# Proteomic Analysis in Nitrogen-Deprived *Isochrysis galbana* during Lipid Accumulation

**DOI:** 10.1371/journal.pone.0082188

**Published:** 2013-12-05

**Authors:** Pingping Song, Ling Li, Jianguo Liu

**Affiliations:** 1 Institute of Oceanology, Chinese Academy of Sciences, Qingdao, China; 2 Graduate University of Chinese Academy of Sciences, Beijing, China; Lawrence Berkeley National Laboratory, United States of America

## Abstract

The differentially co-expressed proteins in N-deprived and N-enriched *I. galbana* were comparatively analyzed by using two dimensional electrophoresis (2-DE) and matrix-assisted laser desorption/ionization-time-of-flight/time-of-flight-mass spectrometry (MALDI-TOF/TOF-MS) with the aim of better understanding lipid metabolism in this oleaginous microalga. Forty-five of the 900 protein spots showed dramatic changes in N-deprived *I. galbana* compared with the N-enriched cells. Of these, 36 protein spots were analyzed and 27 proteins were successfully identified. The identified proteins were classified into seven groups by their molecular functions, including the proteins related to energy production and transformation, substance metabolism, signal transduction, molecular chaperone, transcription and translation, immune defense and cytoskeleton. These altered proteins slowed cell growth and photosynthesis of *I. galbana* directly or indirectly, but at the same time increased lipid accumulation. Eight key enzymes involved in lipid metabolism via different pathways were identified as glyceraldehyde-3-phosphate dehydrogenase (GAPDH), phosphoglycerate kinase (PGK), enolase, aspartate aminotransferase (AST), fumarate hydratase (FH), citrate synthase (CS), O-acetyl-serine lyase (OAS-L) and ATP sulfurylase (ATPS). The results suggested that the glycolytic pathway and citrate transport system might be the main routes for lipid anabolism in N-deprived *I. galbana*, and that the tricarboxylic acid (TCA) cycle, glyoxylate cycle and sulfur assimilation system might be the major pathways involved in lipid catabolism.

## Introduction

Petroleum storage and environmental pollution associated with fossil fuel consumption are two substantial issues that need good solutions for sustainable development of human society. Microalgae are considered to be a potential renewable resource to produce biodiesel, and mass culture of oleaginous microalgae is an important alternative for solving the energy problem [1]. The objective of improving lipid content in microalgae requires a more detailed understanding of the mechanism of lipid metabolism. Nowadays, biochemical methods [2,3] and genetic engineering [4,5] are the two main strategies used in this field of study.

Nutrient limitations, especially nitrogen deprivation, are commonly used biochemical methods for increasing the lipid content in microalgal cultures. When nitrogen content in the algal culture is low, the microalgae reduce their protein synthesis and increase their lipid and carbohydrate accumulation [6,7]. Although nutrient limitation improves lipid production effectively, it also inhibits cell division and photosynthesis at the same time, and leads to a reduction of algal biomass and total oil production [8]. A two-stage culture mode, with first a nutrient-sufficient biomass production phase followed by a lipid induction phase under nutrient deprivation, is considered to be the most efficient approach [9]. 

Based on their sequence homology and some common biochemical characteristics of a number of genes and/or enzymes, isolated from algae and higher plants, that are involved in lipid metabolism, it’s generally believed that the basic pathways of fatty acid and TAG biosynthesis in algae are analogous to higher plants [1]. From genetic research on higher plants, researchers have proposed a genetic engineering strategy to improve the lipid production of microalgae. Through this strategy, four main pathways, including fatty acid synthesis, Kennedy, alternative synthesis of triacylglycerol (TAG) and competitive inhibition of lipid synthesis would be modified.

Acetyl-coenzyme A carboxylase (ACCase) plays a key regulatory role in fatty acid synthesis of plants and animals. Roessler et al.[10] found that ACCase activity and lipid accumulation simultaneously increased in silicon-deprived *Cyclotella cryptic*. Dunahay et al. [11] reported an over-expressed ACCase in *C. cryptic* and *N. saprophila* but its lipid accumulation was not enhanced. Sheehan et al. [12] pointed out that the pathways of lipid biosynthesis may be subject to feedback inhibition, so that the increased activity of ACCase was compensated for by other pathways in the cells and the lipid content wasn’t improved. Fatty acid synthase (FAS) catalyzes the lengthening of carbon chains and is considered to be a crucial multienzymic complex in fatty acid synthesis. Verwoert et al. [13] overexpressed the 3-ketoacyl-acyl carrier protein synthase III (KASIII) of the FAS system in *E. coli*, which resulted in a change of fatty acid composition, but cell growth was seriously blocked and lipid content was not increased. No KASIII overexpression has yet been reported in microalgae.

In the Kennedy pathway, free fatty acids in the endoplasmic reticulum are assembled to form TAG, which is acetyl-CoA dependent. Glycerol-3-phosphate dehydrogenase (G3PDH), glycerol-3- phosphate acyltransferase (GPAT), lysophosphatidic acid acyltransferase (LPAAT) and diacyl-glycerol acyltransferase (DGAT) are four important enzymes in the above process. Vigeolas et al. [14] overexpressed yeast G3PDH in *Brassica napus* seed and found its lipid content was increased by 40%. Moreover, the lipid content in higher plant seeds was significantly improved by over-expression of GPAT [15], LPAAT [16] and DGAT [17,18] genes. For microalgae, GPAT [19] and DGAT2 in *T. pseudonana* and DGAT2 in *O. tauri* [20,21] have been cloned and characterized. However, no improvement of lipid content in the microalgae was reported by over-expression of these enzymes. 

Membrane lipids can also be transformed into TAG [22]. A TAG synthesis pathway independent of ACCase was found in bacteria [23], yeast and plants [24]. Cho et al.[25] reported that the specific acyl-hydrolase activity of galactolipid in nitrogen-deficient *Dunaliella salina* increased, and fatty acids were transformed into TAG. TAG was also regulated by carbohydrate. Roessler [10] found that when *C. cryptica* was exposed to silicon deprivation stress, the activity of chrysolaminarin synthetase was reduced by 31% and the carbon flow to chrysolaminarin decreased from 21.6% to 10.6%. Concomitantly, the carbon flow to lipid increased from 27.6% to 54.1%. Wang et al. [26] reported that the BAFJ5 mutant of *C. reinhardtii* couldn’t produce starch when stressed by nitrogen-deprivation; however, TAG synthesis increased greatly. These results indicate that blocking of carbohydrate synthesis may be an effective way to obtain high oil production in microalgae.

Lipid production is an extremely complicated process involving many metabolic pathways. It is impossible to get maximal lipid production simply by regulating one or two genes. Therefore, more extensive strategies to improve lipid accumulation are expected. Generally, lipid accumulation is regarded as a protective and survival strategy of microalgae under stress, which involves many enzymatic reactions [27]. A large store of information regarding lipid metabolism and homeostasis adjustment can be obtained through proteomic analysis and genome sequencing [28]. Here, we present our results of differentially co-expressed proteins and their roles in the regulation of lipid metabolism in nitrogen-deprived *Isochrysis galbana* so as to provide a new insight into lipid metabolism.

## Materials and Methods

### Strains and culture conditions

**Figure 1:**
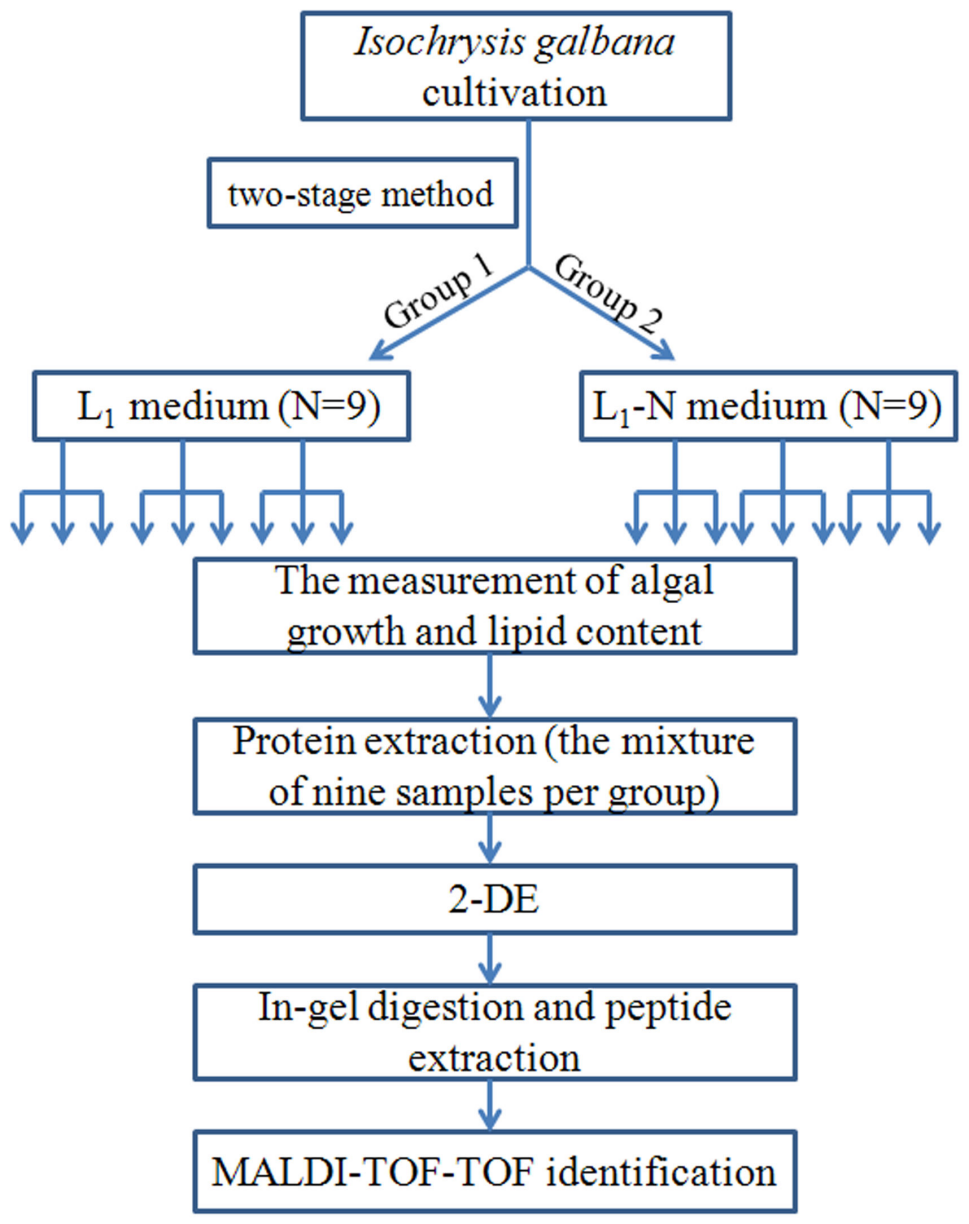
The workflow outlining the proteomic analysis of *I*. *galbana* in N-deprived L_1_ and L_1_ media. To analyze the growth, lipid accumulation and protein variation of *I*. *galbana*, three replicates per group were performed in this experiment, and three samples were took from each replicate. In total, nine samples per group were analyzed. But in the proteomic analysis, the protein of every group was extracted from the mixture of nine samples.

*Isochrysis galbana* IOAC724S was obtained from the R & D Center of Marine Biotechnology, Institute of Oceanology, Chinese Academy of Sciences. Based on the growth and lipid analysis for 52 marine microalgae in our laboratory, this strain of *I. galbana* was considered as a fast-growth oleaginous microalga, with the greatest potential for development as an oil-producing species (unpublished). The two-stage culture method was used for the algal cultivation. The algal cells were first cultured statically for biomass in L_1_ medium [29] at 26°C under 100 μmol photons m^-2^ s^-1^ light intensity with 14:10 h light:dark photoperiod, and manually shaken 6-8 times daily. When the culture reached stationary growth phase, cells (about 8-10×10^6^ cells ml^-1^) were harvested by centrifugation (25°C, 4000 rpm, 6 min). Then, the algal pellet was well suspended in N-free L_1_ medium. Any nutrients remaining in the cell pellets were efficiently removed by washing three times with N-free L_1_ medium. The cell pellet was equally divided into two groups and then inoculated separately in N-free L_1_ and L_1_ medium. The algal cultures were incubated in the above culture conditions, and sampled every 2 days for analysis 3 replicates per group were performed in this experiment, and three samples were taken from each replicate. In total, nine samples per group were analyzed (Figure 1). 

### Cell density and determination of chlorophyll content

Cell density was determined by counting cells with a hemocytometer under a light microscope. The chlorophyll was extracted by methanol and measured photospectrometrically [30]. The total chlorophyll concentration was calculated by using the equation: Chlorophyll (mg/l) = 444. × A_666nm_ + 19,71 × A_653nm._

### Chlorophyll fluorescence induction dynamics

The chlorophyll a fluorescence transients of *I. galbana* both in N-free L_1_ and L_1_ medium were measured with a Handy PEA fluorometer (Hansatech Instruments, Norfolk, UK) according to Strasser et al [31]. All measurements were performed after 15 min dark adaptation at room temperature [32]. 

### Total lipid and fatty acid analysis

Total lipid was extracted in chloroform-methanol (2:1) according to Bligh and Dyer [33]. The fatty acids were released and transesterificated in 2% H_2_SO_4_-methanol solution. Fatty acids were analyzed according to Liu’s gas chromatographic method [34].

### Protein extraction and determination

The nine samples per group were mixed to extract the algal protein using the Trizol method [35] (Figure 1). The obtained protein was solubilized by rehydration for about 4 h, then centrifuged (12,000 rpm, 4°C, 10 min) to remove insoluble substances. The protein concentration of microalgae was determined according to Bradford assay with modifications to alleviate the interference of detergent and reducing agents [36-40].

### Two dimensional electrophoresis (2-DE) and image analysis

The protein samples extracted from *I. galbana* grown in N-free L_1_ and L_1_ medium were used for 2-DE analysis according to Jiang’s method [27]. In detail, 1000 μg of protein extract was disolved in 300 µl rehydration buffer, then loaded onto the IPG strip (pH3-10, nonlinear, 18 cm; GE Healthcare, Sweden). The rehydration buffer contained 7 M urea, 2 M thiourea, 4% CHAPS, 65 mM DTT, 0.2% Bio-Lyte pH 3-10 and 0.001% Bromophenol Blue. The IEF was performed using a Protean IEF Cell with an immobilized pH gradient (Bio-Rad, USA) at 20°C. The voltage was set according to the following programs: 1 h at 500 V, 1 h at 1000 V, 3 h at 8000 V, 24000 Vh at 8000 V and 16 h at 500 V. After IEF, the gel strips were equilibrated with equilibration buffer (6 M urea, 2% SDS, 0.375 M Tris-HCl pH 8.8, 20% glycerol, 2% DTT) for 15 min, then put into another equilibration buffer containing 2.5% iodoacetamide (instead of DTT) for 15 min. For the second dimension electrophoresis, 12% SDS-PAGE was performed on a Bio-Rad Protean II xi Cell System at 14°C. After 2-DE, the gels were fixed for 30 min in fixative solution containing 40% (v/v) methanol and 10% (v/v) acetic acid, then washed four times for 15 min with distilled water, and finally stained in a solution containing 0.12% Coomassie brilliant blue (CBB) G-250, 10% ammonium sulfate, 10% phosphoric acid and 20% methanol. 

The 2-D gels were scanned and Image Master 2D Platinum 7.0 (GeneBio, Geneva, Switzerland) software was applied to analyze the differential protein spots. The amount of a protein spot was calculated according to the spot volume. With a view to the variations induced by the protein loading and staining, the volume of a protein spot was normalized as a percentage of total volume of all protein spots on the gel. The average value ± standard deviation was calculated by the obtained data. The SPSS data processing system was applied to conduct the T-test analysis. A *P*-value < 0.05 was considered to be significant.

### In-gel digestion and peptide extraction

Proteins were subjected to in-gel digestion [41]. Differently co-expressed protein spots were excised from the stained gels and transferred to 1.5 ml centrifuge tubes. They were washed three times using 25 mM NH_4_HCO_3_ and 50% acetonitrile (ACN), and then treated in 10 mM DTT at 56°C for 1 h and in 55 mM IAM for 45 min. Subsequently, in-gel trypsin digestion was performed by adding 10 ng/ml trypsin in 25 mM NH_4_HCO_3_ at 37°C overnight. Finally, 5% methanoic acid was used to stop the reaction. 

### Matrix-assisted laser desorption/ionization-time-of-flight/time-of-flight-mass spectrometry (MALDI-TOF/TOF-MS) and protein identification

After the in-gel digestion and peptide extraction, 1 μl peptide solution was dripped onto the Anchorchip target plate. When the droplet had dried at room temperature, 0.1 μl of matrix solution (CHCA) was dripped onto the plate at the same place. The peptide solution was mixed with the matrix solution (CHCA) on the target plate, dried, and then analyzed by ultrafleXtreme MALDI-TOF-TOF instrument [41]. The plate was loaded into the spectrometer, which was set for reflect mode. The mass range was from 500 to 3500 Da, and the scan resolution was 50, 000. The instrument was operated when the ion source voltage was 25 KV, the reflector voltage was 26.5 KV and the LIFT voltage was 19 KV. The laser from smart beam II was 553 nm and 1000 Hz. Peptide ions were excited and introduced to the mass analyzer for measuring and detected in reflector detector. 1500 hits were accumulated for every spectrum. After the MS scan, the five most abundant MS peaks were selected as parent ions for the MS/MS scans, which were recorded as five files. The MS data were saved in profile format. 

Combined peptide mass fingerprinting (PMF) and MS/MS queries were performed by the MASCOT search engine v2.3.02 against the NCBInr database (year 2011, all entries 16245521 sequences). Maximally one missed tryptic cleavage was allowed; variable Gln-pyro-Glu (N-term Q) @N_term, deamidation (NQ) @1-21, cysteine carbamidomethylation (C) @1-20 and methionine oxidation (M) @1-19 modifications were considered (table S1). Fragment mass tolerance was set to 0.6 Da. Peptide mass tolerance was set at ±100 ppm for MS analysis with a minimum requirement of three peptides matched. Protein identifications were mainly dependent upon PMF matches, a protein score ≥73 was regarded as significant (p<0.05) in this experiment. Several protein identifications were also confirmed by MS/MS; an ion score greater than the significance score 45 (p<0.05) was applied as a cut-off. Although the ion scores of some proteins were lower than the threshold, the ion matches only played a supproting role in protein identifications, and the PMF matches were critical to this. 

## Results

### Cell growth and lipid accumulation in the N-deprived treatment

**Figure 2:**
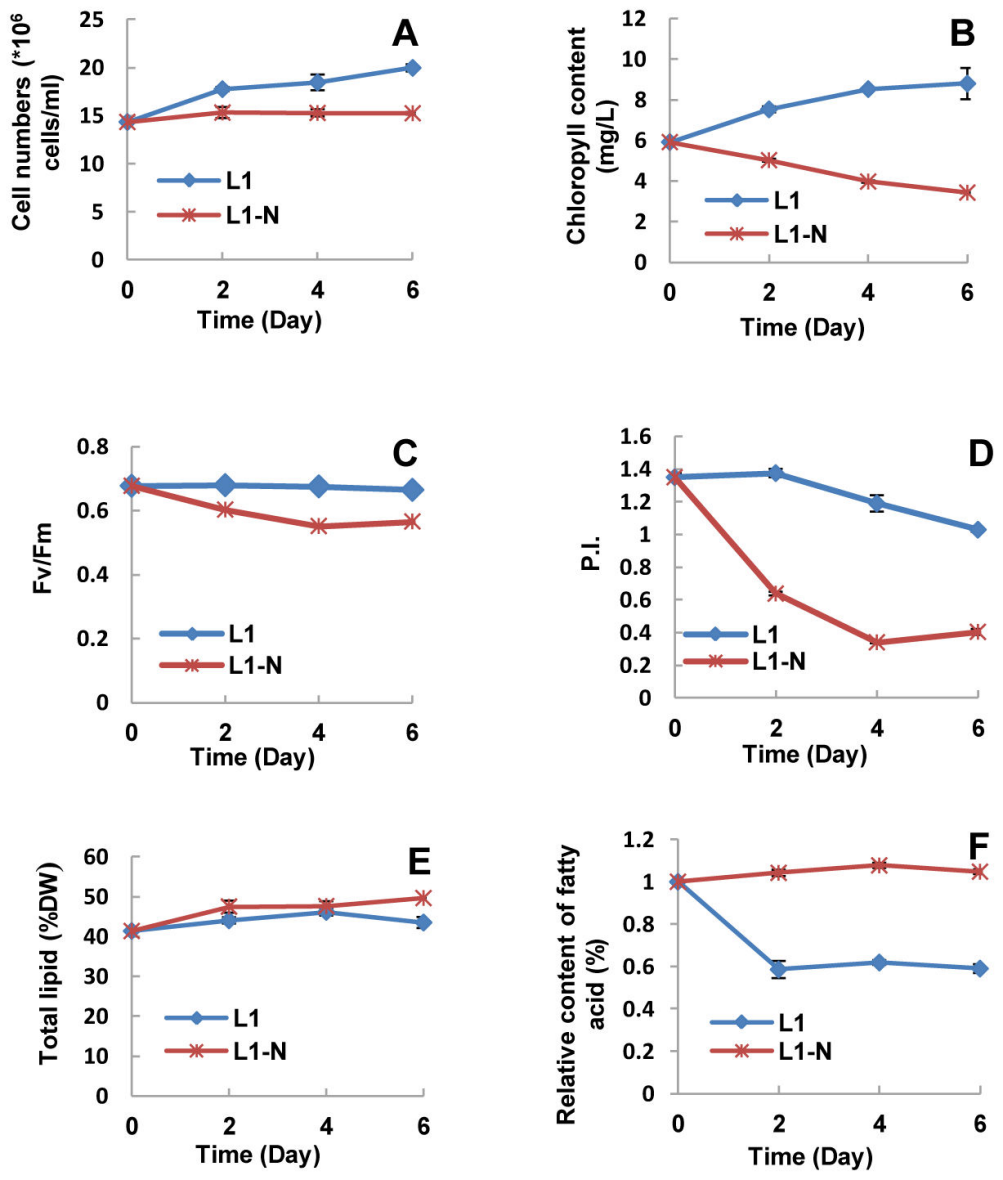
The growth and lipid changes of *I*. *galbana* in L_1_ and N-deprived L_1_ (L_1_-N) media. A, cell numbers; B, chlorophyll; C, Fv/Fm ; D, P.I. ; E, total lipid; F, relative content of fatty acid.

Cell density of *I. galbana* increased gradually from 1.44×10^7^ cells/ml to 2×10^7^ cells/ml in L_1_ medium during the 6-day culture period, but it was stationary at about 1.4-1.5×10^7^ cells/ml in N-deprived medium (Figure 2A, p<0.01). The chlorophyll of *I. galbana* increased from 5.9 mg/L to 7.8 mg/L in L_1_ medium, but decreased from 5.9 mg/L to 2.9 mg/L in N-deprived medium (Figure 2B, p<0.01). 

The Fv/Fm ratio was used to evaluate the maximal efficiency of PSII photochemical activity. Fv/Fm of *I. galbana* in L_1_ medium stayed at a constant high level (~0.7) during the whole culture period, but dropped from 0.7 to 0.55 in N-deprived L_1_ medium (Figure 2C, p<0.01). The performance index (P.I.) can reflect the photosynthetic change, and is more sensitive than Fv/Fm. The P.I. value of *I. galbana* in L_1_ medium slightly decreased from 1.35 to 1.1, whereas it dramatically decreased from 1.35 to 0.4 in N-deprived medium (Figure 2D, p<0.01). 

The total lipid content of cells grown in L_1_ medium increased slightly during the 6-day culture period but increased noticeably from 41% to 49.6% in N-deprived medium (Figure 2E, p<0.05 on the 2^nd^ day, p<0.01 on the 6^th^ day). The relative content of fatty acid remained stable at a high level (~1.0) in N-deprived medium, but showed a dramatic decline to a low level (~0.6) in cells exposed to nutrient-enriched L_1_ medium (Figure 2F, p<0.01). 

### Protein expression and identification

**Figure 3:**
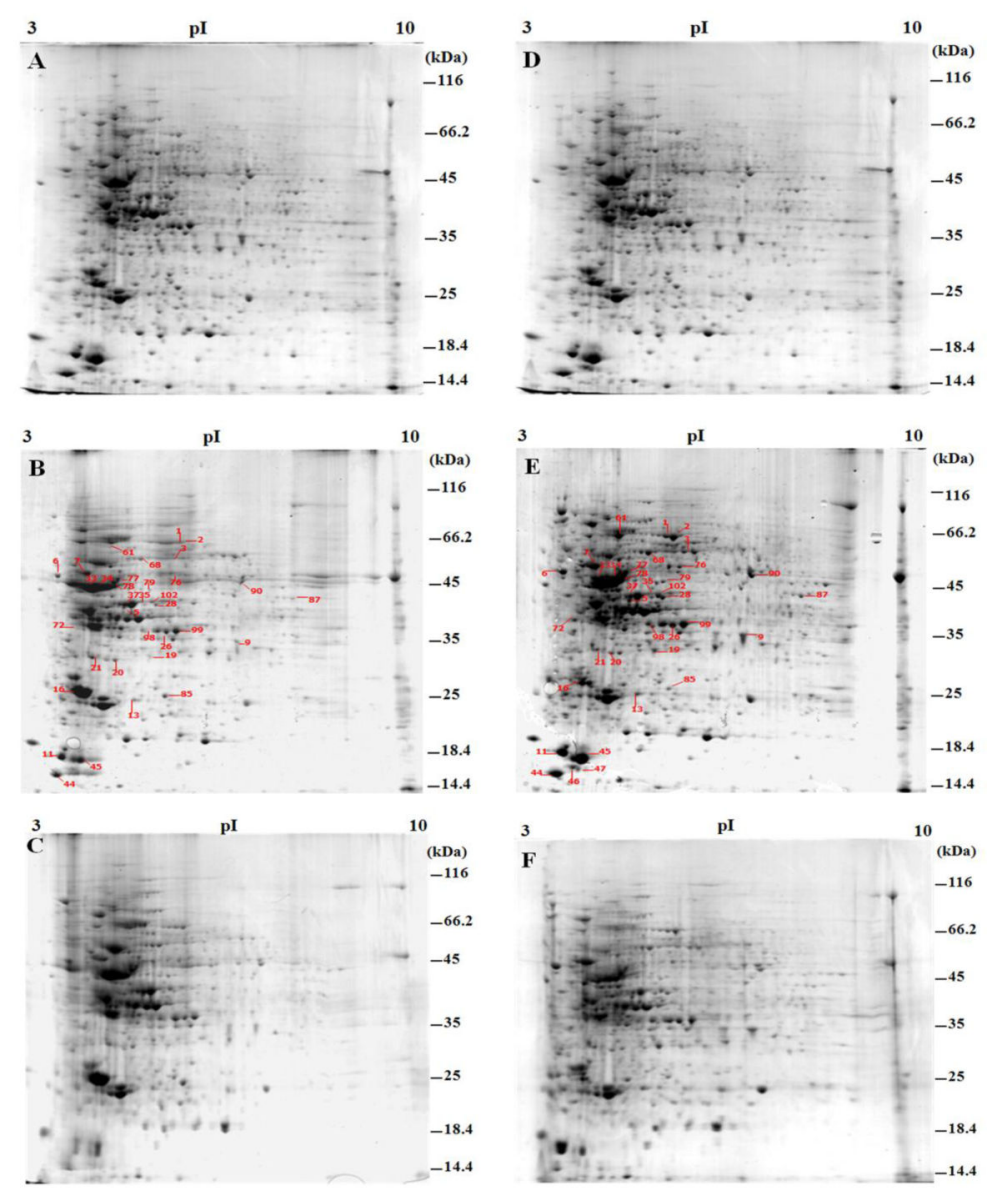
2-DE maps of *I*. *galbana* in N-deprived L_1_ and L_1_ media. (A-C), sample of control collected at day 0, 2 and 6 respectively. (D-F), N-deprived samples for day 0, 2 and 6. The protein samples were separated by SDS-PAGE using a 12% gel, following IEF. The gels were stained with CBB G-250. The straight lines denote the positions of the changed proteins on the 2^nd^ day in N-deprived *I*. *galbana*. The replicate 2DE maps on the 2^nd^ day are seen in the Figure S1.

The soluble proteins of *I. galbana* were separated by 2-DE. In *I. galbana*, the highest content of lipid was obtained on the 6^th^ day, and the protein expression was reported to be earlier than the lipid accumulation in some nitrogen-deprived microalgae strains [42], so three time points (day 0, 2 and 6) were selected for proteomic analysis in this study. The 2-DE maps (Figure 3) showed that the most significant proteomic variations happened on the 2^nd^ day, and more kinds of proteins were expressed in N-deprived *I. galbana* than nutrient-enriched L_1_ group. Therefore 2-DE gels for the 2^nd^ day were selected for further analysis using ImageMaster 2D software. More than 900 protein spots were detected on the gels. Forty-five of the 900 protein spots showed an obvious differential expression in N-deprived *I. galbana* compared with that of the control. Among the protein spots analyzed for N-deprived *I. galbana*, 41 proteins showed up-regulated expression (expression levels ≥ 1.5-fold of controls), and 4 showed down-regulated expression (expression levels ≤ 0.55-fold of controls).

 These differentially expressed protein spots for N-deprived *I. galbana* were further analyzed by MALDI-TOF/TOF-MS. Based on the NCBI database and Mascot search engine, additional and more accurate peptide identifications were achieved. Thirty-six of the differentially expressed protein spots showed apparent similarities with proteins in the searched database, including 32 up-regulated protein spots (representing 23 proteins) and 4 down-regulated protein spots (representing 4 proteins). The remaining 9 protein spots had no hits in the searched database. In some cases, more than one protein spot was identified for the same protein, which might be due to different protein isoforms or post-translational modifications [27]. The identified proteins were divided by their molecular functions into seven categories as follows (Table 1): energy production and transformation (13 spots), substance metabolism (12 spots), signal transduction (1 spot), molecular chaperone (2 spots), transcription and translation (3 spots), immune defense (2 spots) and cytoskeleton (3 spots). More information on all the proteins identified from the differentially expressed spots obtained for the 2^nd^ day cultures are described below.

**Table 1:** Differentially expressed proteins in N-deprived *I. galbana* on the 2^nd^ day.

Spot no.		Protein ID	Protein (species)	Predicted MW(kDa)/pI		Observed MW(kDa)/pI	SC (%)	Protein Score/NMP	N-deprivation /control mean±SD
**The proteins related to energy generation and transformation**									
33	XP_001691632.1		ATP synthase subunit beta (*Phytophthora infestans* )	62/4.73		46/4.70	10.3	167/3	2.31±0.25
90	EGI63505.1		ATP synthase subunit alpha (*Acromyrmex echinatior*)	59.6/9.34		50/6.80	31	343/17	2.62±0.13
9	AAW79315.1		chloroplast ferredoxin NADP(+) reductase (*Isochrysis galbana*)	40.8/7.71		37/5.96	14.7	141/8	1.51±0.32
34	XP_002998682.1		ATP synthase subunit beta (*Phytophthora infestans*)	50.7/5.21		46/4.96	10	222/3	1.95±0.31
78	BAF38479.1		H+-ATPase subunit B (*Zostera marina*)	54.5/4.94		47/4.99	24.6	347/12	2.17±0.03
9	CAD27443.1		vacuolar ATPase subunit B	54.2/4.69		47/5.53	37.8	177/17	2.88±0.06
44	ABA55531.1		chloroplast light harvesting protein isoform 15 (*Isochrysis galbana*)	21.9/4.25		16.3/3.90	10	154/4	2.05±0.68
46	ABA55520.1		chloroplast light harvesting protein isoform 4 (*Isochrysis galbana*)	23/4.68		16/4.51	17.8	78/5	2.67±0.2
47	CBI83417.1		light-harvesting protein (*Symbiodinium* *sp.*)	26.8/9.4		16/4.60	25.1	92/5	3.83±0.19
13	BAJ61707.1		oxygen-evolving enhancer protein (*Neorhodella cyanea*)	37/5.01		28/5.40	18.8	87/5	2.18±0.06
1	XP_003064321.1		glutamine synthetase(*Micromonas pusilla*)	75/5.74		72/5.60	24.3	94/11	2.24±0.05
2	XP_003064321.1		glutamine synthetase (*Micromonas pusilla*)	75.4/5.74		72/5.70	15.4	97/10	4.06±0.64
16*	YP_002834016.1		inosine-5'-monophosphate dehydrogenase (*Corynebacterium aurimucosum*)	53.4/5.25		33.50/4.5	25.6	88/16	0.13±0.01
**The proteins related to substance metabolism**									
21*		BAB47124.1	ACC synthase (*Cucurbita maxima*)	55/7.24		34/4.60	46.4	87.5/22	0.52±0.04
11		AAW79325.1	phosphoglycerate kinase (*Isochrysis galbana*)	46.3/4.78		20/4.25	22	130/10	2.07±0.17
35		AAW79326.1	phosphoglycerate kinase (*Isochrysis galbana*)	48.9/7.89		43/5.50	16.7	123/6	4.38±0.23
28		AAW79327.1	phosphoglycerate kinase (*Pavlova lutheri*)	44.4/5.97		42/5.60	23.4	137/8	2.11±0.11
7		AAR97551.1	enolase (*Phaeodactylum tricornutum*)	41.3/4.3		49/4.55	12.7	156/3	1.78±0.03
77		CAB75428.1	enolase (*Lupinus luteus*)	47.9/4.89		47/4.96	32.7	161/14	1.51±0.39
68		YP_562918.1	fumarate subfamily Fe-S type hydro-lyase alpha subunit (*Shewanella denitrificans*)	56.1/5.79		55/5.40	21.5	94/10	2.04±0.21
9		AAD01872.1	glyceraldehyde-3-phosphate dehydrogenase (*Gonyaulax polyedra*)	37/6.31		34/6.60	27.49	137/10	4.22±0.31
45		BAD05063.1	citrate synthase (*Tetrahymena thermophila*)	55/8.81		18/4.60	28.5	75/14	2.01±0.06
85*		AAB68396.1	aspartate aminotransferase 2 precursor (Canavalia lineata)	51/8.82		30/5.80	35.3	77/19	0.36±0.02
98		ABA55516.1	chloroplast O-acetyl-serine lyase (*Isochrysis galbana*)	21/5.04		34/5.50	24.9	76/5	2.76±0.04
5		ABA55503.1	chloroplast ATP sulfurylase (*Isochrysis galbana*)	23.7/8.12		40/5.20	16.4	89/4	2.17±0.53
**The proteins related to signal transduction**									
6		ZP_01437860.1	two-component sensor histidine kinase (*Fulvimarina pelagi*)		49.1/7.33	52/4.10	23.1	92/15	1.99±0.16
**The proteins related to molecular chaperone**									
61		EGB12199.1	heat shock protein 70 (*Aureococcus anophagefferens*)	71.9/4.73		71/4.85	19	181/8	2.6±0.09
102		XP_002901832.1	T-complex protein 1 (*Phytophthora infestans*)	62.6/6.84		44/5.50	36.4	82.4/23	1.61±0.34
**The proteins related to transcription and translation**									
87		NP_001185317.1	transcription elongation factor SPT6 (*Arabidopsis thaliana*)	167/6.14		43/5.56	28.9	75.9/36	2.1±0.17
20		AEB21848.1	ribosomal protein S3 (*Zoopsis liukiuensis*)	43.4/10.57		33/4.93	54.4	75.1/20	2±0.46
37*		AAV34146.1	elongation factor 1α (*Isochrysis galbana*)	53/9.12		43/5.20	29	201/14	0.55±0.04
**The proteins related to immune defense**									
76		AAY46275.1	beta globin chain (Homo sapiens)	11.5/6.34		55/5.75	53.3	170/5	2.08±0.48
26		ACP30568.1	disease resistance protein (*Brassica rapa* *subsp*)	118/7.94		37/5.70	34.5	78/33	1.53±0.19
**The proteins related to cytoskeleton**									
72		ABG00024.1	Myosin head family protein (*Oryza sativa*)	166/7.44		37/4.40	25.1	74/35	2.89±0.18
19		XP_003078192.1	Myosin class II heavy chain (*Ostreococcus tauri*)	304/4.74		30/5.50	25.5	77/64	1.83±0.13
3		XP_003063693.1	inner dynein arm heavy chain 1-beta (*Micromonas pusilla*)	489/5.52		60/5.80	18.8	79.4/65	2.21±0.16

NMP is the number of peptides matched. SC is sequence coverage. Asterisk (*) denotes down-regulated proteins.

#### Energy generation and transformation

Protein spots 33, 34, 78, 79 and 90 were classified into the ATP synthase family (Figure 3, Table 1), and were further identified as ATP synthase subunit beta, ATP synthase subunit alpha, ATP synthase subunit beta, H^+^-ATPase subunit B and vacuolar ATPase subunit B, respectively. About a 2 to 3-fold up-regulated expression of all these proteins was found in N-deprived *I. galbana*. ATP synthase, the main enzyme catalyzing ATP synthesis in eukaryotic cells, plays an important role in oxidative and photosynthetic phosphorylation. 

Spots 44 and 46 were identified as chloroplast light harvesting protein 15 and 4, and spot 47 was determined to be another chloroplast light harvesting protein (Figure 3, Table 1). Their expressions were up-regulated about 2-4 fold in N-deprived *I. galbana* on the 2^nd^ day of culture. The light-harvesting protein can bind pigment into a pigment-protein complex that captures light energy and transmits the energy to the reaction center for inducing a photochemical reaction. Moreover, it plays an important role in regulating the distribution of excitation energy, photoprotection and environmental adaptation [43]. 

Spot 99 was identified as chloroplast ferredoxin NADP^+^ reductase (Figure 3, Table 1). Expression of this protein in N-deprived *I. galbana* was up-regulated, with about a 1.5-fold increase occurring on the 2^nd^ day. Fd-NADP^+^ reductase catalyzes the redox reaction from Fd to NADP^+^ in chloroplasts, which can regulate the electron transfer of photo-reduction and provide the reducing power for carbon assimilation. 

 Spot 13 was identified as oxygen-evolving enhancer protein (Figure 3, Table 1). Expression of this protein was up-regulated, with about a 2-fold increase in *I. galbana* on the 2^nd^ day of N-deprivation. Oxygen-evolving enhancer protein, a subunit of the oxygen-evolving complex (OEC) of PSII in the chloroplast [44], plays an important role in the primary reaction of photosynthesis. When the plant is exposed to stress, the OEC proteins degrade to help adapt to the adverse environment, with the oxygen-evolving enhancer protein being the degradation product of these proteins [45]. 

Spots 1 and 2 were determined to be glutamine synthetase (GS, Figure 3, Table 1). Their expressions in N-deprived *I. galbana* were about 2-4 fold up-regulated. GS catalyzes the reaction of ammonia and glutamic acid into glutamine. 

Spot 16 was identified as inosine-5'-monophosphate dehydrogenase (IMPD, Figure 3, Table 1). Expression of IMPD in N-deprived *I. galbana* was down-regulated to 1/8 of its control value. IMPD catalyzes the conversion of inosine monophosphate (IMP) into xanthosine monophosphate (XMP), which can then be converted into guanosine monophosphate (GMP). 

#### Substance metabolism

Spots 11, 35 and 28 were classified as phosphoglycerate kinase (PGK, Figure 3, Table 1). Their expressions were up-regulated, with about a 2-4 fold increase in N-deprived *I. galbana* on the 2^nd^ day. PGK catalyzes 1,3-bisphosphoglycerate (1,3-BPG) into 3-phosphoglycerate (3-PG). 

Spots 7 and 77 were identified as enolase (Figure 3, Table 1). The expressions of these proteins were 1.5-2 fold up-regulated in N-deprived *I. galbana*. Enolase catalyzes 2-PG into phosphoenol-pyruvate (PEP). 

Spot 9 was determined to be glyceraldehyde-3-phosphate dehydrogenase (GAPDH). Expression of GAPDH was up-regulated, with about a 4-fold increase in N-deprived *I. galbana*. GAPDH not only catalyzes dihydroxyacetone phosphate (DHAP) into 3-phosphoglycerol (3-PG), but also catalyzes glyceraldehyde-3- phosphate (GAP) into 1,3-BPG. 

 Spot 45 was identified as citrate synthase (CS), with about a 2-fold up-regulated expression in N-deprived *I. galbana* on the 2^nd^ day. CS catalyzes the reaction of acetyl-CoA and oxaloacetic acid into citric acid and CoA in the TCA cycle. CS has various isozymes, whose activity can cause variation of the acetyl-CoA pool directly, as well as affect fatty acid synthesis [46,47]. 

Spot 68 was identified as the Fe-S type hydro-lyase alpha subunit of fumarate hydratase (FH). Its expression was up-regulated about 2-fold in N-deprived *I. galbana* (Figure 3, Table 1). FH catalyzes the conversion of fumaric acid into malic acid in the TCA cycle [48]. 

 Spot 98 was shown to be chloroplast O-acetyl-serine lyase (OAS-L) (Figure 3, Table 1), which had about a 3-fold up-regulated expression in the 2^nd^ day cultures of N-deprived *I. galbana*. OAS-L is the key enzyme in cysteine synthesis, and catalyzes the reaction of sulphide and acetyl serine into cysteine.

Spot 5 were determined to be ATP sulfurylase (ATPS) in the chloroplast, with about a 2-fold up-regulated expression in the 2^nd^ day cultures of N-deprived *I. galbana*. ATPS catalyzes the reaction of adenosine phosphosulfate and pyrophosphate into ATP and sulfate [49,50]. 

Spot 21 was identified as 1-aminocyclopropane-1-carboxylic acid (ACC) synthase (Figure 3, Table 1). The expression of ACC synthase in N-deprived *I. galbana* was half that of the control for the 2^nd^ day cultures. ACC synthase, the rate-limiting enzyme in ethylene (ETH) biosynthesis, catalyzes the reaction of S-adenosyl methionine (SAM) into ACC.

Spot 85 was identified as the precursor of aspartate aminotransferase (AST) 2 (Figure 3, Table 1). AST expression in N-deprived *I. galbana* was only 40% that of the control on the 2^nd^ day. AST catalyzes the reversible interconversion of oxaloacetic acid and aspartic acid, thus performs the transport through the membrane from mitochondrion to glyoxysome.

#### Signal transduction

Spot 6 was identified as the two-component sensor histidine kinase (Figure 3, Table 1). The up-regulated expression of this protein in the 2^nd^ day N-deprived *I. galbana* was about 2-fold higher than that of N-enriched cultures. Histidine kinase can catalyze ATP-dependent auto-phosphorylation, which plays roles in regulating metabolism, genetic expression and cell growth [51]. 

#### Molecular chaperone

Spots 61 and 102 were classified into the family of heat shock proteins (HSP) and identified as HSP 70 and T-complex protein 1 (Figure 3, Table 1). Their expressions were up-regulated about 2.5 and 1.5-fold respectively in N-deprived *I. galbana*. T-complex protein 1 belongs to the HSP60 family. HSP, as a molecular chaperone, helps the proteins refolded correctly, and participates in cell apoptosis and protein metabolism. 

#### Transcription and translation

Spots 87 and 20 was identified as transcription elongation factor SPT6 and ribosomal protein S3, respectively (Figure 3, Table 1). Their expressions were 2-fold up-regulated in N-deprived *I. galbana*. The transcription elongation factors can influence transcriptional efficiency by restraining or otherwise affecting the momentary pausing and arrest of RNA polymerase II (RNApol-II) during the process of transcription [52]. Ribosomal protein S3, a protein in the small ribosomal subunit, is involved in protein synthesis, cellular division and differentiation. 

Spot 37 was determined to be elongation factor 1α (Figure 3, Table 1). Expression of this protein was down-regulated in N-deprived *I. galbana* to about half the level of the control. Translation elongation factors are proteins that facilitate the elongation of peptide chains during protein synthesis. 

#### Immune defense

Spots 26 and 76 were identified as disease resistance protein and beta globin chain (Figure 3, Table 1). Their expressions in N-deprived *I. galbana* were up-regulated ca 1.5 to 2-fold on the 2^nd^ day. Globins are major immune molecules that counteract pathogens. The disease-resistance protein can specifically recognize pathogenic bacteria and stimulate a strong disease reaction in plant cells. 

#### Cytoskeleton

Spots 72, 19 and 76 were determined to be myosin head family protein, myosin class II heavy chain and inner dynein arm heavy chain 1-beta, respectively (Figure 3, Table 1). Their expression was up-regulated about 2 to 3-fold in N-deprived *I. galbana*. Myosin participates in muscle contraction, material transport and morphological changes of cells. Dynein participates mainly in reversible substance transportation for flagella. Myosin and dynein, the main molecular motors, transform chemical energy into mechanical energy by ATP or GTP hydrolysis [53]. 

## Discussion

Nutrition limitation can improve lipid production effectively, but it also inhibits cell division and photosynthesis [8]. These observations were also evident in our studies on N-deprived and N-enriched *I. galbana*. When this oleaginous alga was exposed to nitrogen deprivation, its cell growth was obviously slowed (Figure 2A), and its photosynthesis was seriously inhibited, with a substantial decrease in Fv/Fm (Figure 2C) and P.I. (Figure 2D). Concomitantly, the total lipid content (Figure 2E) and proportion of fatty acids (Figure 2F) in the biomass increased. The above physiological changes necessarily involved various enzymatic defense reactions in the organism. Proteomic analysis revealed up-regulated and down-regulated proteins in N-deprived *I. galbana* (Figure 3). There were many up-regulated proteins in N-deprived *I. galbana*, of which 27 were identified (Table 1), including ATP synthase, Fd-NADP^+^ reductase, light-harvesting protein, oxygen-evolving enhancer protein, PGK, GAPDH, enolase, CS, FH, ATPS, OAS-L, GS and others. The down-regulated proteins in N-deprived algae were AST, ACC synthase, EF1α and IMPD (Table 1). All these affected proteins might influence cell growth, photosynthesis and lipid accumulation in N-deprived *I. galbana* directly and/or indirectly via several metabolic pathways.

Nitrogen, the major necessary nutrient element, is required for cell growth and chloroplast formation. N-deprivation significantly affected protein synthesis in this alga and resulted in reduction of chlorophyll synthesis (Figure 2B). Cellular division was slowed and photosynthesis was decreased in the N-deprived algae [54,55]. In response to the decrease in photosynthesis, the expression of the light-harvesting protein, oxygen-evolving enhancer protein, ATP synthase and Fd-NADP^+^ reductase in the photosynthetic electron transport system were all actively up-regulated, which might improve the ability of light absorption and transformation, electron transport, and ATP and NADPH production, respectively. The up-regulation of these four proteins at least partly, if not fully, compensated for the decrease in light absorption, the reduction of oxygen-evolution and ATP production.

**Figure 4:**
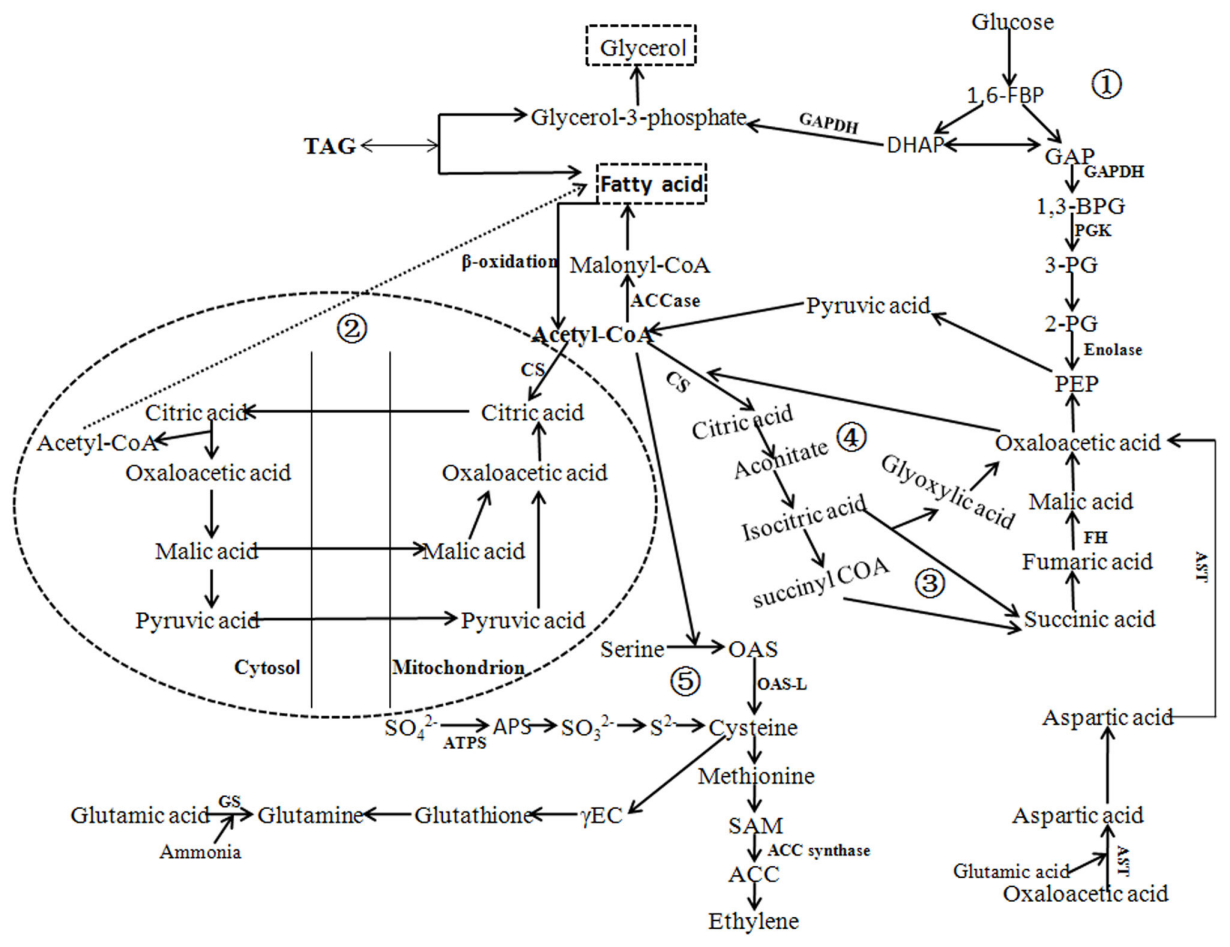
The hypothesized pathways for lipid metabolism in N-deprived *I*. ***galbana***: ① the glycolytic pathway, and involved enzymes GAPDH, PGK and enolase. the citrate transport system, and the involved enzyme CS. the TCA cycle, and the involved enzymes CS and FH. the glyoxylate cycle, and the involved enzymes AST, CS and FH. the pathways of cysteine (sulfur assimilation), glutathione and ethylene biosynthesis, and the involved enzymes OAS-L, ATPS, GS and ACC synthase.

The N-deprivation necessarily resulted in a decrease of Rubisco synthesis [56,57], so the surplus NADPH from the electron transport chain couldn’t be efficiently consumed by the Calvin cycle. The surplus NADPH could be used for fatty acid synthesis as indicated in Figure 2F. Under N-deprived conditions, some crucial proteins for fatty acid synthesis in *I. galbana* were indeed more highly expressed. PGK, GAPDH and enolase are key enzymes in the glycolytic pathway (Fig. 4①), their up-regulation in N-deprived *I. galbana* could increase the transformation of pyruvic acid into acetyl-CoA, and ultimately augment fatty acid synthesis. CS and FH are two key enzymes in the citrate transport system and tricarboxylic acid (TCA) cycle. Their expression was also obviously increased in N-deprived *I. galbana*. Acetyl-CoA is utilized to produce energy in the TCA cycle (Fig. 4③), and the up-regulation of CS and FH could promote the catabolism of fatty acids. However, acetyl-CoA in the mitochondria must be transferred to the cytosol by the citrate transport system (Fig. 4②), and the up-regulation of CS could promote fatty acid synthesis. The five enzymes in the glycolytic pathway and citrate transport system might positively regulate fatty acid synthesis, so the fatty acid content in N-deprived *I. galbana* was clearly increased. The two pathways promoting fatty acid synthesis consumed the surplus NADPH, which relieved the excessively oxidative electron transport chain. Furthermore, the up-regulation of GAPDH could also promote the production of glycerol. The increase of glycerol and fatty acid would promote triacylglycerol (TAG) synthesis (Figure 4). TAG accumulation played an important role in consuming excessive electrons from the electron transport chain [58]. In the glyoxylic acid cycle (Fig. 4④), stored TAG was used to provide the energy required for the oxaloacetic acid and acetyl-CoA to be transformed into glucose. The down-regulation of AST suggested that the lipid catabolism by the glyoxylic pathway might be restrained in N-deprived *I. galbana*.

N-deprivation can also result in a metabolic imbalance of reactive oxygen species (ROS) and an excess of oxygen radicals [59]. Some sulfocompounds (such as glutathione) can quench free radicals, thus improving the resistance of plants [60]. The up-regulation of ATPS and OAS-L suggested that the sulfur assimilation pathway was enhanced in N-deprived *I. galbana*, which could increase the formation of cysteine and finally promoted glutathione synthesis (Fig. 4⑤). According to the demand-driven principle of sulfur nutrition in plants, when sulfur supply is normal, the sulfur assimilation pathway is restrained [61]. When sulfur supply cannot meet the demand, absorption and assimilation of sulfur nutrients is expected to be enhanced, and the expression of OAS-L is up-regulated. The activity of ATPS is negatively regulated by sulfur nutrients, which increase under sulfur-deprived conditions, but decrease when sulfur supply is sufficient [49]. Under N-deprived conditions, *I. galbana* needs a large amount of reductants to quench the free radicals, and consequently the organism activates the sulfur assimilation pathways to increase synthesis of sulfur compounds. In the process, the acetyl-CoA is utilized, so lipid catabolism could be also performed by sulfur assimilation in N-deprived *I. galbana*. Moreover, glutamine is the precursor substance of glutathione synthesis, so the up-regulation of GS could promote glutamine synthesis, which would positively regulate glutathione metabolism (Fig. 4⑤). However, ETH synthesis needs cysteine, which might affect the synthesis of sulfocompounds, so the down-regulation of ACC synthase would slow the activity of the pathway for ETH synthesis from cysteine to ACC (Fig. 4⑤).

It was found that some proteins actively participated in energy metabolism under N-deprivation. The up-regulation of ATP synthase and CS suggested that aerobic respiration might be enhanced in N-deprived *I. galbana*. Aerobic respiration is normally the most effective and main pathway for producing energy, which can make ATP available for the organism. Moreover, the up-regulation of GS and down-regulation of IMPD in purine nucleotide metabolism could restrain the formation of GMP and promote the formation of AMP, and finally increase ATP synthesis. Under N-deprived conditions, the metabolic pattern in *I. galbana* was readjusted. For example, many metabolic pathways producing energy were enhanced in order to adapt to the energy shortage, such that ATP demand and supply reached a new balance. Actually, these protective strategies could not totally remedy the energy shortage, only maintain the energy cycling at a lower level. The down-regulation of EF1α proved that N-deprived *I. galbana* might be in an anti-apoptotic state. The apoptotic signal induced by nutrition depletion can regulate the expression of EF1α. The EF1α was shown to be increased in cell death, and decreased in the anti-apoptotic state [62].

Furthermore, 2DE maps showed that more kinds of proteins in N-deprived *I. galbana* were expressed than nutrient-enriched L_1_ group on the 2^nd^ day. We speculated that, the protein expression pattern of *I. galbana* reflected a stressful and protective mechanism in the initial stage of N-deprivation, so the proteins that were related to lipid synthesis in the stress were induced to express in N-deprived *I. galbana* [1,63,64]. After a longer N-deprivation, the algal cells might change the control strategy for these protein, and the expression of some proteins decreased on the 6^th^ day (Figure 3F), which reflected the adaptive mechanism of *I. galbana*.

## Conclusion

Various metabolic networks are interrelated and interact with each other in cells. Under N-deprived conditions, the production and activity of many proteins involved in nucleic acid, lipid, amino acid and glucose metabolism were changed, which then affected the growth and metabolism of *I. galbana*. The results from proteomic analysis suggested that the glycolysis and citrate transport systems might be the main pathways of lipid anabolism, and that the TCA cycle, the glyoxylate cycle and sulfur assimilation system might be the main pathways of lipid catabolism. GAPDH, PGK, enolase, AST, FH, CS, OAS-L and ATPS were involved in lipid metabolism. Moreover, ATP synthase, Fd-NADP^+^ reductase, light-harvesting protein, oxygen-evolving enhancer protein, GS and IMPD played important roles in regulating energy metabolism. These proteins helped maintain the metabolic and energy balance of *I. galbana* under N-deprived conditions. By analyzing the functions and metabolic pathways in which these proteins are active, more detailed information on lipid metabolism and homeostasis adjustment can be obtained, which adds to the information that can be obtained from just the genome sequence.

## Supporting Information

Figure S1 **The replicate 2-DE maps of *I. galbana* in N-deprived L_1_ and L_1_ media.**
(RAR)

Table S1 **The matched peptides information for differential gel spots of N-deprived *I. galbana* on the 2^nd^ day by the MASCOT software.**
(DOC)
