# Supplementary material for: Proteomic Analysis in Nitrogen-Deprived Isochrysis galbana during Lipid Accumulation
Source: PLoS One. 2013 Dec 5;8(12):e82188. doi: 10.1371/journal.pone.0082188 (PMC3855430; doi:10.1371/journal.pone.0082188)
Supplement: Table S1 — The matched peptides information for differential gel spots of N-deprived I. galbana on the 2nd day by the MASCOT software. (DOC) [file pone.0082188.s002.doc]

**Table S1** The matched peptides information for differential gel spots of N-deprived *I. galbana* on the 2^nd^ day by the MASCOT software.

| Spot no. | Protein ID | Start | End | Observed | Mr (expt) | Mr (calc) | Delta | Miss | Ion Score | Protein Score | Modification | Sequence |
| --- | --- | --- | --- | --- | --- | --- | --- | --- | --- | --- | --- | --- |
| 33 | XP_002998682.1 | 208 | 222 | 1573.77 | 1572.77 | 1572.77 | -0.0048 | 0 |  | 167 | - | AALVYGQMNEPPGAR |
| 63 | 77 | 1677.92 | 1676.92 | 1676.92 | -0.0045 | 0 | 80.14 |  | - | [IVLEVAQHLGENTVR](../../%C3%A6%C2%A1%C5%92%C3%A9%C2%9D%C2%A2/%C3%A6%C2%A1%C5%92%C3%A9%C2%9D%C2%A2%C3%A6%C2%9D%C2%90%C3%A6%E2%80%93%E2%84%A2/%C3%A8%C2%B4%C2%A8%C3%A8%C2%B0%C2%B1%C3%A9%E2%80%B0%C2%B4%C3%A5%C2%AE%C5%A1%C3%A7%5C%C2%BB%E2%80%9C%C3%A6%C2%9E%C5%93/F12FTSECKJ0265_005_GS_20120821/PMF_Report%C3%A5%C2%BF%E2%80%A6%C3%A7%C5%93%E2%80%B9/peptides%C3%A5%C2%BF%E2%80%A6%C3%A7%C5%93%E2%80%B9/33-3.html#23) |
| 238 | 253 | 1878.95 | 1877.95 | 1877.95 | -0.0066 | 0 | 159.95 |  | - | [DVEGQDVLLFVDNIFR](../../%C3%A6%C2%A1%C5%92%C3%A9%C2%9D%C2%A2/%C3%A6%C2%A1%C5%92%C3%A9%C2%9D%C2%A2%C3%A6%C2%9D%C2%90%C3%A6%E2%80%93%E2%84%A2/%C3%A8%C2%B4%C2%A8%C3%A8%C2%B0%C2%B1%C3%A9%E2%80%B0%C2%B4%C3%A5%C2%AE%C5%A1%C3%A7%5C%C2%BB%E2%80%9C%C3%A6%C2%9E%C5%93/F12FTSECKJ0265_005_GS_20120821/PMF_Report%C3%A5%C2%BF%E2%80%A6%C3%A7%C5%93%E2%80%B9/peptides%C3%A5%C2%BF%E2%80%A6%C3%A7%C5%93%E2%80%B9/33-3.html#26) |
| 90 | EGI63505.1 | 310 | 315 | 707.37 | 706.36 | 706.38 | -0.0172 | 0 |  | 343 | - | QAVAYR |
| 201 | 207 | 815.44 | 814.44 | 814.45 | -0.0196 | 0 |  |  | - | ELIIGDR |
| 316 | 322 | 843.44 | 842.44 | 842.47 | -0.0317 | 0 |  |  | Gln->pyro-Glu (N-term Q)@N  _term | QMSLLLR |
| 316 | 322 | 860.48 | 859.47 | 859.45 | 0.0226 | 0 |  |  | Gln->pyro-Glu (N-term Q)@N  _term; Deamidated (NQ)@1; Oxidation (M)@2 | QMSLLLR |
| 316 | 322 | 876.47 | 875.47 | 875.49 | -0.0228 | 0 |  |  | Oxidation (M)@2 | QMSLLLR |
| 428 | 434 | 892.47 | 891.46 | 891.48 | -0.0212 | 0 |  |  | - | LELAQYR |
| 180 | 187 | 903.48 | 902.48 | 902.45 | 0.0224 | 0 |  |  | - | EPMQTGIK |
| 188 | 197 | 1026.60 | 1025.59 | 1025.59 | 0.0044 | 0 |  |  | - | AVDSLVPIGR |
| 41 | 51 | 1217.66 | 1216.66 | 1216.63 | 0.0270 | 0 |  |  | - | SAEISAILEER |
| 212 | 223 | 1286.68 | 1285.68 | 1285.72 | -0.0486 | 0 |  |  | - | TALAIDSIINQK |
| 510 | 521 | 1289.63 | 1288.62 | 1288.69 | -0.0667 | 0 |  |  | Deamidated (NQ)@3 | STQQDLLATIAK |
| 316 | 327 | 1406.68 | 1405.68 | 1405.80 | -0.1220 | 1 |  |  | Gln->pyro-Glu (N-term Q)@N  _term | QMSLLLRRPPGR |
| 396 | 409 | 1438.80 | 1437.79 | 1437.84 | -0.0480 | 0 | 83.12 |  | - | [GIRPAINVGLSVSR](../../%C3%A6%C2%A1%C5%92%C3%A9%EF%86%9D%C2%A2/%C3%A6%C2%A1%C5%92%C3%A9%C2%9D%C2%A2%C3%A6%C2%9D%C2%90%C3%A6%E2%80%93%E2%84%A2/%C3%A8%C2%B4%C2%A8%C3%A8%C2%B0%C2%B1%C3%A9%E2%80%B0%C2%B4%C3%A5%C2%AE%C5%A1%C3%A7%5C%C2%BB%E2%80%9C%C3%A6%C2%9E%C5%93/F12FTSECKJ0265_005_GS_20120821/PMF_Report%C3%A5%C2%BF%E2%80%A6%C3%A7%C5%93%E2%80%B9/peptides%C3%A5%C2%BF%E2%80%A6%C3%A7%C5%93%E2%80%B9/90-2.html#43) |
| 328 | 340 | 1553.70 | 1552.69 | 1552.73 | -0.0420 | 0 | 127.81 |  | - | [EAYPGDVFYLHSR](../../%C3%A6%C2%A1%C5%92%C3%A9%C2%9D%C2%A2/%C3%A6%C2%A1%C5%92%C3%A9%C2%9D%C2%A2%C3%A6%C2%9D%C2%90%C3%A6%E2%80%93%E2%84%A2/%C3%A8%C2%B4%C2%A8%C3%A8%C2%B0%C2%B1%C3%A9%E2%80%B0%C2%B4%C3%A5%C2%AE%C5%A1%C3%A7%5C%C2%BB%E2%80%9C%C3%A6%C2%9E%C5%93/F12FTSECKJ0265_005_GS_20120821/PMF_Report%C3%A5%C2%BF%E2%80%A6%C3%A7%C5%93%E2%80%B9/peptides%C3%A5%C2%BF%E2%80%A6%C3%A7%C5%93%E2%80%B9/90-2.html#45) |
| 82 | 96 | 1684.79 | 1683.78 | 1683.77 | 0.0162 | 0 |  |  | Deamidated (NQ)@1; Oxidation (M)@7 | NIQADEMVEFSSGLK |
| 97 | 116 | 2133.00 | 2132.00 | 2132.02 | -0.0232 | 0 | 77.32 |  | Oxidation (M)@2; Deamidated (NQ)@5 | [GMALNLEPDNVGVVVFGNDR](../../%C3%A6%C2%A1%C5%92%C3%A9%EF%86%9D%C2%A2/%C3%A6%C2%A1%C5%92%C3%A9%C2%9D%C2%A2%C3%A6%C2%9D%C2%90%C3%A6%E2%80%93%E2%84%A2/%C3%A8%C2%B4%C2%A8%C3%A8%C2%B0%C2%B1%C3%A9%E2%80%B0%C2%B4%C3%A5%C2%AE%C5%A1%C3%A7%5C%C2%BB%E2%80%9C%C3%A6%C2%9E%C5%93/F12FTSECKJ0265_005_GS_20120821/PMF_Report%C3%A5%C2%BF%E2%80%A6%C3%A7%C5%93%E2%80%B9/peptides%C3%A5%C2%BF%E2%80%A6%C3%A7%C5%93%E2%80%B9/90-2.html#58) |
| 466 | 486 | 2408.21 | 2407.20 | 2407.16 | 0.0478 | 0 |  |  | Gln->pyro-Glu (N-term Q)@N  _term; Deamidated (NQ)@1; Carbamidomethyl (C)@18 | QGQYVPMAIEEQVAIIYCGVR |
| 99 | AAW79315.1 | 236 | 241 | 791.47 | 790.46 | 790.47 | -0.0054 | 1 |  | 141 | - | RLFVEK |
| 283 | 289 | 823.42 | 822.42 | 822.42 | -0.0060 | 0 |  |  | - | VDYALSR |
| 134 | 141 | 871.41 | 870.40 | 870.47 | -0.0643 | 0 |  |  | - | NGKPNSVR |
| 134 | 141 | 872.41 | 871.40 | 871.45 | -0.0487 | 0 |  |  | Deamidated (NQ)@1 | NGKPNSVR |
| 142 | 149 | 896.48 | 895.47 | 895.48 | -0.0064 | 0 |  |  | - | LYSIASSR |
| 165 | 174 | 1323.62 | 1322.61 | 1322.64 | -0.0303 | 1 |  |  | Carbamidomethyl (C)@6 | RATYWCPELK |
| 150 | 164 | 1690.71 | 1689.71 | 1689.73 | -0.0282 | 0 | 122.47 |  | Carbamidomethyl (C)@13 | [YGDDMTGTTTTLCVR](../../%C3%A6%C2%A1%C5%92%C3%A9%EF%86%9D%C2%A2/%C3%A6%C2%A1%C5%92%C3%A9%EF%86%9D%C2%A2%C3%A6%C2%9D%C2%90%C3%A6%E2%80%93%E2%84%A2/%C3%A8%C2%B4%C2%A8%C3%A8%C2%B0%C2%B1%C3%A9%E2%80%B0%C2%B4%C3%A5%C2%AE%C5%A1%C3%A7%5C%C2%BB%E2%80%9C%C3%A6%C2%9E%C5%93/F12FTSECKJ0265_005_GS_20120821/PMF_Report%C3%A5%C2%BF%E2%80%A6%C3%A7%C5%93%E2%80%B9/peptides%C3%A5%C2%BF%E2%80%A6%C3%A7%C5%93%E2%80%B9/99-1.html#40) |
| 150 | 164 | 1706.72 | 1705.71 | 1705.73 | -0.0210 | 0 | 106.61 |  | Oxidation (M)@5; Carbamidomethyl (C)@13 | [YGDDMTGTTTTLCVR](../../%C3%A6%C2%A1%C5%92%C3%A9%C2%9D%C2%A2/%C3%A6%C2%A1%C5%92%C3%A9%C2%9D%C2%A2%C3%A6%C2%9D%C2%90%C3%A6%E2%80%93%E2%84%A2/%C3%A8%C2%B4%C2%A8%C3%A8%C2%B0%C2%B1%C3%A9%E2%80%B0%C2%B4%C3%A5%C2%AE%C5%A1%C3%A7%5C%C2%BB%E2%80%9C%C3%A6%C5%BE%C5%93/F12FTSECKJ0265_005_GS_20120821/PMF_Report%C3%A5%C2%BF%E2%80%A6%C3%A7%C5%93%E2%80%B9/peptides%C3%A5%C2%BF%E2%80%A6%C3%A7%C5%93%E2%80%B9/99-1.html#41) |
| 34 | XP_002998682.1 | 208 | 222 | 1573.89 | 1572.88 | 1572.77 | 0.1062 | 0 |  | 222 | - | AALVYGQMNEPPGAR |
| 63 | 77 | 1678.05 | 1677.04 | 1676.92 | 0.1235 | 0 | 53.76 |  | - | [IVLEVAQHLGENTVR](../../%C3%A6%C2%A1%C5%92%C3%A9%C2%9D%C2%A2/%C3%A6%C2%A1%C5%92%C3%A9%C2%9D%C2%A2%C3%A6%C2%9D%C2%90%C3%A6%E2%80%93%E2%84%A2/%C3%A8%C2%B4%C2%A8%C3%A8%C2%B0%C2%B1%C3%A9%E2%80%B0%C2%B4%C3%A5%C2%AE%C5%A1%C3%A7%5C%C2%BB%E2%80%9C%C3%A6%C2%9E%C5%93/F12FTSECKJ0265_005_GS_20120821/PMF_Report%C3%A5%C2%BF%E2%80%A6%C3%A7%C5%93%E2%80%B9/peptides%C3%A5%C2%BF%E2%80%A6%C3%A7%C5%93%E2%80%B9/34-2.html#8) |
| 238 | 253 | 1879.03 | 1878.02 | 1877.95 | 0.0689 | 0 | 155.67 |  | - | [DVEGQDVLLFVDNIFR](../../%C3%A6%C2%A1%C5%92%C3%A9%C2%9D%C2%A2/%C3%A6%C2%A1%C5%92%C3%A9%C2%9D%C2%A2%C3%A6%C2%9D%C2%90%C3%A6%E2%80%93%E2%84%A2/%C3%A8%C2%B4%C2%A8%C3%A8%C2%B0%C2%B1%C3%A9%E2%80%B0%C2%B4%C3%A5%C2%AE%C5%A1%C3%A7%5C%C2%BB%E2%80%9C%C3%A6%C2%9E%C5%93/F12FTSECKJ0265_005_GS_20120821/PMF_Report%C3%A5%C2%BF%E2%80%A6%C3%A7%C5%93%E2%80%B9/peptides%C3%A5%C2%BF%E2%80%A6%C3%A7%C5%93%E2%80%B9/34-2.html#10) |
| 78 | BAF38479.1 | 365 | 378 | 1579.85 | 1578.84 | 1578.88 | -0.0350 | 0 | 89.51 | 347 | Gln->pyro-Glu (N-term Q)@N  _term | [QIYPPINVLPSLSR](../../%C3%A6%C2%A1%C5%92%C3%A9%C2%9D%C2%A2/%C3%A6%C2%A1%C5%92%C3%A9%C2%9D%C2%A2%C3%A6%C2%9D%C2%90%C3%A6%E2%80%93%E2%84%A2/%C3%A8%C2%B4%C2%A8%C3%A8%C2%B0%C2%B1%C3%A9%E2%80%B0%C2%B4%C3%A5%C2%AE%C5%A1%C3%A7%5C%C2%BB%E2%80%9C%C3%A6%C2%9E%C5%93/F12FTSECKJ0265_GS_20121211_489/F12FTSECKJ0265_GS_20121211_489/F12FTSECKJ0265_GS_20121211_489/PMF_Report/peptides/78-1.html#11) |
| 365 | 378 | 1596.88 | 1595.87 | 1595.90 | -0.0324 | 0 | 111.77 |  | - | [QIYPPINVLPSLSR](../../%C3%A6%C2%A1%C5%92%C3%A9%C2%9D%C2%A2/%C3%A6%C2%A1%C5%92%C3%A9%C2%9D%C2%A2%C3%A6%C2%9D%C2%90%C3%A6%E2%80%93%E2%84%A2/%C3%A8%C2%B4%C2%A8%C3%A8%C2%B0%C2%B1%C3%A9%E2%80%B0%C2%B4%C3%A5%C2%AE%C5%A1%C3%A7%5C%C2%BB%E2%80%9C%C3%A6%C2%9E%C5%93/F12FTSECKJ0265_GS_20121211_489/F12FTSECKJ0265_GS_20121211_489/F12FTSECKJ0265_GS_20121211_489/PMF_Report/peptides/78-1.html#13) |
| 450 | 463 | 1690.91 | 1689.90 | 1689.91 | -0.0049 | 0 |  |  | Deamidated (NQ)@1 | NIFQSLDLAWTLLR |
| 235 | 249 | 1715.90 | 1714.89 | 1714.93 | -0.0341 | 0 | 45.67 |  | - | [VTLFLNLANDPTIER](../../%C3%A6%C2%A1%C5%92%C3%A9%EF%86%9D%C2%A2/%C3%A6%C2%A1%C5%92%C3%A9%C2%9D%C2%A2%C3%A6%C2%9D%C2%90%C3%A6%E2%80%93%E2%84%A2/%C3%A8%C2%B4%C2%A8%C3%A8%C2%B0%C2%B1%C3%A9%E2%80%B0%C2%B4%C3%A5%C2%AE%C5%A1%C3%A7%5C%C2%BB%E2%80%9C%C3%A6%C2%9E%C5%93/F12FTSECKJ0265_GS_20121211_489/F12FTSECKJ0265_GS_20121211_489/F12FTSECKJ0265_GS_20121211_489/PMF_Report/peptides/78-1.html#23) |
| 234 | 249 | 1873.91 | 1872.90 | 1872.99 | -0.0953 | 1 |  |  | Deamidated (NQ)@7; Deamidated (NQ)@10 | RVTLFLNLANDPTIER |
| 392 | 408 | 1880.85 | 1879.84 | 1879.86 | -0.0166 | 0 |  |  | Deamidated (NQ)@7; Deamidated (NQ)@8 | DHADVSNQLYANYAIGK |
| 300 | 315 | 1928.84 | 1927.83 | 1927.87 | -0.0350 | 0 |  |  | Oxidation (M)@6 | GYPGYMYTDLATIYER |
| 391 | 408 | 2037.98 | 2036.98 | 2036.94 | 0.0312 | 1 |  |  | Deamidated (NQ)@8; Deamidated (NQ)@9; Deamidated (NQ)@13 | RDHADVSNQLYANYAIGK |
| 163 | 182 | 2178.11 | 2177.11 | 2177.14 | -0.0345 | 0 | 166.22 |  | Carbamidomethyl (C)@19 | [IPLFSAAGLPHNEIAAQICR](../../%C3%A6%C2%A1%C5%92%C3%A9%EF%86%9D%C2%A2/%C3%A6%C2%A1%C5%92%C3%A9%C2%9D%C2%A2%C3%A6%C2%9D%C2%90%C3%A6%E2%80%93%E2%84%A2/%C3%A8%C2%B4%C2%A8%C3%A8%C2%B0%C2%B1%C3%A9%E2%80%B0%C2%B4%C3%A5%C2%AE%C5%A1%C3%A7%5C%C2%BB%E2%80%9C%C3%A6%C2%9E%C5%93/F12FTSECKJ0265_GS_20121211_489/F12FTSECKJ0265_GS_20121211_489/F12FTSECKJ0265_GS_20121211_489/PMF_Report/peptides/78-1.html#51) |
| 2 | 22 | 2400.09 | 2399.09 | 2399.04 | 0.0491 | 0 |  |  | Deamidated (NQ)@3 | GVQQNNSNMEEGTLEIGMEYR |
| 1 | 22 | 2532.10 | 2531.09 | 2531.06 | 0.0314 | 0 |  |  | Deamidated (NQ)@4; Deamidated (NQ)@5 | MGVQQNNSNMEEGTLEIGMEYR |
| 1 | 22 | 2548.10 | 2547.09 | 2547.06 | 0.0335 | 0 |  |  | Oxidation (M)@1; Deamidated (NQ)@4; Deamidated (NQ)@5 | MGVQQNNSNMEEGTLEIGMEYR |
| 79 | CAD27443.1 | 182 | 188 | 755.47 | 754.46 | 754.43 | 0.0280 | 1 |  | 177 | Gln->pyro-Glu (N-term Q)@N  _term; Deamidated (NQ)@1 | QAGLVKR |
| 292 | 298 | 842.50 | 841.49 | 841.44 | 0.0526 | 1 |  |  | - | EEVPGRR |
| 381 | 389 | 937.44 | 936.43 | 936.43 | -0.0027 | 0 |  |  | Oxidation (M)@7 | SAIGEGMTR |
| 378 | 389 | 1309.61 | 1308.60 | 1308.65 | -0.0488 | 1 |  |  | Oxidation (M)@2 | LMKSAIGEGMTR |
| 364 | 377 | 1579.86 | 1578.85 | 1578.88 | -0.0222 | 0 |  |  | Gln->pyro-Glu (N-term Q)@N_term | QIYPPINVLPSLSR |
| 364 | 377 | 1596.89 | 1595.88 | 1595.90 | -0.0234 | 0 | 78.38 |  | - | [QIYPPINVLPSLSR](../../%C3%A6%C2%A1%C5%92%C3%A9%C2%9D%C2%A2/%C3%A6%C2%A1%C5%92%C3%A9%C2%9D%C2%A2%C3%A6%C2%9D%C2%90%C3%A6%E2%80%93%E2%84%A2/%C3%A8%C2%B4%C2%A8%C3%A8%C2%B0%C2%B1%C3%A9%E2%80%B0%C2%B4%C3%A5%C2%AE%C5%A1%C3%A7%5C%C2%BB%E2%80%9C%C3%A6%C2%9E%C5%93/F12FTSECKJ0265_GS_20121211_489/F12FTSECKJ0265_GS_20121211_489/F12FTSECKJ0265_GS_20121211_489/PMF_Report/peptides/79-2.html#41) |
| 449 | 462 | 1677.89 | 1676.89 | 1676.91 | -0.0272 | 0 |  |  | Deamidated (NQ)@4 | TIFQSLDLAWTLLR |
| 234 | 248 | 1715.91 | 1714.90 | 1714.93 | -0.0250 | 0 | 58.97 |  | - | [VTLFLNLANDPTIER](../../%C3%A6%C2%A1%C5%92%C3%A9%EF%86%9D%C2%A2/%C3%A6%C2%A1%C5%92%C3%A9%C2%9D%C2%A2%C3%A6%C2%9D%C2%90%C3%A6%E2%80%93%E2%84%A2/%C3%A8%C2%B4%C2%A8%C3%A8%C2%B0%C2%B1%C3%A9%E2%80%B0%C2%B4%C3%A5%C2%AE%C5%A1%C3%A7%5C%C2%BB%E2%80%9C%C3%A6%C2%9E%C5%93/F12FTSECKJ0265_GS_20121211_489/F12FTSECKJ0265_GS_20121211_489/F12FTSECKJ0265_GS_20121211_489/PMF_Report/peptides/79-2.html#50) |
| 41 | 55 | 1736.86 | 1735.85 | 1735.87 | -0.0228 | 1 |  |  | Deamidated (NQ)@2; Deamidated (NQ)@6 | YQEIVNIRLGDGTTR |
| 299 | 314 | 1928.85 | 1927.84 | 1927.87 | -0.0249 | 0 |  |  | Oxidation (M)@6 | GYPGYMYTDLATIYER |
| 390 | 407 | 2053.98 | 2052.97 | 2052.94 | 0.0346 | 1 |  |  | Deamidated (NQ)@8; Deamidated (NQ)@9; Deamidated (NQ)@13 | RDHSDVSNQLYANYAIGK |
| 162 | 181 | 2178.12 | 2177.11 | 2177.14 | -0.0312 | 0 |  |  | Carbamidomethyl (C)@19 | IPLFSAAGLPHNEIAAQICR |
| 359 | 377 | 2228.42 | 2227.42 | 2227.22 | 0.1931 | 1 |  |  | Gln->pyro-Glu (N-term Q)@N _term | QLHNRQIYPPINVLPSLSR |
| 2 | 21 | 2268.07 | 2267.07 | 2266.98 | 0.0820 | 0 |  |  | Oxidation (M)@8 | GAPNNLEMDEGNLEIGMEYR |
| 249 | 268 | 2283.06 | 2282.05 | 2282.20 | -0.1493 | 1 |  |  | Carbamidomethyl (C)@18 | IITPRIALTTAEYLAYECGK |
| 2 | 21 | 2285.09 | 2284.08 | 2283.96 | 0.1199 | 0 |  |  | Deamidated (NQ)@4; Oxidation (M)@8; Oxidation (M)@17 | GAPNNLEMDEGNLEIGMEYR |
| 1 | 21 | 2382.92 | 2381.92 | 2382.03 | -0.1119 | 0 |  |  | - | MGAPNNLEMDEGNLEIGMEYR |
| 44 | ABA55531.1 | 91 | 100 | 1028.56 | 1027.55 | 1027.57 | -0.0199 | 0 | 83.08 | 154 | - | [FPGEIAPGIK](../../%C3%A6%C2%A1%C5%92%C3%A9%EF%86%9D%C2%A2/%C3%A6%C2%A1%C5%92%C3%A9%C2%9D%C2%A2%C3%A6%C2%9D%C2%90%C3%A6%E2%80%93%E2%84%A2/%C3%A8%C2%B4%C2%A8%C3%A8%C2%B0%C2%B1%C3%A9%E2%80%B0%C2%B4%C3%A5%C2%AE%C5%A1%C3%A7%5C%C2%BB%E2%80%9C%C3%A6%C2%9E%C5%93/F12FTSECKJ0265_005_GS_20120821/PMF_Report%C3%A5%C2%BF%E2%80%A6%C3%A7%C5%93%E2%80%B9/peptides%C3%A5%C2%BF%E2%80%A6%C3%A7%C5%93%E2%80%B9/44-1.html#2) |
| 154 | 162 | 1123.50 | 1122.49 | 1122.52 | -0.0321 | 0 |  |  | Gln->pyro-Glu (N-term Q)@N  _term; Carbamidomethyl (C)@3 | QLCELQHGR |
| 154 | 162 | 1140.54 | 1139.54 | 1139.55 | -0.0130 | 0 | 62.28 |  | Carbamidomethyl (C)@3 | [QLCELQHGR](../../%C3%A6%C2%A1%C5%92%C3%A9%C2%9D%C2%A2/%C3%A6%C2%A1%C5%92%C3%A9%C2%9D%C2%A2%C3%A6%C2%9D%C2%90%C3%A6%E2%80%93%E2%84%A2/%C3%A8%C2%B4%C2%A8%C3%A8%C2%B0%C2%B1%C3%A9%E2%80%B0%C2%B4%C3%A5%C2%AE%C5%A1%C3%A7%5C%C2%BB%E2%80%9C%C3%A6%C2%9E%C5%93/F12FTSECKJ0265_005_GS_20120821/PMF_Report%C3%A5%C2%BF%E2%80%A6%C3%A7%C5%93%E2%80%B9/peptides%C3%A5%C2%BF%E2%80%A6%C3%A7%C5%93%E2%80%B9/44-1.html#13) |
| 153 | 162 | 1296.66 | 1295.65 | 1295.65 | -0.0028 | 1 |  |  | Carbamidomethyl (C)@4 | RQLCELQHGR |
| 46 | ABA55520.1 | 89 | 100 | 1236.68 | 1235.67 | 1235.66 | 0.0173 | 0 | 64.66 | 78 | - | [FPGYLSPSAGLK](../../%C3%A6%C2%A1%C5%92%C3%A9%C2%9D%C2%A2/%C3%A6%C2%A1%C5%92%C3%A9%C2%9D%C2%A2%C3%A6%C2%9D%C2%90%C3%A6%E2%80%93%E2%84%A2/%C3%A8%C2%B4%C2%A8%C3%A8%C2%B0%C2%B1%C3%A9%E2%80%B0%C2%B4%C3%A5%C2%AE%C5%A1%C3%A7%5C%C2%BB%E2%80%9C%C3%A6%C2%9E%C5%93/F12FTSECKJ0265_GS_20121211_489/F12FTSECKJ0265_GS_20121211_489/F12FTSECKJ0265_GS_20121211_489/PMF_Report/peptides/46-1.html#14) |
| 148 | 159 | 1271.61 | 1270.60 | 1270.59 | 0.0103 | 0 |  |  | - | EPGDIGGEGWVR |
| 62 | 71 | 1277.73 | 1276.72 | 1276.62 | 0.0992 | 1 |  |  | Oxidation (M)@4 | YEVMEIKHGR |
| 58 | 68 | 1491.84 | 1490.83 | 1490.69 | 0.1393 | 1 |  |  | Oxidation (M)@2 | NMYRYEVMEIK |
| 58 | 68 | 1507.84 | 1506.83 | 1506.68 | 0.1447 | 1 |  |  | Oxidation  (M) @2;  Oxidation (M)@8 | NMYRYEVMEIK |
| 47 | CBI83417.1 | 145 | 156 | 1236.68 | 1235.67 | 1235.66 | 0.0155 | 0 | 81.79 | 92 | - | [FPGYLSPSAGLK](../../%C3%A6%C2%A1%C5%92%C3%A9%EF%86%9D%C2%A2/%C3%A6%C2%A1%C5%92%C3%A9%C2%9D%C2%A2%C3%A6%C2%9D%C2%90%C3%A6%E2%80%93%E2%84%A2/%C3%A8%C2%B4%C2%A8%C3%A8%C2%B0%C2%B1%C3%A9%E2%80%B0%C2%B4%C3%A5%C2%AE%C5%A1%C3%A7%5C%C2%BB%E2%80%9C%C3%A6%C2%9E%C5%93/F12FTSECKJ0265_GS_20121211_489/F12FTSECKJ0265_GS_20121211_489/F12FTSECKJ0265_GS_20121211_489/PMF_Report/peptides/47-1.html#16) |
| 206 | 217 | 1275.72 | 1274.71 | 1274.67 | 0.0383 | 1 |  |  | - | VLTSSDPAEKTK |
| 157 | 169 | 1303.61 | 1302.61 | 1302.68 | -0.0763 | 0 | 0 |  | Deamidated (NQ)@6 | [FADVPNGLAAISK](../../%C3%A6%C2%A1%C5%92%C3%A9%C2%9D%C2%A2/%C3%A6%C2%A1%C5%92%C3%A9%C2%9D%C2%A2%C3%A6%C2%9D%C2%90%C3%A6%E2%80%93%E2%84%A2/%C3%A8%C2%B4%C2%A8%C3%A8%C2%B0%C2%B1%C3%A9%E2%80%B0%C2%B4%C3%A5%C2%AE%C5%A1%C3%A7%5C%C2%BB%E2%80%9C%C3%A6%C2%9E%C5%93/F12FTSECKJ0265_GS_20121211_489/F12FTSECKJ0265_GS_20121211_489/F12FTSECKJ0265_GS_20121211_489/PMF_Report/peptides/47-1.html#27) |
| 9 | 36 | 2580.22 | 2579.22 | 2579.28 | -0.0640 | 0 |  |  | Gln->pyro-Glu (N-term Q)@N  _term | QMAVAGLAGAALFSGATFATAGSSGNLR |
| 9 | 36 | 2596.39 | 2595.38 | 2595.28 | 0.1033 | 0 |  |  | Gln->pyro-Glu (N-term Q)@N  _term; Oxidation (M)@2 | QMAVAGLAGAALFSGATFATAGSSGNLR |
| 13 | BAJ61707.1 | 53 | 63 | 1245.61 | 1244.60 | 1244.70 | -0.0950 | 1 |  | 87 | Deamidated (NQ)@2 | LQKLVSSENVK |
| 110 | 121 | 1320.61 | 1319.60 | 1319.64 | -0.0354 | 1 |  |  | Carbamidomethyl (C)@1 | CPEVDSSKGTIK |
| 334 | 345 | 1387.74 | 1386.73 | 1386.66 | 0.0735 | 1 |  |  | - | GVFYGRLAEDFN |
| 239 | 251 | 1492.74 | 1491.73 | 1491.81 | -0.0768 | 1 |  |  | - | VPSYRTGLFLDPK |
| 191 | 207 | 1762.87 | 1761.87 | 1761.85 | 0.0132 | 0 | 76.37 |  | - | [DGIDYAATTVQLPGGER](../../%C3%A6%C2%A1%C5%92%C3%A9%C2%9D%C2%A2/%C3%A6%C2%A1%C5%92%C3%A9%C2%9D%C2%A2%C3%A6%C2%9D%C2%90%C3%A6%E2%80%93%E2%84%A2/%C3%A8%C2%B4%C2%A8%C3%A8%C2%B0%C2%B1%C3%A9%E2%80%B0%C2%B4%C3%A5%C2%AE%C5%A1%C3%A7%5C%C2%BB%E2%80%9C%C3%A6%C2%9E%C5%93/F12FTSECKJ0265_GS_20121211_489/F12FTSECKJ0265_GS_20121211_489/F12FTSECKJ0265_GS_20121211_489/PMF_Report/peptides/13-1.html#53) |
| 1 | XP_003064321.1 | 617 | 624 | 833.46 | 832.45 | 832.41 | 0.0434 | 0 |  | 94 | Carbamidomethyl (C)@2 | ECAAAIAK |
| 643 | 651 | 1105.53 | 1104.53 | 1104.60 | -0.0707 | 1 |  |  | Oxidation (M)@4 | LETMVNVRK |
| 451 | 460 | 1118.55 | 1117.54 | 1117.53 | 0.0087 | 0 | 68.79 |  | - | [TSPFPYGGHR](../../%C3%A6%C2%A1%C5%92%C3%A9%EF%86%9D%C2%A2/%C3%A6%C2%A1%C5%92%C3%A9%EF%86%9D%C2%A2%C3%A6%EF%86%9D%EF%86%90%C3%A6%E2%80%93%E2%84%A2/%C3%A8%C2%B4%C2%A8%C3%A8%C2%B0%C2%B1%C3%A9%E2%80%B0%C2%B4%C3%A5%C2%AE%C5%A1%C3%A7%5C%C2%BB%E2%80%9C%C3%A6%C2%9E%C5%93/F12FTSECKJ0265_005_GS_20120821/PMF_Report%C3%A5%C2%BF%E2%80%A6%C3%A7%C5%93%E2%80%B9/peptides%C3%A5%C2%BF%E2%80%A6%C3%A7%C5%93%E2%80%B9/1-2.html#17) |
| 163 | 172 | 1179.61 | 1178.61 | 1178.64 | -0.0345 | 1 |  |  | - | ALDEKTPLHR |
| 217 | 229 | 1481.78 | 1480.77 | 1480.77 | 0.0036 | 1 |  |  | Oxidation (M)@1 | MDLQFTGRTVLGK |
| 529 | 546 | 1847.92 | 1846.91 | 1846.91 | 0.0054 | 1 |  |  | Deamidated (NQ)@17 | ADAMGVTRIDSGVEAINK |
| 109 | 126 | 1939.02 | 1938.01 | 1937.86 | 0.1556 | 0 | 0 |  | Deamidated (NQ)@14 | [HILFGETDGSSYPNGGMR](../../%C3%A6%C2%A1%C5%92%C3%A9%C2%9D%C2%A2/%C3%A6%C2%A1%C5%92%C3%A9%C2%9D%C2%A2%C3%A6%C2%9D%C2%90%C3%A6%E2%80%93%E2%84%A2/%C3%A8%C2%B4%C2%A8%C3%A8%C2%B0%C2%B1%C3%A9%E2%80%B0%C2%B4%C3%A5%C2%AE%C5%A1%C3%A7%5C%C2%BB%E2%80%9C%C3%A6%C2%9E%C5%93/F12FTSECKJ0265_005_GS_20120821/PMF_Report%C3%A5%C2%BF%E2%80%A6%C3%A7%C5%93%E2%80%B9/peptides%C3%A5%C2%BF%E2%80%A6%C3%A7%C5%93%E2%80%B9/1-2.html#48) |
| 65 | 81 | 2088.05 | 2087.04 | 2086.90 | 0.1370 | 0 |  |  | Deamidated  (NQ)@4;  Carbamidomethyl (C)@6;  Deamidated (NQ)@10; Oxidation (M)@12 | GVTNFCHWFQPMAATFR |
| 27 | 45 | 2104.05 | 2103.04 | 2103.09 | -0.0510 | 1 |  |  | Deamidated (NQ)@5 | FLKEQGLSAAVLDDPSWVK |
| 1 | 20 | 2120.05 | 2119.04 | 2118.97 | 0.0714 | 0 |  |  | Deamidated (NQ)@3 | MGQNVDAPHLSAGFGEDVFK |
| 187 | 210 | 2705.20 | 2704.19 | 2704.33 | -0.1395 | 0 |  |  | Oxidation (M)@2;  Deamidated (NQ)@11 | HMGFEVAGAVNNIGLEQELFFIPR |
| 2 | XP_003064321.1 | 617 | 624 | 833.44 | 832.43 | 832.41 | 0.0172 | 0 |  | 97 | Carbamidomethyl (C)@2 | ECAAAIAK |
| 537 | 546 | 1045.53 | 1044.52 | 1044.55 | -0.0213 | 0 |  |  | - | IDSGVEAINK |
| 56 | 64 | 1077.55 | 1076.55 | 1076.57 | -0.0212 | 0 |  |  | Carbamidomethyl (C)@6 | AVLTWCLSK |
| 643 | 651 | 1105.50 | 1104.49 | 1104.60 | -0.1018 | 1 |  |  | Oxidation (M)@4 | LETMVNVRK |
| 451 | 460 | 1118.52 | 1117.51 | 1117.53 | -0.0220 | 0 | 67.56 |  | - | [TSPFPYGGHR](../../%C3%A6%C2%A1%C5%92%C3%A9%C2%9D%C2%A2/%C3%A6%C2%A1%C5%92%C3%A9%C2%9D%C2%A2%C3%A6%C2%9D%C2%90%C3%A6%E2%80%93%E2%84%A2/%C3%A8%C2%B4%C2%A8%C3%A8%C2%B0%C2%B1%C3%A9%E2%80%B0%C2%B4%C3%A5%C2%AE%C5%A1%C3%A7%5C%C2%BB%E2%80%9C%C3%A6%C2%9E%C5%93/F12FTSECKJ0265_005_GS_20120821/PMF_Report%C3%A5%C2%BF%E2%80%A6%C3%A7%C5%93%E2%80%B9/peptides%C3%A5%C2%BF%E2%80%A6%C3%A7%C5%93%E2%80%B9/2-1.html#19) |
| 216 | 224 | 1140.49 | 1139.49 | 1139.54 | -0.0539 | 1 |  |  | Oxidation (M)@2;  Deamidated (NQ)@5 | RMDLQFTGR |
| 217 | 229 | 1481.74 | 1480.73 | 1480.77 | -0.0397 | 1 |  |  | Oxidation (M)@1 | MDLQFTGRTVLGK |
| 529 | 546 | 1847.89 | 1846.88 | 1846.91 | -0.0281 | 1 |  |  | Deamidated (NQ)@17 | ADAMGVTRIDSGVEAINK |
| 109 | 126 | 1938.98 | 1937.97 | 1937.86 | 0.1143 | 0 | 0 |  | Deamidated (NQ)@14 | [HILFGETDGSSYPNGGMR](../../%C3%A6%C2%A1%C5%92%C3%A9%C2%9D%C2%A2/%C3%A6%C2%A1%C5%92%C3%A9%C2%9D%C2%A2%C3%A6%C2%9D%C2%90%C3%A6%E2%80%93%E2%84%A2/%C3%A8%C2%B4%C2%A8%C3%A8%C2%B0%C2%B1%C3%A9%E2%80%B0%C2%B4%C3%A5%C2%AE%C5%A1%C3%A7%5C%C2%BB%E2%80%9C%C3%A6%C2%9E%C5%93/F12FTSECKJ0265_005_GS_20120821/PMF_Report%C3%A5%C2%BF%E2%80%A6%C3%A7%C5%93%E2%80%B9/peptides%C3%A5%C2%BF%E2%80%A6%C3%A7%C5%93%E2%80%B9/2-1.html#40) |
| 27 | 45 | 2104.01 | 2103.00 | 2103.09 | -0.0840 | 1 |  |  | Deamidated (NQ)@5 | FLKEQGLSAAVLDDPSWVK |
| 16* | YP_002834016.1 | 415 | 420 | 755.37 | 754.36 | 754.36 | 0.0018 | 1 |  | 88 | - | SYSKDR |
| 67 | 73 | 781.39 | 780.38 | 780.36 | 0.0167 | 0 |  |  | Oxidation (M)@1;  Oxidation (M)@5 | MAIGMAR |
| 201 | 208 | 842.50 | 841.49 | 841.56 | -0.0741 | 0 |  |  | - | LVGLITVK |
| 266 | 272 | 849.43 | 848.42 | 848.44 | -0.0234 | 0 |  |  | Oxidation (M)@4 | VLEMVSR |
| 421 | 427 | 870.42 | 869.41 | 869.43 | -0.0145 | 0 |  |  | - | YFQADVK |
| 74 | 82 | 920.45 | 919.45 | 919.49 | -0.0424 | 0 |  |  | Gln->pyro-Glu (N-term Q)@N  _term;  Deamidated (NQ)@1 | QGGIGVLHR |
| 273 | 280 | 964.46 | 963.45 | 963.48 | -0.0258 | 1 |  |  | - | VQKDFGDR |
| 397 | 407 | 1114.51 | 1113.50 | 1113.44 | 0.0619 | 0 | 0 |  | Oxidation (M)@2;  Oxidation (M)@5 | [GMGSMGAMQGR](../../%C3%A6%C2%A1%C5%92%C3%A9%C2%9D%C2%A2/%C3%A6%C2%A1%C5%92%C3%A9%C2%9D%C2%A2%C3%A6%C2%9D%C2%90%C3%A6%E2%80%93%E2%84%A2/%C3%A8%C2%B4%C2%A8%C3%A8%C2%B0%C2%B1%C3%A9%E2%80%B0%C2%B4%C3%A5%C2%AE%C5%A1%C3%A7%5C%C2%BB%E2%80%9C%C3%A6%C2%9E%C5%93/F12FTSECKJ0265_005_GS_20120821/PMF_Report%C3%A5%C2%BF%E2%80%A6%C3%A7%C5%93%E2%80%B9/peptides%C3%A5%C2%BF%E2%80%A6%C3%A7%C5%93%E2%80%B9/L3-1.html#21) |
| 419 | 427 | 1141.58 | 1140.57 | 1140.56 | 0.0129 | 1 |  |  | - | DRYFQADVK |
| 137 | 147 | 1201.63 | 1200.62 | 1200.59 | 0.0317 | 0 | 0 |  | Gln->pyro-Glu (N-term Q)@N  _term;  Carbamidomethyl (C)@8 | [QGTLVGICTNR](../../%C3%A6%C2%A1%C5%92%C3%A9%C2%9D%C2%A2/%C3%A6%C2%A1%C5%92%C3%A9%C2%9D%C2%A2%C3%A6%C2%9D%C2%90%C3%A6%E2%80%93%E2%84%A2/%C3%A8%C2%B4%C2%A8%C3%A8%C2%B0%C2%B1%C3%A9%E2%80%B0%C2%B4%C3%A5%C2%AE%C5%A1%C3%A7%5C%C2%BB%E2%80%9C%C3%A6%C2%9E%C5%93/F12FTSECKJ0265_005_GS_20120821/PMF_Report%C3%A5%C2%BF%E2%80%A6%C3%A7%C5%93%E2%80%B9/peptides%C3%A5%C2%BF%E2%80%A6%C3%A7%C5%93%E2%80%B9/L3-1.html#29) |
| 395 | 407 | 1450.66 | 1449.65 | 1449.58 | 0.0740 | 1 | 3.77 |  | Oxidation (M)@4;  Oxidation (M)@7;  Oxidation (M)@10;  Deamidated (NQ)@11 | [YRGMGSMGAMQGR](../../%C3%A6%C2%A1%C5%92%C3%A9%C2%9D%C2%A2/%C3%A6%C2%A1%C5%92%C3%A9%C2%9D%C2%A2%C3%A6%C2%9D%C2%90%C3%A6%E2%80%93%E2%84%A2/%C3%A8%C2%B4%C2%A8%C3%A8%C2%B0%C2%B1%C3%A9%E2%80%B0%C2%B4%C3%A5%C2%AE%C5%A1%C3%A7%5C%C2%BB%E2%80%9C%C3%A6%C2%9E%C5%93/F12FTSECKJ0265_005_GS_20120821/PMF_Report%C3%A5%C2%BF%E2%80%A6%C3%A7%C5%93%E2%80%B9/peptides%C3%A5%C2%BF%E2%80%A6%C3%A7%C5%93%E2%80%B9/L3-1.html#34) |
| 460 | 474 | 1529.70 | 1528.70 | 1528.71 | -0.0123 | 0 |  |  | Oxidation (M)@3;  Deamidated (NQ)@10 | ASMGYTGSANLAELK |
| 281 | 295 | 1556.75 | 1555.75 | 1555.83 | -0.0850 | 1 |  |  | Deamidated (NQ)@7 | IDVIGGNLATREAAR |
| 397 | 413 | 1670.73 | 1669.72 | 1669.72 | 0.0006 | 1 |  |  | Oxidation (M)@2;  Deamidated (NQ)@9 | GMGSMGAMQGRGLSGEK |
| 460 | 476 | 1741.78 | 1740.78 | 1740.87 | -0.0962 | 1 | 0 |  | - | [ASMGYTGSANLAELKTK](../../%C3%A6%C2%A1%C5%92%C3%A9%EF%86%9D%C2%A2/%C3%A6%C2%A1%C5%92%C3%A9%C2%9D%C2%A2%C3%A6%C2%9D%C2%90%C3%A6%E2%80%93%E2%84%A2/%C3%A8%C2%B4%C2%A8%C3%A8%C2%B0%C2%B1%C3%A9%E2%80%B0%C2%B4%C3%A5%C2%AE%C5%A1%C3%A7%5C%C2%BB%E2%80%9C%C3%A6%C2%9E%C5%93/F12FTSECKJ0265_005_GS_20120821/PMF_Report%C3%A5%C2%BF%E2%80%A6%C3%A7%C5%93%E2%80%B9/peptides%C3%A5%C2%BF%E2%80%A6%C3%A7%C5%93%E2%80%B9/L3-1.html#40) |
| 74 | 97 | 2590.25 | 2589.24 | 2589.29 | -0.0526 | 1 |  |  | Gln->pyro-Glu (N-term Q)@N  _term;  Deamidated (NQ)@1;  Deamidated (NQ)@10;  Deamidated (NQ)@16;  Deamidated (NQ)@18 | QGGIGVLHRNLSAEEQAQQVEIVK |
| 21* | BAB47124.1 | 101 | 109 | 1144.59 | 1143.58 | 1143.62 | -0.0343 | 1 |  | 87 | Deamidated (NQ)@1 | NAWRILWGK |
| 348 | 358 | 1147.62 | 1146.61 | 1146.64 | -0.0314 | 0 | 1.34 |  | Deamidated (NQ)@4 | [GLAQVGIGYLR](../../%C3%A6%C2%A1%C5%92%C3%A9%C2%9D%C2%A2/%C3%A6%C2%A1%C5%92%C3%A9%C2%9D%C2%A2%C3%A6%C2%9D%C2%90%C3%A6%E2%80%93%E2%84%A2/%C3%A8%C2%B4%C2%A8%C3%A8%C2%B0%C2%B1%C3%A9%E2%80%B0%C2%B4%C3%A5%C2%AE%C5%A1%C3%A7%5C%C2%BB%E2%80%9C%C3%A6%C5%BE%C5%93/F12FTSECKJ0265_GS_20121211_489/F12FTSECKJ0265_GS_20121211_489/F12FTSECKJ0265_GS_20121211_489/PMF_Report/peptides/21-1.html#17) |
| 377 | 386 | 1219.59 | 1218.59 | 1218.61 | -0.0213 | 0 |  |  | - | TLEAEMALWR |
| 453 | 463 | 1276.72 | 1275.72 | 1275.68 | 0.0379 | 1 |  |  | Deamidated (NQ)@1 | QSSLELRLSSR |
| 2 | 14 | 1358.65 | 1357.64 | 1357.76 | -0.1146 | 0 |  |  | - | VLVSSNANNLLSK |
| 432 | 443 | 1421.69 | 1420.68 | 1420.76 | -0.0751 | 1 |  |  | Deamidated (NQ)@1 | NFVLQNKEVTTK |
| 219 | 231 | 1448.78 | 1447.77 | 1447.71 | 0.0664 | 0 |  |  | Gln->pyro-Glu (N-term Q)@N  _term;  Deamidated (NQ)@1;  Deamidated (NQ)@11;  Deamidated (NQ)@12 | QTLETAVAFINQK |
| 168 | 180 | 1479.74 | 1478.73 | 1478.71 | 0.0267 | 0 |  |  | Carbamidomethyl (C)@6;  Deamidated (NQ)@7;  Deamidated (NQ)@10 | IVPVVCNSSNNFK |
| 1 | 14 | 1492.79 | 1491.78 | 1491.75 | 0.0327 | 0 |  |  | Deamidated (NQ)@7;  Deamidated (NQ)@9;  Deamidated (NQ)@10 | MVLVSSNANNLLSK |
| 2 | 17 | 1673.83 | 1672.83 | 1672.92 | -0.0982 | 1 | 0 |  | Deamidated (NQ)@6;  Deamidated (NQ)@8;  Deamidated (NQ)@9 | [VLVSSNANNLLSKLAK](../../%C3%A6%C2%A1%C5%92%C3%A9%C2%9D%C2%A2/%C3%A6%C2%A1%C5%92%C3%A9%C2%9D%C2%A2%C3%A6%C2%9D%C2%90%C3%A6%E2%80%93%E2%84%A2/%C3%A8%C2%B4%C2%A8%C3%A8%C2%B0%C2%B1%C3%A9%E2%80%B0%C2%B4%C3%A5%C2%AE%C5%A1%C3%A7%5C%C2%BB%E2%80%9C%C3%A6%C2%9E%C5%93/F12FTSECKJ0265_GS_20121211_489/F12FTSECKJ0265_GS_20121211_489/F12FTSECKJ0265_GS_20121211_489/PMF_Report/peptides/21-1.html#53) |
| 1 | 17 | 1804.91 | 1803.90 | 1803.97 | -0.0620 | 1 |  |  | Deamidated (NQ)@7;  Deamidated (NQ)@9;  Deamidated (NQ)@10 | MVLVSSNANNLLSKLAK |
| 184 | 199 | 1825.95 | 1824.94 | 1824.86 | 0.0774 | 1 |  |  | Deamidated (NQ)@11;  Deamidated (NQ)@14 | EALETAYEKAQESNIK |
| 202 | 218 | 1828.89 | 1827.88 | 1827.90 | -0.0240 | 0 |  |  | Deamidated (NQ)@6;  Deamidated (NQ)@9;  Carbamidomethyl (C)@15 | GLLITNPSNPLGTVCDR |
| 164 | 180 | 1865.07 | 1864.06 | 1863.94 | 0.1246 | 1 |  |  | Carbamidomethyl (C)@10;  Deamidated (NQ)@11;  Deamidated (NQ)@14 | TGVKIVPVVCNSSNNFK |
| 371 | 386 | 1940.94 | 1939.93 | 1940.02 | -0.0878 | 1 |  |  | - | HLLTEKTLEAEMALWR |
| 371 | 386 | 1957.00 | 1956.00 | 1956.01 | -0.0162 | 1 |  |  | Oxidation (M)@12 | HLLTEKTLEAEMALWR |
| 377 | 393 | 2002.05 | 2001.05 | 2001.06 | -0.0153 | 1 |  |  | Deamidated (NQ)@14 | TLEAEMALWRVIINDVK |
| 413 | 429 | 2050.98 | 2049.98 | 2049.83 | 0.1445 | 0 |  |  | Carbamidomethyl (C)@2;  Deamidated (NQ)@5;  Oxidation (M)@6;  Deamidated (NQ)@9;  Oxidation (M)@11 | VCFANMDDNTMEISITR |
| 394 | 412 | 2062.16 | 2061.16 | 2061.05 | 0.1071 | 0 |  |  | Carbamidomethyl (C)@3 | AECVAGVVFPLLGSPGWFR |
| 200 | 218 | 2067.95 | 2066.94 | 2067.11 | -0.1705 | 1 |  |  | Carbamidomethyl (C)@17 | IKGLLITNPSNPLGTVCDR |
| 464 | 482 | 2221.10 | 2220.10 | 2220.10 | -0.0072 | 1 |  |  | Oxidation (M)@6;  Deamidated (NQ)@14 | RLEDIMSPHSPLPQSPMLR |
| 47 | 68 | 2572.32 | 2571.32 | 2571.21 | 0.1086 | 0 |  |  | Deamidated (NQ)@4;  Oxidation (M)@5;  Deamidated (NQ)@10;  Deamidated (NQ)@11 | GVIQMGLAENQLSFEFVEDWIK |
| 11 | AAW79325.1 | 48 | 54 | 788.42 | 787.41 | 787.42 | -0.0053 | 1 |  | 130 | - | ADLEGKR |
| 69 | 75 | 821.40 | 820.39 | 820.39 | -0.0033 | 0 |  |  | - | TITDDTR |
| 110 | 118 | 943.53 | 942.52 | 942.53 | -0.0108 | 0 |  |  | - | FSLGPVAPR |
| 191 | 200 | 986.49 | 985.48 | 985.48 | -0.0038 | 0 |  |  | - | AHGSTEGVTK |
| 227 | 237 | 1102.62 | 1101.61 | 1101.63 | -0.0197 | 0 |  |  | - | RPFAAIVGGSK |
| 242 | 251 | 1134.59 | 1133.58 | 1133.60 | -0.0196 | 0 |  |  | Oxidation (M)@8 | IGVIESLMEK |
| 93 | 103 | 1206.71 | 1205.70 | 1205.72 | -0.0212 | 0 | 7.24 |  | - | [VLLSSHLGRPK](../../%C3%A6%C2%A1%C5%92%C3%A9%C2%9D%C2%A2/%C3%A6%C2%A1%C5%92%C3%A9%C2%9D%C2%A2%C3%A6%C2%9D%C2%90%C3%A6%E2%80%93%E2%84%A2/%C3%A8%C2%B4%C2%A8%C3%A8%C2%B0%C2%B1%C3%A9%E2%80%B0%C2%B4%C3%A5%C2%AE%C5%A1%C3%A7%5C%C2%BB%E2%80%9C%C3%A6%C2%9E%C5%93/F12FTSECKJ0265_005_GS_20120821/PMF_Report%C3%A5%C2%BF%E2%80%A6%C3%A7%C5%93%E2%80%B9/peptides%C3%A5%C2%BF%E2%80%A6%C3%A7%C5%93%E2%80%B9/11-1.html#26) |
| 173 | 190 | 1920.89 | 1919.88 | 1919.89 | -0.0144 | 0 | 12.69 |  | - | [LAANADMFVNDAFGTAHR](../../%C3%A6%C2%A1%C5%92%C3%A9%C2%9D%C2%A2/%C3%A6%C2%A1%C5%92%C3%A9%C2%9D%C2%A2%C3%A6%C2%9D%C2%90%C3%A6%E2%80%93%E2%84%A2/%C3%A8%C2%B4%C2%A8%C3%A8%C2%B0%C2%B1%C3%A9%E2%80%B0%C2%B4%C3%A5%C2%AE%C5%A1%C3%A7%5C%C2%BB%E2%80%9C%C3%A6%C2%9E%C5%93/F12FTSECKJ0265_005_GS_20120821/PMF_Report%C3%A5%C2%BF%E2%80%A6%C3%A7%C5%93%E2%80%B9/peptides%C3%A5%C2%BF%E2%80%A6%C3%A7%C5%93%E2%80%B9/11-1.html#32) |
| 173 | 190 | 1936.89 | 1935.88 | 1935.89 | -0.0053 | 0 | 72.12 |  | Oxidation (M)@7 | [LAANADMFVNDAFGTAHR](../../%C3%A6%C2%A1%C5%92%C3%A9%C2%9D%C2%A2/%C3%A6%C2%A1%C5%92%C3%A9%C2%9D%C2%A2%C3%A6%C2%9D%C2%90%C3%A6%E2%80%93%E2%84%A2/%C3%A8%C2%B4%C2%A8%C3%A8%C2%B0%C2%B1%C3%A9%E2%80%B0%C2%B4%C3%A5%C2%AE%C5%A1%C3%A7%5C%C2%BB%E2%80%9C%C3%A6%C2%9E%C5%93/F12FTSECKJ0265_005_GS_20120821/PMF_Report%C3%A5%C2%BF%E2%80%A6%C3%A7%C5%93%E2%80%B9/peptides%C3%A5%C2%BF%E2%80%A6%C3%A7%C5%93%E2%80%B9/11-1.html#34) |
| 214 | 237 | 2505.27 | 2504.26 | 2504.29 | -0.0324 | 1 |  |  | - | ELDYLDGAVSDPKRPFAAIVGGSK |
| 35 | AAW79326.1 | 7 | 16 | 1073.57 | 1072.56 | 1072.55 | 0.0066 | 0 |  | 123 | - | DPSVITNTAR |
| 6 | 16 | 1201.63 | 1200.62 | 1200.65 | -0.0247 | 1 |  |  | - | KDPSVITNTAR |
| 197 | 206 | 1216.61 | 1215.60 | 1215.69 | -0.0913 | 1 |  |  | Deamidated (NQ)@2 | IQLIKNMLDK |
| 133 | 147 | 1607.78 | 1606.77 | 1606.77 | -0.0032 | 0 | 110.96 |  | - | [LGDVYVSDAFGTAHR](../../%C3%A6%C2%A1%C5%92%C3%A9%EF%86%9D%C2%A2/%C3%A6%C2%A1%C5%92%C3%A9%C2%9D%C2%A2%C3%A6%C2%9D%C2%90%C3%A6%E2%80%93%E2%84%A2/%C3%A8%C2%B4%C2%A8%C3%A8%C2%B0%C2%B1%C3%A9%E2%80%B0%C2%B4%C3%A5%C2%AE%C5%A1%C3%A7%5C%C2%BB%E2%80%9C%C3%A6%C2%9E%C5%93/F12FTSECKJ0265_GS_20121211_489/F12FTSECKJ0265_GS_20121211_489/F12FTSECKJ0265_GS_20121211_489/PMF_Report/peptides/35-1.html#35) |
| 223 | 240 | 1975.94 | 1974.93 | 1974.94 | -0.0055 | 0 |  |  | Oxidation (M)@5 | VLHNMPIGSSLYDEEGAK |
| 428 | 448 | 2435.21 | 2434.21 | 2434.10 | 0.1034 | 1 |  |  | Oxidation (M)@6;  Carbamidomethyl (C)@9;  Carbamidomethyl (C)@11 | GLWAWMLGCGCAPAVEGWLRE |
| 28 | AAW79327.1 | 23 | 31 | 1061.54 | 1060.53 | 1060.52 | 0.0112 | 0 |  | 137 | - | VDFNVPQDK |
| 174 | 186 | 1365.66 | 1364.65 | 1364.64 | 0.0128 | 0 |  |  | - | AHSSMLGEGFTTK |
| 236 | 248 | 1433.73 | 1432.72 | 1432.71 | 0.0153 | 0 |  |  | Oxidation (M)@1;  Oxidation (M)@7 | MIIGGGMAYTFLK |
| 2 | 15 | 1491.72 | 1490.71 | 1490.82 | -0.1097 | 1 |  |  | Deamidated (NQ)@7 | SLSSKLQLSSVDVK |
| 159 | 173 | 1607.80 | 1606.79 | 1606.77 | 0.0180 | 0 | 119.5 |  | - | [LGDVYVSDAFGTAHR](../../%C3%A6%C2%A1%C5%92%C3%A9%C2%9D%C2%A2/%C3%A6%C2%A1%C5%92%C3%A9%C2%9D%C2%A2%C3%A6%C2%9D%C2%90%C3%A6%E2%80%93%E2%84%A2/%C3%A8%C2%B4%C2%A8%C3%A8%C2%B0%C2%B1%C3%A9%E2%80%B0%C2%B4%C3%A5%C2%AE%C5%A1%C3%A7%5C%C2%BB%E2%80%9C%C3%A6%C2%9E%C5%93/F12FTSECKJ0265_GS_20121211_489/F12FTSECKJ0265_GS_20121211_489/F12FTSECKJ0265_GS_20121211_489/PMF_Report/peptides/28-1.html#41) |
| 1 | 15 | 1638.86 | 1637.85 | 1637.85 | -0.0015 | 1 |  |  | Oxidation (M)@1;  Deamidated (NQ)@8 | MSLSSKLQLSSVDVK |
| 126 | 141 | 1791.75 | 1790.74 | 1790.83 | -0.0935 | 1 |  |  | - | FHIEEEGKATTESGEK |
| 43 | 59 | 1809.96 | 1808.95 | 1808.95 | 0.0066 | 1 |  |  | Carbamidomethyl (C)@11 | IEGALPTIKHCLDSGAK |
| 7 | AAR97551.1 | 226 | 238 | 1376.67 | 1375.66 | 1375.77 | -0.1119 | 1 | 0 | 156 | - | [IGLDVAASEFKVK](../../%C3%A6%C2%A1%C5%92%C3%A9%EF%86%9D%C2%A2/%C3%A6%C2%A1%C5%92%C3%A9%EF%86%9D%C2%A2%C3%A6%C2%9D%C2%90%C3%A6%E2%80%93%E2%84%A2/%C3%A8%C2%B4%C2%A8%C3%A8%C2%B0%C2%B1%C3%A9%E2%80%B0%C2%B4%C3%A5%C2%AE%C5%A1%C3%A7%5C%C2%BB%E2%80%9C%C3%A6%C2%9E%C5%93/F12FTSECKJ0265_005_GS_20120821/PMF_Report%C3%A5%C2%BF%E2%80%A6%C3%A7%C5%93%E2%80%B9/peptides%C3%A5%C2%BF%E2%80%A6%C3%A7%C5%93%E2%80%B9/7-1.html#17) |
| 19 | 36 | 1764.88 | 1763.87 | 1763.87 | 0.0022 | 0 | 145.19 |  | - | [ASVPSGASTGAYEAVELR](../../%C3%A6%C2%A1%C5%92%C3%A9%C2%9D%C2%A2/%C3%A6%C2%A1%C5%92%C3%A9%C2%9D%C2%A2%C3%A6%C2%9D%C2%90%C3%A6%E2%80%93%E2%84%A2/%C3%A8%C2%B4%C2%A8%C3%A8%C2%B0%C2%B1%C3%A9%E2%80%B0%C2%B4%C3%A5%C2%AE%C5%A1%C3%A7%5C%C2%BB%E2%80%9C%C3%A6%C2%9E%C5%93/F12FTSECKJ0265_005_GS_20120821/PMF_Report%C3%A5%C2%BF%E2%80%A6%C3%A7%C5%93%E2%80%B9/peptides%C3%A5%C2%BF%E2%80%A6%C3%A7%C5%93%E2%80%B9/7-1.html#21) |
| 1 | 18 | 1878.95 | 1877.94 | 1877.90 | 0.0393 | 0 |  |  | Deamidated (NQ)@3 | SGNPTVEVEVTTADGVFR |
| 77 | CAB75428.1 | 11 | 16 | 748.35 | 747.35 | 747.36 | -0.0078 | 0 |  | 161 | Gln->pyro-Glu (N-term Q)@N  _term | QIFDSR |
| 11 | 16 | 765.38 | 764.38 | 764.38 | -0.0050 | 0 |  |  | - | QIFDSR |
| 416 | 421 | 806.44 | 805.44 | 805.44 | -0.0080 | 0 |  |  | - | YNQLLR |
| 372 | 381 | 1101.52 | 1100.51 | 1100.52 | -0.0088 | 0 |  |  | - | AGWGVMTSHR |
| 372 | 381 | 1117.51 | 1116.51 | 1116.51 | -0.0073 | 0 |  |  | Oxidation (M)@6 | AGWGVMTSHR |
| 126 | 137 | 1231.64 | 1230.63 | 1230.73 | -0.1045 | 1 |  |  | - | AGASALKIPLYK |
| 53 | 65 | 1282.53 | 1281.52 | 1281.62 | -0.0972 | 1 |  |  | - | DGGSDYLGKGVSK |
| 353 | 367 | 1573.81 | 1572.81 | 1572.84 | -0.0303 | 0 |  |  | - | VNQIGSVTESIEAVK |
| 66 | 81 | 1610.81 | 1609.81 | 1609.89 | -0.0864 | 0 |  |  | Deamidated (NQ)@4;  Deamidated (NQ)@6 | AVDNVNTIIAPALIGK |
| 263 | 277 | 1773.85 | 1772.84 | 1772.77 | 0.0641 | 1 |  |  | Deamidated (NQ)@4;  Deamidated (NQ)@9;  Deamidated (NQ)@10 | YVLNFKEDNNDGSQK |
| 35 | 52 | 1804.94 | 1803.93 | 1803.94 | -0.0083 | 0 | 132.88 |  | - | [AAVPSGASTGIYEALELR](../../%C3%A6%C2%A1%C5%92%C3%A9%EF%86%9D%C2%A2/%C3%A6%C2%A1%C5%92%C3%A9%C2%9D%C2%A2%C3%A6%C2%9D%C2%90%C3%A6%E2%80%93%E2%84%A2/%C3%A8%C2%B4%C2%A8%C3%A8%C2%B0%C2%B1%C3%A9%E2%80%B0%C2%B4%C3%A5%C2%AE%C5%A1%C3%A7%5C%C2%BB%E2%80%9C%C3%A6%C2%9E%C5%93/F12FTSECKJ0265_GS_20121211_489/F12FTSECKJ0265_GS_20121211_489/F12FTSECKJ0265_GS_20121211_489/PMF_Report/peptides/77-2.html#65) |
| 245 | 261 | 1831.92 | 1830.92 | 1830.87 | 0.0445 | 0 |  |  | - | VVIGMDVAASEFYTSDK |
| 245 | 261 | 1847.91 | 1846.91 | 1846.87 | 0.0416 | 0 |  |  | Oxidation (M)@5 | VVIGMDVAASEFYTSDK |
| 168 | 184 | 1883.87 | 1882.87 | 1882.96 | -0.0912 | 0 |  |  | Deamidated (NQ)@4 | LAMQEFMILPVGASTFK |
| 68 | YP_562918.1 | 506 | 512 | 745.36 | 744.35 | 744.41 | -0.0634 | 0 |  | 94 | - | VNIANSK |
| 230 | 237 | 834.40 | 833.39 | 833.41 | -0.0231 | 0 |  |  | - | GAVTTEEK |
| 159 | 167 | 864.41 | 863.40 | 863.40 | 0.0034 | 1 |  |  | Deamidated (NQ)@6 | GGGSENKSK |
| 418 | 426 | 907.39 | 906.38 | 906.47 | -0.0844 | 0 |  |  | - | TGLMGMIGK |
| 230 | 239 | 1103.55 | 1102.54 | 1102.60 | -0.0602 | 1 | 42.26 |  | - | [GAVTTEEKLR](../../%C3%A6%C2%A1%C5%92%C3%A9%EF%86%9D%C2%A2/%C3%A6%C2%A1%C5%92%C3%A9%C2%9D%C2%A2%C3%A6%C2%9D%C2%90%C3%A6%E2%80%93%E2%84%A2/%C3%A8%C2%B4%C2%A8%C3%A8%C2%B0%C2%B1%C3%A9%E2%80%B0%C2%B4%C3%A5%C2%AE%C5%A1%C3%A7%5C%C2%BB%E2%80%9C%C3%A6%C2%9E%C5%93/F12FTSECKJ0265_005_GS_20120821/PMF_Report%C3%A5%C2%BF%E2%80%A6%C3%A7%C5%93%E2%80%B9/peptides%C3%A5%C2%BF%E2%80%A6%C3%A7%C5%93%E2%80%B9/68-1.html#16) |
| 120 | 131 | 1185.61 | 1184.60 | 1184.65 | -0.0475 | 1 |  |  | - | ASIVADPAGTRK |
| 427 | 438 | 1331.69 | 1330.68 | 1330.67 | 0.0104 | 1 | 0 |  | Deamidated (NQ)@6 | [SERGPQTVESIK](../../%C3%A6%C2%A1%C5%92%C3%A9%C2%9D%C2%A2/%C3%A6%C2%A1%C5%92%C3%A9%C2%9D%C2%A2%C3%A6%C2%9D%C2%90%C3%A6%E2%80%93%E2%84%A2/%C3%A8%C2%B4%C2%A8%C3%A8%C2%B0%C2%B1%C3%A9%E2%80%B0%C2%B4%C3%A5%C2%AE%C5%A1%C3%A7%5C%C2%BB%E2%80%9C%C3%A6%C2%9E%C5%93/F12FTSECKJ0265_005_GS_20120821/PMF_Report%C3%A5%C2%BF%E2%80%A6%C3%A7%C5%93%E2%80%B9/peptides%C3%A5%C2%BF%E2%80%A6%C3%A7%C5%93%E2%80%B9/68-1.html#24) |
| 95 | 108 | 1655.79 | 1654.78 | 1654.72 | 0.0668 | 0 | 20 |  | Oxidation (M)@3;  Oxidation (M)@8 | [TDMTIQEMVDEGVR](../../%C3%A6%C2%A1%C5%92%C3%A9%C2%9D%C2%A2/%C3%A6%C2%A1%C5%92%C3%A9%C2%9D%C2%A2%C3%A6%C2%9D%C2%90%C3%A6%E2%80%93%E2%84%A2/%C3%A8%C2%B4%C2%A8%C3%A8%C2%B0%C2%B1%C3%A9%E2%80%B0%C2%B4%C3%A5%C2%AE%C5%A1%C3%A7%5C%C2%BB%E2%80%9C%C3%A6%C2%9E%C5%93/F12FTSECKJ0265_005_GS_20120821/PMF_Report%C3%A5%C2%BF%E2%80%A6%C3%A7%C5%93%E2%80%B9/peptides%C3%A5%C2%BF%E2%80%A6%C3%A7%C5%93%E2%80%B9/68-1.html#38) |
| 325 | 339 | 1776.74 | 1775.73 | 1775.85 | -0.1145 | 0 | 0 |  | Deamidated (NQ)@2;  Deamidated (NQ)@5;  Deamidated (NQ)@8 | [VNLDNITQAEIETWK](../../%C3%A6%C2%A1%C5%92%C3%A9%C2%9D%C2%A2/%C3%A6%C2%A1%C5%92%C3%A9%C2%9D%C2%A2%C3%A6%C2%9D%C2%90%C3%A6%E2%80%93%E2%84%A2/%C3%A8%C2%B4%C2%A8%C3%A8%C2%B0%C2%B1%C3%A9%E2%80%B0%C2%B4%C3%A5%C2%AE%C5%A1%C3%A7%5C%C2%BB%E2%80%9C%C3%A6%C2%9E%C5%93/F12FTSECKJ0265_005_GS_20120821/PMF_Report%C3%A5%C2%BF%E2%80%A6%C3%A7%C5%93%E2%80%B9/peptides%C3%A5%C2%BF%E2%80%A6%C3%A7%C5%93%E2%80%B9/68-1.html#48) |
| 135 | 158 | 2572.36 | 2571.35 | 2571.26 | 0.0898 | 0 |  |  | Deamidated (NQ)@2;  Deamidated (NQ)@15 | DNTPSVVHIDMVPGNHVEVQIAAK |
| 9 | AAD01872.1 | 234 | 240 | 795.41 | 794.40 | 794.41 | -0.0103 | 0 |  | 137 | - | LTGMAFR |
| 234 | 240 | 811.41 | 810.40 | 810.41 | -0.0022 | 0 |  |  | Oxidation (M)@4 | LTGMAFR |
| 31 | 40 | 1135.59 | 1134.58 | 1134.54 | 0.0441 | 0 |  |  | - | AVNDPFMDVK |
| 2 | 12 | 1163.62 | 1162.61 | 1162.54 | 0.0675 | 0 |  |  | Oxidation (M)@4;  Deamidated (NQ)@7 | PPTMGINGFGR |
| 258 | 269 | 1277.69 | 1276.68 | 1276.70 | -0.0177 | 1 |  |  | - | AAKYDDIVAAIK |
| 226 | 240 | 1590.82 | 1589.82 | 1589.86 | -0.0426 | 1 |  |  | Deamidated (NQ)@6;O  xidation (M)@12 | VIPAVNGKLTGMAFR |
| 316 | 329 | 1788.79 | 1787.79 | 1787.79 | -0.0045 | 0 | 109.31 |  | - | [LVSWYDNEWGYSNR](../../%C3%A6%C2%A1%C5%92%C3%A9%EF%86%9D%C2%A2/%C3%A6%C2%A1%C5%92%C3%A9%EF%86%9D%C2%A2%C3%A6%C2%9D%C2%90%C3%A6%E2%80%93%E2%84%A2/%C3%A8%C2%B4%C2%A8%C3%A8%C2%B0%C2%B1%C3%A9%E2%80%B0%C2%B4%C3%A5%C2%AE%C5%A1%C3%A7%5C%C2%BB%E2%80%9C%C3%A6%C2%9E%C5%93/F12FTSECKJ0265_GS_20121211_489/F12FTSECKJ0265_GS_20121211_489/F12FTSECKJ0265_GS_20121211_489/PMF_Report/peptides/9-1.html#44) |
| 201 | 221 | 1991.98 | 1990.97 | 1991.03 | -0.0591 | 0 |  |  | Deamidated (NQ)@10;  Deamidated (NQ)@11 | TGVVAAAHPQNIIPSSTGAAK |
| 20 | 40 | 2259.17 | 2258.16 | 2258.11 | 0.0438 | 1 |  |  | Deamidated (NQ)@5;  Deamidated (NQ)@14 | AALANPDIEVKAVNDPFMDVK |
| 20 | 40 | 2275.15 | 2274.15 | 2274.11 | 0.0363 | 1 |  |  | Deamidated (NQ)@5;  Deamidated (NQ)@14;  Oxidation (M)@18 | AALANPDIEVKAVNDPFMDVK |
| 45 | BAD05063.1 | 32 | 38 | 877.50 | 876.49 | 876.47 | 0.0209 | 0 |  | 75 | - | TYEVPIR |
| 438 | 445 | 925.50 | 924.49 | 924.43 | 0.0549 | 0 |  |  | Gln->pyro-Glu (N-term Q)@N  _term | QNYVGYAK |
| 304 | 312 | 1033.60 | 1032.60 | 1032.52 | 0.0794 | 0 | 0 |  | Deamidated (NQ)@4 | [MLEQIGDVK](../../%C3%A6%C2%A1%C5%92%C3%A9%EF%86%9D%C2%A2/%C3%A6%C2%A1%C5%92%C3%A9%C2%9D%C2%A2%C3%A6%C2%9D%C2%90%C3%A6%E2%80%93%E2%84%A2/%C3%A8%C2%B4%C2%A8%C3%A8%C2%B0%C2%B1%C3%A9%E2%80%B0%C2%B4%C3%A5%C2%AE%C5%A1%C3%A7%5C%C2%BB%E2%80%9C%C3%A6%C2%9E%C5%93/F12FTSECKJ0265_005_GS_20120821/PMF_Report%C3%A5%C2%BF%E2%80%A6%C3%A7%C5%93%E2%80%B9/peptides%C3%A5%C2%BF%E2%80%A6%C3%A7%C5%93%E2%80%B9/45-3.html#14) |
| 304 | 312 | 1049.58 | 1048.57 | 1048.51 | 0.0623 | 0 |  |  | Oxidation (M)@1;  Deamidated (NQ)@4 | MLEQIGDVK |
| 438 | 446 | 1081.60 | 1080.59 | 1080.54 | 0.0570 | 1 | 11.08 |  | Gln->pyro-Glu (N-term Q)@N  _term | [QNYVGYAKR](../../%C3%A6%C2%A1%C5%92%C3%A9%C2%9D%C2%A2/%C3%A6%C2%A1%C5%92%C3%A9%C2%9D%C2%A2%C3%A6%C2%9D%C2%90%C3%A6%E2%80%93%E2%84%A2/%C3%A8%C2%B4%C2%A8%C3%A8%C2%B0%C2%B1%C3%A9%E2%80%B0%C2%B4%C3%A5%C2%AE%C5%A1%C3%A7%5C%C2%BB%E2%80%9C%C3%A6%C2%9E%C5%93/F12FTSECKJ0265_005_GS_20120821/PMF_Report%C3%A5%C2%BF%E2%80%A6%C3%A7%C5%93%E2%80%B9/peptides%C3%A5%C2%BF%E2%80%A6%C3%A7%C5%93%E2%80%B9/45-3.html#19) |
| 93 | 102 | 1097.60 | 1096.60 | 1096.61 | -0.0161 | 0 | 0 |  | - | [GIPIEQLAEK](../../%C3%A6%C2%A1%C5%92%C3%A9%C2%9D%C2%A2/%C3%A6%C2%A1%C5%92%C3%A9%C2%9D%C2%A2%C3%A6%C2%9D%C2%90%C3%A6%E2%80%93%E2%84%A2/%C3%A8%C2%B4%C2%A8%C3%A8%C2%B0%C2%B1%C3%A9%E2%80%B0%C2%B4%C3%A5%C2%AE%C5%A1%C3%A7%5C%C2%BB%E2%80%9C%C3%A6%C2%9E%C5%93/F12FTSECKJ0265_005_GS_20120821/PMF_Report%C3%A5%C2%BF%E2%80%A6%C3%A7%C5%93%E2%80%B9/peptides%C3%A5%C2%BF%E2%80%A6%C3%A7%C5%93%E2%80%B9/45-3.html#20) |
| 32 | 41 | 1235.65 | 1234.65 | 1234.63 | 0.0145 | 1 |  |  | Deamidated (NQ)@8 | TYEVPIRNSR |
| 455 | 465 | 1264.72 | 1263.71 | 1263.60 | 0.1147 | 1 | 0 |  | Gln->pyro-Glu (N-term Q)@N  _term;  Deamidated (NQ)@1 | [QEAKFNLESSK](../../%C3%A6%C2%A1%C5%92%C3%A9%C2%9D%C2%A2/%C3%A6%C2%A1%C5%92%C3%A9%C2%9D%C2%A2%C3%A6%C2%9D%C2%90%C3%A6%E2%80%93%E2%84%A2/%C3%A8%C2%B4%C2%A8%C3%A8%C2%B0%C2%B1%C3%A9%E2%80%B0%C2%B4%C3%A5%C2%AE%C5%A1%C3%A7%5C%C2%BB%E2%80%9C%C3%A6%C2%9E%C5%93/F12FTSECKJ0265_005_GS_20120821/PMF_Report%C3%A5%C2%BF%E2%80%A6%C3%A7%C5%93%E2%80%B9/peptides%C3%A5%C2%BF%E2%80%A6%C3%A7%C5%93%E2%80%B9/45-3.html#26) |
| 234 | 244 | 1326.60 | 1325.60 | 1325.67 | -0.0769 | 1 |  |  | - | LNENKYSPHPK |
| 4 | 19 | 1820.04 | 1819.03 | 1818.87 | 0.1569 | 0 |  |  | Deamidated (NQ)@5;  Deamidated (NQ)@6;  Deamidated (NQ)@7 | ITDLNQQIITNETGEK |
| 213 | 233 | 2558.28 | 2557.27 | 2557.07 | 0.2015 | 0 |  |  | Deamidated (NQ)@1;  Deamidated (NQ)@3;  Oxidation (M)@18;  Oxidation (M)@19 | NFNNPSSDLPYVDNFLYMMDK |
| 248 | 269 | 2588.41 | 2587.41 | 2587.26 | 0.1488 | 0 |  |  | Deamidated (NQ)@15;  Carbamidomethyl (C)@16 | ALDILFILHAEHEMNCSTAFVR |
| 213 | 238 | 3122.45 | 3121.44 | 3121.42 | 0.0259 | 1 |  |  | - | NFNNPSSDLPYVDNFLYMMDKLNENK |
| 93 | 120 | 3136.46 | 3135.46 | 3135.70 | -0.2434 | 1 |  |  | Deamidated (NQ)@6 | GIPIEQLAEKSTFLEVAYLLIYGELPTK |
| 85* | AAB68396.1 | 172 | 179 | 842.50 | 841.49 | 841.50 | -0.0080 | 0 |  | 77 | - | LGAALIER |
| 54 | 64 | 1179.59 | 1178.58 | 1178.56 | 0.0235 | 0 |  |  | Oxidation (M)@3;  Deamidated (NQ)@8 | ISMAVADNVSR |
| 25 | 35 | 1197.56 | 1196.55 | 1196.66 | -0.1146 | 1 |  |  | Deamidated (NQ)@8 | GKPRLGDNALR |
| 29 | 40 | 1293.64 | 1292.63 | 1292.64 | -0.0044 | 1 |  |  | Deamidated (NQ)@4;  Deamidated (NQ)@11 | LGDNALRFSGNK |
| 430 | 439 | 1308.61 | 1307.60 | 1307.61 | -0.0048 | 1 |  |  | Oxidation (M)@5 | WHVYMTKDGR |
| 409 | 420 | 1359.72 | 1358.71 | 1358.65 | 0.0571 | 0 |  |  | Deamidated (NQ)@1;  Oxidation (M)@4 | QIGMFSFTGLNK |
| 384 | 396 | 1524.72 | 1523.72 | 1523.78 | -0.0680 | 1 |  |  | Deamidated (NQ)@1;  Deamidated (NQ)@4 | NVRQLLYDSISSK |
| 319 | 334 | 1571.70 | 1570.69 | 1570.83 | -0.1433 | 0 | 8.37 |  | - | [IGAINVISSSPESAAR](../../%C3%A6%C2%A1%C5%92%C3%A9%C2%9D%C2%A2/%C3%A6%C2%A1%C5%92%C3%A9%C2%9D%C2%A2%C3%A6%C2%9D%C2%90%C3%A6%E2%80%93%E2%84%A2/%C3%A8%C2%B4%C2%A8%C3%A8%C2%B0%C2%B1%C3%A9%E2%80%B0%C2%B4%C3%A5%C2%AE%C5%A1%C3%A7%5C%C2%BB%E2%80%9C%C3%A6%C2%9E%C5%93/F12FTSECKJ0265_GS_20121211_489/F12FTSECKJ0265_GS_20121211_489/F12FTSECKJ0265_GS_20121211_489/PMF_Report/peptides/85-1.html#27) |
| 205 | 216 | 1602.75 | 1601.74 | 1601.75 | -0.0133 | 1 |  |  | - | VPWSEYRYYDPK |
| 198 | 211 | 1767.82 | 1766.81 | 1766.84 | -0.0235 | 1 |  |  | Deamidated (NQ)@1 | NIFNDARVPWSEYR |
| 421 | 436 | 2044.00 | 2042.99 | 2042.88 | 0.1122 | 1 |  |  | Oxidation (M)@6;  Oxidation (M)@14 | EQSDNMTNKWHVYMTK |
| 65 | 84 | 2131.08 | 2130.07 | 2130.07 | 0.0018 | 0 |  |  | Oxidation (M)@6 | FEGIPMAPPDPILGVSEAFK |
| 402 | 420 | 2234.03 | 2233.02 | 2233.11 | -0.0885 | 1 |  |  | Deamidated (NQ)@8;  Deamidated (NQ)@18 | DWSFILKQIGMFSFTGLNK |
| 2 | 24 | 2285.05 | 2284.04 | 2284.17 | -0.1252 | 0 |  |  | - | ASSVLSGANQSSPSSTVLNTHLK |
| 409 | 429 | 2422.20 | 2421.19 | 2421.09 | 0.0991 | 1 |  |  | Oxidation (M)@4;  Oxidation (M)@18 | QIGMFSFTGLNKEQSDNMTNK |
| 54 | 84 | 3259.41 | 3258.41 | 3258.63 | -0.2228 | 1 |  |  | Deamidated (NQ)@8 | ISMAVADNVSRFEGIPMAPPDPILGVSEAFK |
| 54 | 84 | 3274.42 | 3273.41 | 3273.64 | -0.2319 | 1 |  |  | Oxidation (M)@3 | ISMAVADNVSRFEGIPMAPPDPILGVSEAFK |
| 54 | 84 | 3275.93 | 3274.93 | 3274.63 | 0.3009 | 1 |  |  | Oxidation (M)@3;  Deamidated (NQ)@8 | ISMAVADNVSRFEGIPMAPPDPILGVSEAFK |
| 54 | 84 | 3291.41 | 3290.40 | 3290.62 | -0.2160 | 1 |  |  | Oxidation (M)@3;  Deamidated (NQ)@8;  Oxidation (M)@17 | ISMAVADNVSRFEGIPMAPPDPILGVSEAFK |
| 98 | ABA55516.1 | 167 | 178 | 1391.73 | 1390.72 | 1390.73 | -0.0070 | 0 | 65.5 | 76 | Carbamidomethyl (C)@4 | [TIVCIIPSFGER](../../%C3%A6%C2%A1%C5%92%C3%A9%C2%9D%C2%A2/%C3%A6%C2%A1%C5%92%C3%A9%C2%9D%C2%A2%C3%A6%C2%9D%C2%90%C3%A6%E2%80%93%E2%84%A2/%C3%A8%C2%B4%C2%A8%C3%A8%C2%B0%C2%B1%C3%A9%E2%80%B0%C2%B4%C3%A5%C2%AE%C5%A1%C3%A7%5C%C2%BB%E2%80%9C%C3%A6%C5%BE%C5%93/F12FTSECKJ0265_GS_20121211_489/F12FTSECKJ0265_GS_20121211_489/F12FTSECKJ0265_GS_20121211_489/PMF_Report/peptides/98-4.html#28) |
| 20 | 31 | 1432.70 | 1431.69 | 1431.70 | -0.0096 | 0 |  |  | - | LLQQFENSDNPK |
| 20 | 34 | 1838.91 | 1837.90 | 1837.94 | -0.0441 | 1 |  |  | - | LLQQFENSDNPKIHR |
| 179 | 201 | 2582.43 | 2581.42 | 2581.26 | 0.1644 | 0 |  |  | - | YLSTILYANLWEEAAAQTAEPLD |
| 179 | 201 | 2584.19 | 2583.18 | 2583.23 | -0.0439 | 0 |  |  | Deamidated (NQ)@9;  Deamidated (NQ)@17 | YLSTILYANLWEEAAAQTAEPLD |
| 5 | ABA55503.1 | 201 | 208 | 935.44 | 934.43 | 934.42 | 0.0116 | 0 |  | 89 | Carbamidomethyl (C)@4 | DFPCATPK |
| 29 | 39 | 1085.55 | 1084.54 | 1084.56 | -0.0237 | 0 |  |  | - | GNGPVISAQSR |
| 161 | 177 | 1935.87 | 1934.86 | 1934.86 | -0.0037 | 0 | 81.35 |  | Carbamidomethyl (C)@2;  Carbamidomethyl (C)@5 | [ECLSCYGTASIEHPGVR](../../%C3%A6%C2%A1%C5%92%C3%A9%C2%9D%C2%A2/%C3%A6%C2%A1%C5%92%C3%A9%C2%9D%C2%A2%C3%A6%C2%9D%C2%90%C3%A6%E2%80%93%E2%84%A2/%C3%A8%C2%B4%C2%A8%C3%A8%C2%B0%C2%B1%C3%A9%E2%80%B0%C2%B4%C3%A5%C2%AE%C5%A1%C3%A7%5C%C2%BB%E2%80%9C%C3%A6%C2%9E%C5%93/F12FTSECKJ0265_GS_20121211_489/F12FTSECKJ0265_GS_20121211_489/F12FTSECKJ0265_GS_20121211_489/PMF_Report/peptides/5-3.html#76) |
| 6 | ZP_01437860.1 | 66 | 71 | 734.37 | 733.36 | 733.41 | -0.0528 | 0 |  | 92 | Deamidated (NQ)@2 | LQGFIR |
| 287 | 293 | 744.34 | 743.33 | 743.31 | 0.0252 | 0 |  |  | Gln->pyro-Glu (N-term Q)@N  _term;  Deamidated (NQ)@1;  Deamidated (NQ)@2;  Deamidated (NQ)@5 | QNAAQAR |
| 328 | 334 | 842.48 | 841.48 | 841.44 | 0.0304 | 0 |  |  | - | HPNVTFK |
| 287 | 294 | 869.51 | 868.50 | 868.45 | 0.0483 | 1 |  |  | Gln->pyro-Glu (N-term Q)@N  _term | QNAAQARK |
| 287 | 294 | 870.52 | 869.52 | 869.44 | 0.0807 | 1 |  |  | Gln->pyro-Glu (N-term Q)@N  _term;  Deamidated (NQ)@1 | QNAAQARK |
| 287 | 294 | 871.34 | 870.33 | 870.42 | -0.0850 | 1 |  |  | Gln->pyro-Glu (N-term Q)@N  _term;Deamidated (NQ)@1;  Deamidated (NQ)@2 | QNAAQARK |
| 229 | 235 | 873.50 | 872.50 | 872.47 | 0.0255 | 0 |  |  | - | LSEQLQR |
| 370 | 377 | 901.50 | 900.49 | 900.54 | -0.0499 | 1 |  |  | - | ISVKVAER |
| 228 | 235 | 1031.55 | 1030.54 | 1030.54 | 0.0035 | 1 |  |  | Deamidated (NQ)@5;  Deamidated (NQ)@7 | RLSEQLQR |
| 378 | 386 | 1037.47 | 1036.46 | 1036.48 | -0.0223 | 0 | 0 |  | Deamidated (NQ)@3;  Deamidated (NQ)@7 | [DGNLTFNVR](../../%C3%A6%C2%A1%C5%92%C3%A9%C2%9D%C2%A2/%C3%A6%C2%A1%C5%92%C3%A9%C2%9D%C2%A2%C3%A6%C2%9D%C2%90%C3%A6%E2%80%93%E2%84%A2/%C3%A8%C2%B4%C2%A8%C3%A8%C2%B0%C2%B1%C3%A9%E2%80%B0%C2%B4%C3%A5%C2%AE%C5%A1%C3%A7%5C%C2%BB%E2%80%9C%C3%A6%C2%9E%C5%93/F12FTSECKJ0265_005_GS_20120821/PMF_Report%C3%A5%C2%BF%E2%80%A6%C3%A7%C5%93%E2%80%B9/peptides%C3%A5%C2%BF%E2%80%A6%C3%A7%C5%93%E2%80%B9/6-2.html#18) |
| 72 | 80 | 1055.56 | 1054.55 | 1054.49 | 0.0640 | 1 | 0 |  | - | [SHNGDRDVR](../../%C3%A6%C2%A1%C5%92%C3%A9%C2%9D%C2%A2/%C3%A6%C2%A1%C5%92%C3%A9%C2%9D%C2%A2%C3%A6%C2%9D%C2%90%C3%A6%E2%80%93%E2%84%A2/%C3%A8%C2%B4%C2%A8%C3%A8%C2%B0%C2%B1%C3%A9%E2%80%B0%C2%B4%C3%A5%C2%AE%C5%A1%C3%A7%5C%C2%BB%E2%80%9C%C3%A6%C2%9E%C5%93/F12FTSECKJ0265_005_GS_20120821/PMF_Report%C3%A5%C2%BF%E2%80%A6%C3%A7%C5%93%E2%80%B9/peptides%C3%A5%C2%BF%E2%80%A6%C3%A7%C5%93%E2%80%B9/6-2.html#19) |
| 397 | 407 | 1176.55 | 1175.55 | 1175.61 | -0.0650 | 1 |  |  | Gln->pyro-Glu (N-term Q)@N  _term | QKSGFGIIGMR |
| 387 | 398 | 1194.53 | 1193.52 | 1193.53 | -0.0115 | 1 |  |  | - | DDGGGFADGKQK |
| 207 | 218 | 1376.65 | 1375.64 | 1375.65 | -0.0099 | 1 |  |  | Deamidated (NQ)@7;  Oxidation (M)@9 | DLARGLNQMADR |
| 102 | 118 | 1938.96 | 1937.95 | 1938.03 | -0.0747 | 0 | 0 |  | Deamidated (NQ)@4;  Deamidated (NQ)@14 | [GLPQWFVTLLSPPQPTR](../../%C3%A6%C2%A1%C5%92%C3%A9%C2%9D%C2%A2/%C3%A6%C2%A1%C5%92%C3%A9%C2%9D%C2%A2%C3%A6%C2%9D%C2%90%C3%A6%E2%80%93%E2%84%A2/%C3%A8%C2%B4%C2%A8%C3%A8%C2%B0%C2%B1%C3%A9%E2%80%B0%C2%B4%C3%A5%C2%AE%C5%A1%C3%A7%5C%C2%BB%E2%80%9C%C3%A6%C2%9E%C5%93/F12FTSECKJ0265_005_GS_20120821/PMF_Report%C3%A5%C2%BF%E2%80%A6%C3%A7%C5%93%E2%80%B9/peptides%C3%A5%C2%BF%E2%80%A6%C3%A7%C5%93%E2%80%B9/6-2.html#33) |
| 61 | EGB12199.1 | 419 | 426 | 886.48 | 885.47 | 885.50 | -0.0315 | 1 |  | 181 | - | RNTTVPAK |
| 163 | 174 | 1185.61 | 1184.60 | 1184.65 | -0.0530 | 0 | 103.89 |  | - | [DAGSIAGLNVLR](../../%C3%A6%C2%A1%C5%92%C3%A9%C2%9D%C2%A2/%C3%A6%C2%A1%C5%92%C3%A9%C2%9D%C2%A2%C3%A6%C2%9D%C2%90%C3%A6%E2%80%93%E2%84%A2/%C3%A8%C2%B4%C2%A8%C3%A8%C2%B0%C2%B1%C3%A9%E2%80%B0%C2%B4%C3%A5%C2%AE%C5%A1%C3%A7%5C%C2%BB%E2%80%9C%C3%A6%C2%9E%C5%93/F12FTSECKJ0265_005_GS_20120821/PMF_Report%C3%A5%C2%BF%E2%80%A6%C3%A7%C5%93%E2%80%B9/peptides%C3%A5%C2%BF%E2%80%A6%C3%A7%C5%93%E2%80%B9/61-6.html#25) |
| 28 | 38 | 1228.57 | 1227.56 | 1227.62 | -0.0559 | 0 | 65.74 |  | - | [VEIIANDQGNR](../../%C3%A6%C2%A1%C5%92%C3%A9%C2%9D%C2%A2/%C3%A6%C2%A1%C5%92%C3%A9%C2%9D%C2%A2%C3%A6%C2%9D%C2%90%C3%A6%E2%80%93%E2%84%A2/%C3%A8%C2%B4%C2%A8%C3%A8%C2%B0%C2%B1%C3%A9%E2%80%B0%C2%B4%C3%A5%C2%AE%C5%A1%C3%A7%5C%C2%BB%E2%80%9C%C3%A6%C5%BE%C5%93/F12FTSECKJ0265_005_GS_20120821/PMF_Report%C3%A5%C2%BF%E2%80%A6%C3%A7%C5%93%E2%80%B9/peptides%C3%A5%C2%BF%E2%80%A6%C3%A7%C5%93%E2%80%B9/61-6.html#29) |
| 462 | 472 | 1256.61 | 1255.61 | 1255.64 | -0.0323 | 0 |  |  | - | FNLDGIPPMPR |
| 223 | 238 | 1675.70 | 1674.69 | 1674.72 | -0.0350 | 0 |  |  | - | ATAGDTHLGGEDFDNR |
| 141 | 158 | 2038.89 | 2037.88 | 2038.00 | -0.1160 | 1 |  |  | Deamidated (NQ)@4;  Deamidated (NQ)@14 | EVKNAVVTVPAYFNDSQR |
| 577 | 598 | 2604.20 | 2603.19 | 2603.16 | 0.0365 | 1 |  |  | Deamidated (NQ)@11;  Oxidation (M)@13 | ITETTSWLDANQMAEKEEYEAK |
| 364 | 390 | 2665.19 | 2664.19 | 2664.28 | -0.0899 | 0 |  |  | Deamidated (NQ)@3 | SINPDEAVAYGATVQAAILSGADSSEK |
| 102 | XP_002901832.1 | 306 | 312 | 780.43 | 779.42 | 779.37 | 0.0435 | 0 |  | 82.4 | Carbamidomethyl (C)@4 | GLFCAGR |
| 559 | 569 | 1046.48 | 1045.48 | 1045.51 | -0.0328 | 1 |  |  | Oxidation (M)@6 | GGPSGMGRGVR |
| 421 | 429 | 1066.50 | 1065.49 | 1065.55 | -0.0552 | 1 |  |  | Deamidated (NQ)@1 | QYARTIEGK |
| 467 | 475 | 1086.58 | 1085.58 | 1085.51 | 0.0629 | 1 |  |  | - | HFKDPEEGK |
| 430 | 439 | 1090.52 | 1089.52 | 1089.62 | -0.1019 | 0 |  |  | - | AQLLVNAYAK |
| 38 | 47 | 1091.54 | 1090.54 | 1090.54 | -0.0077 | 1 |  |  | Oxidation (M)@8 | TTLGPRGMDK |
| 1 | 10 | 1179.60 | 1178.59 | 1178.62 | -0.0318 | 0 |  |  | Oxidation (M)@1 | MNGMMIILLK |
| 313 | 323 | 1350.71 | 1349.70 | 1349.59 | 0.1139 | 1 |  |  | Deamidated (NQ)@3;  Deamidated (NQ)@10 | VAQDDMERTQR |
| 313 | 323 | 1365.65 | 1364.64 | 1364.60 | 0.0453 | 1 |  |  | Deamidated (NQ)@3;  Oxidation (M)@6 | VAQDDMERTQR |
| 552 | 566 | 1380.68 | 1379.68 | 1379.59 | 0.0904 | 1 |  |  | Oxidation (M)@2 | GMMGGGRGGPSGMGR |
| 218 | 229 | 1402.67 | 1401.66 | 1401.66 | 0.0067 | 0 |  |  | - | TFSYAGFEQQPK |
| 152 | 165 | 1533.76 | 1532.75 | 1532.75 | 0.0030 | 1 |  |  | Gln->pyro-Glu (N-term Q)@N  _term;  Deamidated (NQ)@1;  Oxidation (M)@2 | QMLERVSGTALNSK |
| 2 | 19 | 1936.07 | 1935.06 | 1934.98 | 0.0814 | 1 |  |  | - | NGMMIILLKEGTDTSQGK |
| 447 | 464 | 1973.02 | 1972.01 | 1971.93 | 0.0851 | 0 | 16.48 |  | Gln->pyro-Glu (N-term Q)@N  _term;  Deamidated (NQ)@1;  Deamidated (NQ)@5 | [QIAENAGHDATDILNHLR](../../%C3%A6%C2%A1%C5%92%C3%A9%C2%9D%C2%A2/%C3%A6%C2%A1%C5%92%C3%A9%C2%9D%C2%A2%C3%A6%C2%9D%C2%90%C3%A6%E2%80%93%E2%84%A2/%C3%A8%C2%B4%C2%A8%C3%A8%C2%B0%C2%B1%C3%A9%E2%80%B0%C2%B4%C3%A5%C2%AE%C5%A1%C3%A7%5C%C2%BB%E2%80%9C%C3%A6%C2%9E%C5%93/F12FTSECKJ0265_GS_20121211_489/F12FTSECKJ0265_GS_20121211_489/F12FTSECKJ0265_GS_20121211_489/PMF_Report/peptides/102-1.html#57) |
| 20 | 37 | 1992.00 | 1990.99 | 1990.93 | 0.0593 | 0 | 1.55 |  | Deamidated (NQ)@2;  Deamidated (NQ)@6;  Deamidated (NQ)@8;  Carbamidomethyl (C)@10;  Deamidated (NQ)@11 | [AQLISNINACQAVMEAVR](../../%C3%A6%C2%A1%C5%92%C3%A9%EF%86%9D%C2%A2/%C3%A6%C2%A1%C5%92%C3%A9%C2%9D%C2%A2%C3%A6%C2%9D%C2%90%C3%A6%E2%80%93%E2%84%A2/%C3%A8%C2%B4%C2%A8%C3%A8%C2%B0%C2%B1%C3%A9%E2%80%B0%C2%B4%C3%A5%C2%AE%C5%A1%C3%A7%5C%C2%BB%E2%80%9C%C3%A6%C2%9E%C5%93/F12FTSECKJ0265_GS_20121211_489/F12FTSECKJ0265_GS_20121211_489/F12FTSECKJ0265_GS_20121211_489/PMF_Report/peptides/102-1.html#59) |
| 20 | 37 | 2005.99 | 2004.99 | 2004.96 | 0.0260 | 0 |  |  | Deamidated (NQ)@2;  Deamidated (NQ)@6;  Carbamidomethyl (C)@10;  Oxidation (M)@14 | AQLISNINACQAVMEAVR |
| 20 | 37 | 2008.00 | 2006.99 | 2006.93 | 0.0600 | 0 |  |  | Deamidated (NQ)@2;  Deamidated (NQ)@6;  Deamidated (NQ)@8;  Carbamidomethyl (C)@10;  Deamidated (NQ)@11;  Oxidation (M)@14 | AQLISNINACQAVMEAVR |
| 106 | 122 | 2009.00 | 2007.99 | 2008.01 | -0.0183 | 0 |  |  | Gln->pyro-Glu (N-term Q)@N  _term;  Oxidation (M)@14 | QAKPFVEENTHPQMIIK |
| 106 | 122 | 2011.98 | 2010.97 | 2010.96 | 0.0075 | 0 |  |  | Gln->pyro-Glu (N-term Q)@N  _term;  Deamidated (NQ)@1;  Deamidated (NQ)@9;  Deamidated (NQ)@13;  Oxidation (M)@14 | QAKPFVEENTHPQMIIK |
| 447 | 466 | 2248.09 | 2247.09 | 2247.08 | 0.0089 | 1 |  |  | Deamidated (NQ)@1;  Deamidated (NQ)@5;  Deamidated (NQ)@15;  Deamidated (NQ)@19 | QIAENAGHDATDILNHLRQK |
| 106 | 125 | 2383.97 | 2382.97 | 2383.20 | -0.2344 | 1 |  |  | Gln->pyro-Glu (N-term Q)@N  _term;  Deamidated (NQ)@1 | QAKPFVEENTHPQMIIKSFR |
| 172 | 197 | 2811.42 | 2810.41 | 2810.41 | -0.0044 | 0 |  |  | Deamidated (NQ)@1 | QFFSPMIVDAVLSLDEGLDISMVGVK |
| 440 | 464 | 2814.42 | 2813.41 | 2813.46 | -0.0465 | 1 |  |  | - | AFEIIPRQIAENAGHDATDILNHLR |
| 87 | NP_001185317.1 | 239 | 245 | 870.53 | 869.53 | 869.46 | 0.0656 | 0 |  | 75.9 | - | QLDIPER |
| 204 | 211 | 875.46 | 874.45 | 874.45 | 0.0005 | 1 |  |  | Deamidated (NQ)@6;  Deamidated (NQ)@7 | KGLASNQR |
| 1122 | 1129 | 935.47 | 934.46 | 934.44 | 0.0178 | 0 |  |  | Carbamidomethyl (C)@7 | EGDILTCK |
| 725 | 733 | 1092.51 | 1091.50 | 1091.44 | 0.0617 | 0 |  |  | Deamidated (NQ)@2;  Deamidated (NQ)@3;Deamidated (NQ)@5;  Deamidated (NQ)@7 | SQNVNDQQR |
| 936 | 945 | 1093.67 | 1092.66 | 1092.61 | 0.0511 | 0 |  |  | - | VFVNAAGFLR |
| 926 | 935 | 1127.55 | 1126.54 | 1126.62 | -0.0786 | 1 |  |  | Oxidation (M)@4 | DLIMHGLGKK |
| 205 | 214 | 1161.61 | 1160.61 | 1160.57 | 0.0344 | 1 |  |  | - | GLASNQRMER |
| 205 | 214 | 1163.64 | 1162.64 | 1162.54 | 0.0961 | 1 |  |  | Deamidated (NQ)@5;  Deamidated (NQ)@6 | GLASNQRMER |
| 205 | 214 | 1179.60 | 1178.59 | 1178.53 | 0.0556 | 1 |  |  | Deamidated (NQ)@5;  Deamidated (NQ)@6;  Oxidation (M)@8 | GLASNQRMER |
| 289 | 299 | 1193.61 | 1192.60 | 1192.61 | -0.0077 | 1 |  |  | - | GFSVNKDDIAK |
| 725 | 734 | 1219.62 | 1218.61 | 1218.55 | 0.0667 | 1 |  |  | Deamidated (NQ)@2;  Deamidated (NQ)@3;  Deamidated (NQ)@5 | SQNVNDQQRK |
| 176 | 186 | 1313.64 | 1312.63 | 1312.62 | 0.0122 | 1 |  |  | Oxidation (M)@10 | YRQGSDITAMR |
| 568 | 578 | 1333.73 | 1332.72 | 1332.71 | 0.0167 | 0 |  |  | Deamidated (NQ)@5 | FEGAQWLLIQK |
| 324 | 338 | 1537.74 | 1536.73 | 1536.71 | 0.0273 | 0 |  |  | - | SLLDTGDFDGANQGK |
| 1380 | 1393 | 1578.84 | 1577.83 | 1577.77 | 0.0649 | 0 |  |  | Deamidated (NQ)@7 | HIDDPLQESAPSIR |
| 853 | 865 | 1596.90 | 1595.89 | 1595.82 | 0.0685 | 0 |  |  | Deamidated (NQ)@6 | LHPLENFLQLDEK |
| 30 | 43 | 1717.85 | 1716.84 | 1716.77 | 0.0677 | 1 |  |  | Oxidation (M)@1;  Carbamidomethyl (C)@8 | MMMTKMQCLDLATK |
| 1138 | 1150 | 1719.86 | 1718.85 | 1718.80 | 0.0498 | 1 |  |  | Deamidated (NQ)@2;  Carbamidomethyl (C)@7;  Oxidation (M)@12 | YQVFLICKESEMR |
| 1039 | 1052 | 1782.93 | 1781.92 | 1781.86 | 0.0634 | 1 |  |  | Carbamidomethyl (C)@4 | ELSCGFQDWRIPFK |
| 1366 | 1379 | 1819.86 | 1818.85 | 1818.86 | -0.0087 | 1 |  |  | Oxidation (M)@1;  Deamidated (NQ)@13 | MFEDIDRLVAYFQR |
| 584 | 598 | 1865.91 | 1864.90 | 1864.99 | -0.0841 | 1 |  |  | - | LLQVTFKLPENYMNR |
| 389 | 404 | 1911.03 | 1910.02 | 1910.04 | -0.0170 | 1 | 0 |  | Deamidated (NQ)@2;  Deamidated (NQ)@4 | [LNLNQYLFESVIKSLK](../../%C3%A6%C2%A1%C5%92%C3%A9%EF%86%9D%C2%A2/%C3%A6%C2%A1%C5%92%C3%A9%C2%9D%C2%A2%C3%A6%C2%9D%C2%90%C3%A6%E2%80%93%E2%84%A2/%C3%A8%C2%B4%C2%A8%C3%A8%C2%B0%C2%B1%C3%A9%E2%80%B0%C2%B4%C3%A5%C2%AE%C5%A1%C3%A7%5C%C2%BB%E2%80%9C%C3%A6%C2%9E%C5%93/F12FTSECKJ0265_GS_20121211_489/F12FTSECKJ0265_GS_20121211_489/F12FTSECKJ0265_GS_20121211_489/PMF_Report/peptides/87-5.html#64) |
| 187 | 203 | 1948.95 | 1947.95 | 1947.98 | -0.0325 | 1 |  |  | Deamidated (NQ)@3 | DANEIFGDVDELLTIRK |
| 1345 | 1361 | 1988.00 | 1987.00 | 1987.00 | 0.0012 | 1 |  |  | - | STNPHHEYIGLYPKGFK |
| 1104 | 1121 | 2050.97 | 2049.96 | 2049.95 | 0.0094 | 1 |  |  | - | EDFSDDGRDIVDLADQLK |
| 229 | 245 | 2083.00 | 2081.99 | 2081.92 | 0.0705 | 1 |  |  | Oxidation (M)@2;  Deamidated (NQ)@5;  Deamidated (NQ)@11 | YMTGNDDEIRQLDIPER |
| 324 | 343 | 2120.96 | 2119.95 | 2120.04 | -0.0905 | 1 |  |  | - | SLLDTGDFDGANQGKKPETK |
| 1084 | 1103 | 2178.14 | 2177.13 | 2177.10 | 0.0286 | 1 |  |  | Deamidated (NQ)@2;  Carbamidomethyl (C)@8 | LQNGRAICVLDSGLTGMLMK |
| 1084 | 1103 | 2211.09 | 2210.08 | 2210.07 | 0.0060 | 1 |  |  | Deamidated (NQ)@2;  Deamidated (NQ)@3;  Carbamidomethyl (C)@8;  Oxidation (M)@17;  Oxidation (M)@19 | LQNGRAICVLDSGLTGMLMK |
| 783 | 802 | 2233.07 | 2232.07 | 2232.04 | 0.0287 | 0 |  |  | Oxidation (M)@6 | DVGHGMDDLSIVYVDESLPR |
| 56 | 74 | 2239.12 | 2238.11 | 2238.01 | 0.1061 | 1 |  |  | Deamidated (NQ)@14;  Deamidated (NQ)@16;  Deamidated (NQ)@17 | KDEGLDEDDYLLLQDNNVK |
| 1031 | 1048 | 2276.08 | 2275.07 | 2275.02 | 0.0474 | 1 |  |  | Deamidated (NQ)@5;  Carbamidomethyl (C)@12;  Deamidated (NQ)@15 | ETYSNIIRELSCGFQDWR |
| 803 | 822 | 2279.05 | 2278.04 | 2278.07 | -0.0321 | 1 |  |  | Deamidated (NQ)@4;  Deamidated (NQ)@11;  Deamidated (NQ)@14;  Deamidated (NQ)@15 | LYENSRISGEQLPQQSGNVR |
| 57 | 76 | 2383.95 | 2382.94 | 2383.11 | -0.1679 | 1 |  |  | Deamidated (NQ)@13 | DEGLDEDDYLLLQDNNVKFK |
| 9 | 28 | 2506.23 | 2505.22 | 2505.06 | 0.1599 | 1 |  |  | Oxidation (M)@1;  Oxidation (M)@8;  Oxidation (M)@14;  Oxidation (M)@15;  Oxidation (M)@18 | MTTESQFMEIQLSMMKTMMK |
| 1399 | 1425 | 2705.15 | 2704.14 | 2704.16 | -0.0158 | 1 |  |  | Oxidation (M)@3;  Deamidated (NQ)@21 | VPMRSPADHGSSGGSGWGSSQSEGGWK |
| 20 | AEB21848.1 | 306 | 312 | 864.47 | 863.46 | 863.51 | -0.0476 | 1 |  | 75.1 | Deamidated (NQ)@5 | SIFKQIK |
| 213 | 222 | 1045.58 | 1044.57 | 1044.48 | 0.0871 | 0 |  |  | Gln->pyro-Glu (N-term Q)@N_term | QGTQSGESIR |
| 213 | 222 | 1046.57 | 1045.57 | 1045.47 | 0.0980 | 0 |  |  | Gln->pyro-Glu (N-term Q)@N  _term;  Deamidated (NQ)@1 | QGTQSGESIR |
| 126 | 135 | 1215.59 | 1214.58 | 1214.57 | 0.0161 | 1 |  |  | - | ASEERYAFSR |
| 72 | 81 | 1228.64 | 1227.64 | 1227.62 | 0.0117 | 1 |  |  | Deamidated (NQ)@1 | NEVGIWPEKR |
| 256 | 266 | 1328.66 | 1327.65 | 1327.64 | 0.0103 | 1 |  |  | Oxidation (M)@2;  Deamidated (NQ)@3 | KMQSLFSNQTK |
| 192 | 205 | 1358.65 | 1357.64 | 1357.72 | -0.0796 | 0 | 0 |  | Deamidated (NQ)@5 | [SPVAQGLAGTSTIR](../../%C3%A6%C2%A1%C5%92%C3%A9%EF%86%9D%C2%A2/%C3%A6%C2%A1%C5%92%C3%A9%EF%86%9D%C2%A2%C3%A6%EF%86%9D%EF%86%90%C3%A6%E2%80%93%E2%84%A2/%C3%A8%C2%B4%C2%A8%C3%A8%C2%B0%C2%B1%C3%A9%E2%80%B0%C2%B4%C3%A5%C2%AE%C5%A1%C3%A7%5C%C2%BB%E2%80%9C%C3%A6%C2%9E%C5%93/F12FTSECKJ0265_GS_20121211_489/F12FTSECKJ0265_GS_20121211_489/F12FTSECKJ0265_GS_20121211_489/PMF_Report/peptides/20-6.html#26) |
| 181 | 191 | 1365.65 | 1364.65 | 1364.59 | 0.0545 | 0 |  |  | Deamidated (NQ)@1;  Deamidated (NQ)@2;  Deamidated (NQ)@4;  Deamidated (NQ)@10 | NQIQFDPMVNR |
| 89 | 100 | 1432.74 | 1431.74 | 1431.74 | -0.0018 | 1 |  |  | Deamidated (NQ)@5;Oxidation (M)@7 | SPSIQKMDQLLR |
| 48 | 62 | 1520.73 | 1519.73 | 1519.85 | -0.1213 | 1 |  |  | Deamidated (NQ)@2 | HQGLGALPSVKSIGR |
| 43 | 58 | 1677.93 | 1676.92 | 1676.93 | -0.0108 | 1 |  |  | - | LSQSRHQGLGALPSVK |
| 223 | 237 | 1724.94 | 1723.93 | 1723.88 | 0.0481 | 0 |  |  | Gln->pyro-Glu (N-term Q)@N  _term;  Deamidated (NQ)@1 | QPGSTLYFDAIIFLR |
| 267 | 281 | 1731.92 | 1730.92 | 1731.00 | -0.0887 | 1 |  |  | Deamidated (NQ)@2 | TNTLIRPVKIASVYR |
| 4 | 20 | 1931.99 | 1930.98 | 1930.93 | 0.0518 | 0 |  |  | - | DYFGSIRPPTGNTFGFR |
| 192 | 212 | 2069.04 | 2068.03 | 2068.12 | -0.0858 | 1 |  |  | Deamidated (NQ)@5 | SPVAQGLAGTSTIREAIPTAK |
| 257 | 275 | 2225.12 | 2224.12 | 2224.14 | -0.0256 | 1 |  |  | Oxidation (M)@1;  Deamidated (NQ)@2;  Deamidated (NQ)@7;  Deamidated (NQ)@8 | MQSLFSNQTKTNTLIRPVK |
| 181 | 205 | 2705.18 | 2704.17 | 2704.30 | -0.1333 | 1 |  |  | Deamidated (NQ)@1;  Deamidated (NQ)@2;  Deamidated (NQ)@4;  Deamidated (NQ)@10;  Deamidated (NQ)@16 | NQIQFDPMVNRSPVAQGLAGTSTIR |
| 181 | 205 | 2717.10 | 2716.09 | 2716.36 | -0.2669 | 1 |  |  | Deamidated (NQ)@1;  Oxidation (M)@8 | NQIQFDPMVNRSPVAQGLAGTSTIR |
| 341 | 370 | 3289.68 | 3288.67 | 3288.62 | 0.0546 | 1 |  |  | Deamidated (NQ)@12 | YGETSLHVFSDQIDYAKTQASTPYGILGVK |
| 166 | 191 | 3321.68 | 3320.67 | 3320.52 | 0.1515 | 1 |  |  | Deamidated (NQ)@9;  Deamidated (NQ)@13;  Deamidated (NQ)@16;  Deamidated (NQ)@17;D  eamidated (NQ)@19 | AFLDYFVMQYFFNLRNQIQFDPMVNR |
| 37* | AAV34146.1 | 38 | 50 | 1433.74 | 1432.73 | 1432.81 | -0.0734 | 0 |  | 201 | Gln->pyro-Glu (N-term Q)@N  _term;  Deamidated (NQ)@1 | QVGVPHLVVFLNK |
| 187 | 202 | 1688.87 | 1687.86 | 1687.85 | 0.0194 | 0 |  |  | Deamidated (NQ)@10 | TLEEGMAGDNVGILIR |
| 187 | 202 | 1703.86 | 1702.85 | 1702.86 | -0.0039 | 0 | 68.29 |  | Oxidation (M)@6 | [TLEEGMAGDNVGILIR](../../%C3%A6%C2%A1%C5%92%C3%A9%C2%9D%C2%A2/%C3%A6%C2%A1%C5%92%C3%A9%C2%9D%C2%A2%C3%A6%C2%9D%C2%90%C3%A6%E2%80%93%E2%84%A2/%C3%A8%C2%B4%C2%A8%C3%A8%C2%B0%C2%B1%C3%A9%E2%80%B0%C2%B4%C3%A5%C2%AE%C5%A1%C3%A7%5C%C2%BB%E2%80%9C%C3%A6%C2%9E%C5%93/F12FTSECKJ0265_GS_20121211_489/F12FTSECKJ0265_GS_20121211_489/F12FTSECKJ0265_GS_20121211_489/PMF_Report/peptides/37-1.html#31) |
| 111 | 127 | 2023.02 | 2022.02 | 2022.01 | 0.0020 | 1 |  |  | - | IFDLMKAVDDYIPTPER |
| 111 | 127 | 2039.01 | 2038.00 | 2038.01 | -0.0044 | 1 |  |  | Oxidation (M)@5 | IFDLMKAVDDYIPTPER |
| 187 | 206 | 2114.14 | 2113.13 | 2113.12 | 0.0139 | 1 |  |  | - | TLEEGMAGDNVGILIRGIQK |
| 76 | AAY46275.1 | 2 | 9 | 952.48 | 951.48 | 951.50 | -0.0267 | 0 |  | 170 | - | VHLTPEEK |
| 97 | 105 | 1126.53 | 1125.52 | 1125.56 | -0.0333 | 0 | 66.14 |  | - | [LHVDPENFR](../../%C3%A6%C2%A1%C5%92%C3%A9%EF%86%9D%C2%A2/%C3%A6%C2%A1%C5%92%C3%A9%EF%86%9D%C2%A2%C3%A6%EF%86%9D%EF%86%90%C3%A6%E2%80%93%E2%84%A2/%C3%A8%C2%B4%C2%A8%C3%A8%C2%B0%C2%B1%C3%A9%E2%80%B0%C2%B4%C3%A5%C2%AE%C5%A1%C3%A7%5C%C2%BB%E2%80%9C%C3%A6%C2%9E%C5%93/F12FTSECKJ0265_005_GS_20120821/PMF_Report%C3%A5%C2%BF%E2%80%A6%C3%A7%C5%93%E2%80%B9/peptides%C3%A5%C2%BF%E2%80%A6%C3%A7%C5%93%E2%80%B9/76-1.html#27) |
| 32 | 41 | 1306.67 | 1305.66 | 1305.69 | -0.0261 | 0 | 16.14 |  | Carbamidomethyl (C)@9 | [LLVVYPWTCR](../../%C3%A6%C2%A1%C5%92%C3%A9%C2%9D%C2%A2/%C3%A6%C2%A1%C5%92%C3%A9%C2%9D%C2%A2%C3%A6%C2%9D%C2%90%C3%A6%E2%80%93%E2%84%A2/%C3%A8%C2%B4%C2%A8%C3%A8%C2%B0%C2%B1%C3%A9%E2%80%B0%C2%B4%C3%A5%C2%AE%C5%A1%C3%A7%5C%C2%BB%E2%80%9C%C3%A6%C2%9E%C5%93/F12FTSECKJ0265_005_GS_20120821/PMF_Report%C3%A5%C2%BF%E2%80%A6%C3%A7%C5%93%E2%80%B9/peptides%C3%A5%C2%BF%E2%80%A6%C3%A7%C5%93%E2%80%B9/76-1.html#39) |
| 19 | 31 | 1314.63 | 1313.62 | 1313.66 | -0.0382 | 0 | 55.77 |  | - | [VNVDEVGGEALGR](../../%C3%A6%C2%A1%C5%92%C3%A9%C2%9D%C2%A2/%C3%A6%C2%A1%C5%92%C3%A9%C2%9D%C2%A2%C3%A6%C2%9D%C2%90%C3%A6%E2%80%93%E2%84%A2/%C3%A8%C2%B4%C2%A8%C3%A8%C2%B0%C2%B1%C3%A9%E2%80%B0%C2%B4%C3%A5%C2%AE%C5%A1%C3%A7%5C%C2%BB%E2%80%9C%C3%A6%C2%9E%C5%93/F12FTSECKJ0265_005_GS_20120821/PMF_Report%C3%A5%C2%BF%E2%80%A6%C3%A7%C5%93%E2%80%B9/peptides%C3%A5%C2%BF%E2%80%A6%C3%A7%C5%93%E2%80%B9/76-1.html#40) |
| 68 | 83 | 1669.84 | 1668.83 | 1668.88 | -0.0544 | 0 |  |  | - | VLGAFSDGLAHLDNLK |
| 26 | ACP30568.1 | 250 | 258 | 997.54 | 996.54 | 996.48 | 0.0612 | 0 |  | 78 | - | ISLDDYGSK |
| 707 | 716 | 1106.58 | 1105.57 | 1105.58 | -0.0122 | 0 |  |  | - | LTNISMNVSK |
| 508 | 517 | 1203.65 | 1202.65 | 1202.58 | 0.0645 | 0 |  |  | Gln->pyro-Glu (N-term Q)@N  _term;  Deamidated (NQ)@1 | QFLVDAEEIR |
| 601 | 610 | 1210.55 | 1209.55 | 1209.64 | -0.0931 | 0 |  |  | - | FSAEFLVELR |
| 642 | 653 | 1315.75 | 1314.74 | 1314.67 | 0.0712 | 0 | 0 |  | Deamidated (NQ)@4;  Deamidated (NQ)@7 | [EIPNLSNATNLK](../../%C3%A6%C2%A1%C5%92%C3%A9%EF%86%9D%C2%A2/%C3%A6%C2%A1%C5%92%C3%A9%EF%86%9D%C2%A2%C3%A6%EF%86%9D%EF%86%90%C3%A6%E2%80%93%E2%84%A2/%C3%A8%C2%B4%C2%A8%C3%A8%C2%B0%C2%B1%C3%A9%E2%80%B0%C2%B4%C3%A5%C2%AE%C5%A1%C3%A7%5C%C2%BB%E2%80%9C%C3%A6%C2%9E%C5%93/F12FTSECKJ0265_GS_20121211_489/F12FTSECKJ0265_GS_20121211_489/F12FTSECKJ0265_GS_20121211_489/PMF_Report/peptides/26-1.html#22) |
| 1 | 11 | 1353.69 | 1352.68 | 1352.58 | 0.1060 | 1 |  |  | Oxidation (M)@1;  Deamidated (NQ)@9 | MESSSPSRNWR |
| 155 | 165 | 1379.67 | 1378.66 | 1378.61 | 0.0576 | 0 |  |  | Deamidated (NQ)@3;  Oxidation (M)@8 | LWNNEADMIEK |
| 887 | 897 | 1395.72 | 1394.71 | 1394.76 | -0.0525 | 0 |  |  | Oxidation (M)@4 | VVHMIQRPFPR |
| 482 | 493 | 1466.81 | 1465.80 | 1465.76 | 0.0382 | 1 |  |  | Oxidation (M)@3 | IHMHSLLQKMGR |
| 552 | 563 | 1493.74 | 1492.73 | 1492.74 | -0.0053 | 0 |  |  | Deamidated (NQ)@4 | GMHNLQFLEIYK |
| 601 | 612 | 1513.81 | 1512.81 | 1512.78 | 0.0309 | 1 |  |  | Oxidation (M)@11 | FSAEFLVELRMR |
| 584 | 595 | 1570.83 | 1569.83 | 1569.82 | 0.0037 | 1 |  |  | - | LRLLHWDSFPMR |
| 552 | 564 | 1638.86 | 1637.85 | 1637.81 | 0.0411 | 1 |  |  | Oxidation (M)@2;  Deamidated (NQ)@4;  Deamidated (NQ)@6 | GMHNLQFLEIYKK |
| 230 | 244 | 1707.78 | 1706.78 | 1706.83 | -0.0587 | 0 |  |  | Deamidated (NQ)@6;  Oxidation (M)@11;  Deamidated (NQ)@13 | LSPIFQHSAFMGNIK |
| 12 | 25 | 1716.84 | 1715.83 | 1715.80 | 0.0364 | 1 |  |  | Deamidated (NQ)@2;  Carbamidomethyl (C)@8 | FNVFPSFCGEDLRK |
| 988 | 1002 | 1725.83 | 1724.82 | 1724.86 | -0.0354 | 1 |  |  | Oxidation (M)@1;  Carbamidomethyl (C)@6;  Deamidated (NQ)@7 | MEIIGCQFTVLGGKR |
| 348 | 362 | 1741.83 | 1740.82 | 1740.84 | -0.0197 | 0 |  |  | Carbamidomethyl (C)@8;  Deamidated (NQ)@9 | LEALEIFCQSAFGQK |
| 549 | 563 | 1840.00 | 1838.99 | 1838.94 | 0.0547 | 1 | 0 |  | Deamidated (NQ)@7 | [AFKGMHNLQFLEIYK](../../%C3%A6%C2%A1%C5%92%C3%A9%C2%9D%C2%A2/%C3%A6%C2%A1%C5%92%C3%A9%C2%9D%C2%A2%C3%A6%C2%9D%C2%90%C3%A6%E2%80%93%E2%84%A2/%C3%A8%C2%B4%C2%A8%C3%A8%C2%B0%C2%B1%C3%A9%E2%80%B0%C2%B4%C3%A5%C2%AE%C5%A1%C3%A7%5C%C2%BB%E2%80%9C%C3%A6%C2%9E%C5%93/F12FTSECKJ0265_GS_20121211_489/F12FTSECKJ0265_GS_20121211_489/F12FTSECKJ0265_GS_20121211_489/PMF_Report/peptides/26-1.html#55) |
| 491 | 506 | 1872.91 | 1871.90 | 1871.85 | 0.0526 | 1 |  |  | Oxidation (M)@1;  Carbamidomethyl (C)@7;  Deamidated (NQ)@8;  Deamidated (NQ)@9 | MGREIVCQQSVHEPGK |
| 508 | 523 | 1888.90 | 1887.89 | 1887.94 | -0.0512 | 1 |  |  | Gln->pyro-Glu (N-term Q)@N  _term;  Carbamidomethyl (C)@15 | QFLVDAEEIRDVLACK |
| 984 | 1001 | 1995.84 | 1994.83 | 1995.02 | -0.1855 | 1 |  |  | Deamidated (NQ)@2;  Oxidation (M)@5;  Carbamidomethyl (C)@10 | ANIKMEIIGCQFTVLGGK |
| 699 | 716 | 2117.00 | 2115.99 | 2115.98 | 0.0115 | 1 |  |  | Carbamidomethyl (C)@2;  Oxidation (M)@3;  Carbamidomethyl (C)@6 | VCMTQCSKLTNISMNVSK |
| 292 | 310 | 2129.06 | 2128.05 | 2128.17 | -0.1156 | 0 | 0 |  | - | [VFVVLDDVDELEQLIALAK](../../%C3%A6%C2%A1%C5%92%C3%A9%C2%9D%C2%A2/%C3%A6%C2%A1%C5%92%C3%A9%C2%9D%C2%A2%C3%A6%C2%9D%C2%90%C3%A6%E2%80%93%E2%84%A2/%C3%A8%C2%B4%C2%A8%C3%A8%C2%B0%C2%B1%C3%A9%E2%80%B0%C2%B4%C3%A5%C2%AE%C5%A1%C3%A7%5C%C2%BB%E2%80%9C%C3%A6%C2%9E%C5%93/F12FTSECKJ0265_GS_20121211_489/F12FTSECKJ0265_GS_20121211_489/F12FTSECKJ0265_GS_20121211_489/PMF_Report/peptides/26-1.html#68) |
| 699 | 716 | 2134.01 | 2133.00 | 2132.96 | 0.0450 | 1 |  |  | Carbamidomethyl (C)@2;  Oxidation (M)@3;  Deamidated (NQ)@5;  Carbamidomethyl (C)@6;  Oxidation (M)@14 | VCMTQCSKLTNISMNVSK |
| 473 | 490 | 2172.06 | 2171.06 | 2171.14 | -0.0869 | 1 |  |  | - | SLIDTHWGRIHMHSLLQK |
| 453 | 472 | 2195.94 | 2194.93 | 2195.13 | -0.1931 | 1 |  |  | - | MLLENSNLDVDHGLKALADK |
| 453 | 472 | 2197.94 | 2196.93 | 2197.09 | -0.1586 | 1 |  |  | Deamidated (NQ)@5;  Deamidated (NQ)@7 | MLLENSNLDVDHGLKALADK |
| 830 | 848 | 2258.27 | 2257.26 | 2257.04 | 0.2241 | 0 |  |  | Deamidated (NQ)@7;  Carbamidomethyl (C)@11;  Carbamidomethyl (C)@17 | IHGSFHNPDICLNFANCLK |
| 225 | 244 | 2312.24 | 2311.24 | 2311.17 | 0.0694 | 1 |  |  | Deamidated (NQ)@11;  Oxidation (M)@16;  Deamidated (NQ)@18 | ALYTRLSPIFQHSAFMGNIK |
| 75 | 93 | 2320.19 | 2319.19 | 2319.02 | 0.1634 | 0 |  |  | Deamidated (NQ)@1;  Carbamidomethyl (C)@8;  Carbamidomethyl (C)@18 | NYASSTWCLNELLEIMSCK |
| 878 | 897 | 2339.15 | 2338.15 | 2338.21 | -0.0643 | 1 |  |  | Deamidated (NQ)@2;  Deamidated (NQ)@15 | DQTSGDLLKVVHMIQRPFPR |
| 864 | 886 | 2585.30 | 2584.29 | 2584.23 | 0.0678 | 1 |  |  | Deamidated (NQ)@9;  Oxidation (M)@12;  Deamidated (NQ)@16 | YTILPGEEQPGMFKDQTSGDLLK |
| 805 | 829 | 2872.37 | 2871.36 | 2871.42 | -0.0565 | 1 |  |  | Deamidated (NQ)@7;  Carbamidomethyl (C)@20 | KLTSLPQLPESLSELNAQECESLER |
| 72 | ABG00024.1 | 142 | 147 | 777.46 | 776.45 | 776.39 | 0.0667 | 0 |  | 74 | Oxidation (M)@2;  Deamidated (NQ)@4 | LMINNR |
| 502 | 507 | 826.51 | 825.50 | 825.47 | 0.0295 | 1 |  |  | - | LYQKFK |
| 944 | 951 | 937.44 | 936.44 | 936.43 | 0.0055 | 0 |  |  | - | FGETDNVR |
| 942 | 951 | 1176.61 | 1175.60 | 1175.56 | 0.0413 | 1 |  |  | Gln->pyro-Glu (N-term Q)@N_term | QKFGETDNVR |
| 375 | 384 | 1179.61 | 1178.60 | 1178.64 | -0.0400 | 1 |  |  | - | DALSRIVYSR |
| 44 | 55 | 1277.63 | 1276.62 | 1276.70 | -0.0837 | 1 |  |  | Deamidated (NQ)@5 | KVTINVGSAYPK |
| 1153 | 1163 | 1294.61 | 1293.60 | 1293.68 | -0.0812 | 1 |  |  | - | RTQTQTSFLGR |
| 958 | 969 | 1318.63 | 1317.62 | 1317.64 | -0.0222 | 0 |  |  | - | ATDAESQINELK |
| 506 | 516 | 1344.76 | 1343.75 | 1343.70 | 0.0522 | 1 | 0 |  | - | [FKDNPHFSKPK](../../%C3%A6%C2%A1%C5%92%C3%A9%EF%86%9D%C2%A2/%C3%A6%C2%A1%C5%92%C3%A9%EF%86%9D%C2%A2%C3%A6%EF%86%9D%EF%86%90%C3%A6%E2%80%93%E2%84%A2/%C3%A8%C2%B4%C2%A8%C3%A8%C2%B0%C2%B1%C3%A9%E2%80%B0%C2%B4%C3%A5%C2%AE%C5%A1%C3%A7%5C%C2%BB%E2%80%9C%C3%A6%C2%9E%C5%93/F12FTSECKJ0265_GS_20121211_489/F12FTSECKJ0265_GS_20121211_489/F12FTSECKJ0265_GS_20121211_489/PMF_Report/peptides/72-1.html#21) |
| 979 | 990 | 1382.72 | 1381.71 | 1381.69 | 0.0138 | 0 |  |  | - | LNSTEAENHVLR |
| 148 | 162 | 1417.79 | 1416.78 | 1416.75 | 0.0327 | 0 | 0 |  | Deamidated (NQ)@3 | [ISQAILVSGESGAGK](../../%C3%A6%C2%A1%C5%92%C3%A9%C2%9D%C2%A2/%C3%A6%C2%A1%C5%92%C3%A9%C2%9D%C2%A2%C3%A6%C2%9D%C2%90%C3%A6%E2%80%93%E2%84%A2/%C3%A8%C2%B4%C2%A8%C3%A8%C2%B0%C2%B1%C3%A9%E2%80%B0%C2%B4%C3%A5%C2%AE%C5%A1%C3%A7%5C%C2%BB%E2%80%9C%C3%A6%C2%9E%C5%93/F12FTSECKJ0265_GS_20121211_489/F12FTSECKJ0265_GS_20121211_489/F12FTSECKJ0265_GS_20121211_489/PMF_Report/peptides/72-1.html#29) |
| 435 | 445 | 1425.74 | 1424.73 | 1424.73 | -0.0031 | 0 |  |  | - | LQQHFNQHVFK |
| 229 | 241 | 1462.73 | 1461.73 | 1461.83 | -0.1027 | 1 |  |  | - | ISGAAIRTYLLER |
| 1252 | 1264 | 1535.83 | 1534.82 | 1534.74 | 0.0796 | 0 |  |  | Deamidated (NQ)@3;  Carbamidomethyl (C)@6 | TLQENCVPSIFAR |
| 928 | 941 | 1575.76 | 1574.76 | 1574.82 | -0.0649 | 0 |  |  | - | ALVVSLETNIEEMK |
| 699 | 713 | 1607.79 | 1606.78 | 1606.88 | -0.0959 | 1 |  |  | Deamidated (NQ)@7 | MLLGKANLTGYQIGK |
| 996 | 1008 | 1612.81 | 1611.81 | 1611.75 | 0.0561 | 0 |  |  | Deamidated (NQ)@5;  Oxidation (M)@6;  Deamidated (NQ)@10 | TRPDNMPLLNMHR |
| 996 | 1008 | 1628.79 | 1627.78 | 1627.74 | 0.0401 | 0 |  |  | Deamidated (NQ)@5;  Oxidation (M)@6;  Deamidated (NQ)@10;  Oxidation (M)@11 | TRPDNMPLLNMHR |
| 1192 | 1206 | 1741.84 | 1740.83 | 1740.89 | -0.0554 | 0 |  |  | Oxidation (M)@13 | QQLTAFVEGLYGMIR |
| 926 | 941 | 1817.81 | 1816.81 | 1816.99 | -0.1781 | 1 |  |  | Deamidated (NQ)@11 | LKALVVSLETNIEEMK |
| 928 | 943 | 1831.94 | 1830.93 | 1830.98 | -0.0468 | 1 |  |  | - | ALVVSLETNIEEMKQK |
| 45 | 61 | 1833.81 | 1832.80 | 1832.93 | -0.1250 | 1 |  |  | - | VTINVGSAYPKDTESPR |
| 70 | 85 | 1838.91 | 1837.90 | 1838.00 | -0.1009 | 1 |  |  | Deamidated (NQ)@11 | LAYLHEPGVLQNLKSR |
| 420 | 434 | 1847.94 | 1846.93 | 1846.79 | 0.1366 | 0 | 0 |  | Deamidated (NQ)@2;  Deamidated (NQ)@6;  Carbamidomethyl (C)@8;  Deamidated (NQ)@10 | [TNSFEQFCINLTNEK](../../%C3%A6%C2%A1%C5%92%C3%A9%C2%9D%C2%A2/%C3%A6%C2%A1%C5%92%C3%A9%C2%9D%C2%A2%C3%A6%C2%9D%C2%90%C3%A6%E2%80%93%E2%84%A2/%C3%A8%C2%B4%C2%A8%C3%A8%C2%B0%C2%B1%C3%A9%E2%80%B0%C2%B4%C3%A5%C2%AE%C5%A1%C3%A7%5C%C2%BB%E2%80%9C%C3%A6%C2%9E%C5%93/F12FTSECKJ0265_GS_20121211_489/F12FTSECKJ0265_GS_20121211_489/F12FTSECKJ0265_GS_20121211_489/PMF_Report/peptides/72-1.html#59) |
| 1099 | 1115 | 1986.81 | 1985.81 | 1985.98 | -0.1707 | 1 |  |  | Deamidated (NQ)@2;  Deamidated (NQ)@9;  Oxidation (M)@15;  Deamidated (NQ)@16 | TNVFDRLIQIFGSAMQK |
| 168 | 185 | 2003.81 | 2002.81 | 2002.93 | -0.1246 | 1 |  |  | Deamidated (NQ)@4;  Deamidated (NQ)@14 | MLMQYLAFMGGKAQAEGR |
| 168 | 185 | 2017.82 | 2016.81 | 2016.96 | -0.1441 | 1 |  |  | Oxidation (M)@1 | MLMQYLAFMGGKAQAEGR |
| 871 | 887 | 2075.01 | 2074.00 | 2074.06 | -0.0605 | 1 |  |  | Deamidated (NQ)@3;  Deamidated (NQ)@5;  Oxidation (M)@11 | KLQLQLQDLQMQLNDTK |
| 548 | 564 | 2097.00 | 2095.99 | 2096.03 | -0.0444 | 0 |  |  | Carbamidomethyl (C)@2;  Deamidated (NQ)@10 | LCIVMHVTYQTDLFLDK |
| 1192 | 1210 | 2200.10 | 2199.09 | 2199.09 | -0.0001 | 1 |  |  | Deamidated (NQ)@1;  Deamidated (NQ)@2;  Oxidation (M)@13 | QQLTAFVEGLYGMIRDNVK |
| 991 | 1008 | 2211.11 | 2210.10 | 2210.05 | 0.0524 | 1 |  |  | Deamidated (NQ)@1;  Deamidated (NQ)@2 | QQAMRTRPDNMPLLNMHR |
| 610 | 628 | 2225.13 | 2224.12 | 2224.13 | -0.0077 | 0 |  |  | Gln->pyro-Glu (N-term Q)@N  _term;  Deamidated (NQ)@1;  Deamidated (NQ)@2;  Deamidated (NQ)@4 | QQLQSLLETLSAIEPHYIR |
| 610 | 628 | 2239.14 | 2238.13 | 2238.20 | -0.0673 | 0 |  |  | - | QQLQSLLETLSAIEPHYIR |
| 958 | 978 | 2383.96 | 2382.96 | 2383.08 | -0.1211 | 1 |  |  | Deamidated (NQ)@7;  Deamidated (NQ)@9;  Deamidated (NQ)@16 | ATDAESQINELKSMMQSLQEK |
| 1424 | 1444 | 2476.14 | 2475.14 | 2475.22 | -0.0846 | 0 |  |  | Deamidated (NQ)@3;  Deamidated (NQ)@4;  Deamidated (NQ)@9;  Deamidated (NQ)@10 | EFQNVVPPQQLLDNPAFQFLK |
| 19 | XP_003078192.1 | 654 | 660 | 805.42 | 804.42 | 804.42 | -0.0062 | 0 |  | 77 | - | EEVTSLK |
| 366 | 372 | 814.46 | 813.46 | 813.48 | -0.0270 | 1 |  |  | - | GLIRAER |
| 2269 | 2275 | 842.51 | 841.51 | 841.45 | 0.0510 | 0 |  |  | Gln->pyro-Glu (N-term Q)@N  _term | QITELQK |
| 2269 | 2275 | 860.49 | 859.48 | 859.47 | 0.0185 | 0 |  |  | Deamidated (NQ)@1 | QITELQK |
| 1841 | 1847 | 881.46 | 880.45 | 880.43 | 0.0191 | 0 |  |  | Deamidated (NQ)@4 | SFEQTIR |
| 2690 | 2698 | 930.53 | 929.53 | 929.49 | 0.0338 | 1 |  |  | Deamidated (NQ)@7 | GKVAEAQAR |
| 261 | 268 | 1026.50 | 1025.49 | 1025.45 | 0.0428 | 0 |  |  | Deamidated (NQ)@3 | TLNDEFMR |
| 2502 | 2510 | 1033.52 | 1032.51 | 1032.53 | -0.0246 | 0 |  |  | Deamidated (NQ)@1;  Deamidated (NQ)@5 | NTEVQTLVK |
| 17 | 25 | 1045.56 | 1044.56 | 1044.47 | 0.0830 | 0 |  |  | Deamidated (NQ)@3 | EPNEALESR |
| 1708 | 1715 | 1056.58 | 1055.58 | 1055.47 | 0.1016 | 0 |  |  | - | STMFWEQK |
| 270 | 279 | 1091.54 | 1090.53 | 1090.56 | -0.0286 | 1 |  |  | - | MKGVELGAMR |
| 2544 | 2553 | 1102.56 | 1101.55 | 1101.57 | -0.0154 | 0 |  |  | - | EAEALVDSLR |
| 645 | 653 | 1135.55 | 1134.55 | 1134.55 | 0.0020 | 1 |  |  | - | LESERSQMR |
| 2016 | 2025 | 1149.56 | 1148.55 | 1148.53 | 0.0185 | 1 |  |  | Deamidated (NQ)@5 | SASENEDKLR |
| 645 | 653 | 1151.55 | 1150.54 | 1150.54 | 0.0047 | 1 |  |  | Oxidation (M)@8 | LESERSQMR |
| 382 | 390 | 1183.65 | 1182.64 | 1182.53 | 0.1106 | 0 |  |  | Carbamidomethyl (C)@4 | YQSCLDLER |
| 694 | 703 | 1189.62 | 1188.62 | 1188.61 | 0.0071 | 1 |  |  | - | TELERALSDR |
| 2480 | 2490 | 1248.71 | 1247.70 | 1247.60 | 0.1033 | 1 | 0 |  | Deamidated (NQ)@4 | [AEANESRLTEK](../../%C3%A6%C2%A1%C5%92%C3%A9%C2%9D%C2%A2/%C3%A6%C2%A1%C5%92%C3%A9%C2%9D%C2%A2%C3%A6%C2%9D%C2%90%C3%A6%E2%80%93%E2%84%A2/%C3%A8%C2%B4%C2%A8%C3%A8%C2%B0%C2%B1%C3%A9%E2%80%B0%C2%B4%C3%A5%C2%AE%C5%A1%C3%A7%5C%C2%BB%E2%80%9C%C3%A6%C2%9E%C5%93/F12FTSECKJ0265_GS_20121211_489/F12FTSECKJ0265_GS_20121211_489/F12FTSECKJ0265_GS_20121211_489/PMF_Report/peptides/19-4.html#32) |
| 1529 | 1539 | 1292.63 | 1291.62 | 1291.63 | -0.0144 | 1 |  |  | Oxidation (M)@9 | ELDELKAEMAK |
| 946 | 957 | 1304.66 | 1303.66 | 1303.69 | -0.0301 | 0 |  |  | - | VTELSSVVDDLK |
| 1419 | 1429 | 1310.63 | 1309.63 | 1309.53 | 0.1003 | 1 |  |  | Deamidated (NQ)@6 | KSSEENTDDER |
| 1997 | 2007 | 1329.64 | 1328.63 | 1328.58 | 0.0538 | 0 |  |  | - | ETEEGYEMTIK |
| 2355 | 2365 | 1345.69 | 1344.68 | 1344.67 | 0.0152 | 1 |  |  | Deamidated (NQ)@2;  Deamidated (NQ)@8 | EQLRLDANAWK |
| 634 | 644 | 1365.65 | 1364.64 | 1364.65 | -0.0068 | 1 |  |  | - | FEAERLEMNAR |
| 2347 | 2358 | 1430.77 | 1429.76 | 1429.75 | 0.0077 | 1 |  |  | - | LSTLEADREQLR |
| 495 | 507 | 1432.73 | 1431.72 | 1431.70 | 0.0168 | 1 |  |  | - | ALLDSEMDKTGPR |
| 495 | 507 | 1448.79 | 1447.78 | 1447.70 | 0.0827 | 1 | 2.59 |  | Oxidation (M)@7 | [ALLDSEMDKTGPR](../../%C3%A6%C2%A1%C5%92%C3%A9%C2%9D%C2%A2/%C3%A6%C2%A1%C5%92%C3%A9%C2%9D%C2%A2%C3%A6%C2%9D%C2%90%C3%A6%E2%80%93%E2%84%A2/%C3%A8%C2%B4%C2%A8%C3%A8%C2%B0%C2%B1%C3%A9%E2%80%B0%C2%B4%C3%A5%C2%AE%C5%A1%C3%A7%5C%C2%BB%E2%80%9C%C3%A6%C2%9E%C5%93/F12FTSECKJ0265_GS_20121211_489/F12FTSECKJ0265_GS_20121211_489/F12FTSECKJ0265_GS_20121211_489/PMF_Report/peptides/19-4.html#48) |
| 1836 | 1847 | 1461.78 | 1460.77 | 1460.73 | 0.0487 | 1 |  |  | Gln->pyro-Glu (N-term Q)@N  _term;  Deamidated (NQ)@1 | QNLNKSFEQTIR |
| 796 | 808 | 1467.71 | 1466.71 | 1466.78 | -0.0765 | 1 | 0 |  | Gln->pyro-Glu (N-term Q)@N _erm | [QLADRAVIESNLR](../../%C3%A6%C2%A1%C5%92%C3%A9%EF%86%9D%C2%A2/%C3%A6%C2%A1%C5%92%C3%A9%EF%86%9D%C2%A2%C3%A6%EF%86%9D%EF%86%90%C3%A6%E2%80%93%E2%84%A2/%C3%A8%C2%B4%C2%A8%C3%A8%C2%B0%C2%B1%C3%A9%E2%80%B0%C2%B4%C3%A5%C2%AE%C5%A1%C3%A7%5C%C2%BB%E2%80%9C%C3%A6%C2%9E%C5%93/F12FTSECKJ0265_GS_20121211_489/F12FTSECKJ0265_GS_20121211_489/F12FTSECKJ0265_GS_20121211_489/PMF_Report/peptides/19-4.html#50) |
| 1836 | 1847 | 1481.70 | 1480.69 | 1480.70 | -0.0102 | 1 |  |  | Deamidated (NQ)@1;  Deamidated (NQ)@2;  Deamidated (NQ)@4;  Deamidated (NQ)@9 | QNLNKSFEQTIR |
| 1556 | 1568 | 1483.71 | 1482.71 | 1482.77 | -0.0618 | 1 |  |  | Gln->pyro-Glu (N-term Q)@N_term;  Deamidated (NQ)@1;  Deamidated (NQ)@11 | QLADRAIIESNLR |
| 796 | 808 | 1486.76 | 1485.75 | 1485.78 | -0.0290 | 1 |  |  | Deamidated (NQ)@1;  Deamidated (NQ)@11 | QLADRAVIESNLR |
| 280 | 294 | 1489.70 | 1488.70 | 1488.68 | 0.0160 | 0 |  |  | Deamidated (NQ)@1 | QEAVQGAAAGEQSSR |
| 1914 | 1925 | 1493.74 | 1492.73 | 1492.72 | 0.0152 | 1 |  |  | Deamidated (NQ)@3;  Deamidated (NQ)@6 | VSQAEQLFREER |
| 2438 | 2449 | 1499.71 | 1498.70 | 1498.80 | -0.0973 | 1 |  |  | Deamidated (NQ)@9 | LEEELVVRQLDR |
| 613 | 627 | 1510.81 | 1509.80 | 1509.71 | 0.0888 | 1 |  |  | - | ARSEIASMGTSDSAK |
| 1823 | 1835 | 1544.79 | 1543.78 | 1543.81 | -0.0244 | 1 |  |  | Deamidated (NQ)@5 | ERLAQLESELVEK |
| 2603 | 2616 | 1583.80 | 1582.79 | 1582.77 | 0.0186 | 1 |  |  | Deamidated (NQ)@12 | LDTVTSEYKEAQAK |
| 1848 | 1861 | 1594.77 | 1593.76 | 1593.73 | 0.0330 | 1 |  |  | Oxidation (M)@6 | DLKSSMTNVEDSPR |
| 1630 | 1644 | 1613.80 | 1612.79 | 1612.82 | -0.0315 | 0 |  |  | Gln->pyro-Glu (N-term Q)@N _erm;  Deamidated (NQ)@1 | QLTEAVAELQSLAEK |
| 1226 | 1239 | 1617.80 | 1616.80 | 1616.85 | -0.0552 | 1 |  |  | Deamidated (NQ)@6;  Deamidated (NQ)@13 | TLLEEQSKLLGEQK |
| 1296 | 1309 | 1623.80 | 1622.79 | 1622.80 | -0.0046 | 1 |  |  | Deamidated (NQ)@3;  Deamidated (NQ)@8;  Oxidation (M)@13 | AAQIDFEQRVVAMK |
| 256 | 268 | 1626.77 | 1625.76 | 1625.74 | 0.0273 | 1 |  |  | Oxidation (M)@12 | NDDLKTLNDEFMR |
| 958 | 971 | 1707.86 | 1706.85 | 1706.83 | 0.0250 | 1 |  |  | Deamidated (NQ)@2;  Deamidated (NQ)@4;  Oxidation (M)@13 | SQLQNRTTLEVNMR |
| 1096 | 1109 | 1724.93 | 1723.93 | 1723.83 | 0.0918 | 0 |  |  | - | DMTQLNEYVIEIEK |
| 1096 | 1109 | 1741.95 | 1740.94 | 1740.81 | 0.1304 | 0 |  |  | Oxidation (M)@2;  Deamidated (NQ)@4 | DMTQLNEYVIEIEK |
| 715 | 729 | 1746.90 | 1745.89 | 1745.91 | -0.0137 | 1 |  |  | Deamidated (NQ)@2 | LNATLRAAQIDFEQR |
| 2699 | 2713 | 1762.86 | 1761.86 | 1761.85 | 0.0029 | 1 |  |  | Deamidated (NQ)@7;  Deamidated (NQ)@12;  Deamidated (NQ)@14 | ADDAERQLFGLQLQR |
| 887 | 900 | 1770.84 | 1769.84 | 1769.86 | -0.0250 | 1 |  |  | Deamidated (NQ)@6 | KELEHQMSLLEEQR |
| 2339 | 2354 | 1788.86 | 1787.85 | 1787.96 | -0.1118 | 1 | 0 |  | - | [NLSELLSKLSTLEADR](../../%C3%A6%C2%A1%C5%92%C3%A9%EF%86%9D%C2%A2/%C3%A6%C2%A1%C5%92%C3%A9%C2%9D%C2%A2%C3%A6%C2%9D%C2%90%C3%A6%E2%80%93%E2%84%A2/%C3%A8%C2%B4%C2%A8%C3%A8%C2%B0%C2%B1%C3%A9%E2%80%B0%C2%B4%C3%A5%C2%AE%C5%A1%C3%A7%5C%C2%BB%E2%80%9C%C3%A6%C2%9E%C5%93/F12FTSECKJ0265_GS_20121211_489/F12FTSECKJ0265_GS_20121211_489/F12FTSECKJ0265_GS_20121211_489/PMF_Report/peptides/19-4.html#77) |
| 2528 | 2543 | 1804.92 | 1803.91 | 1803.95 | -0.0385 | 1 |  |  | Deamidated (NQ)@5 | TLAYNEEVQAAVLRAR |
| 661 | 675 | 1828.89 | 1827.88 | 1827.85 | 0.0301 | 1 |  |  | Oxidation (M)@6;  Deamidated (NQ)@13 | ADYDRMLLEAISQMR |
| 1753 | 1769 | 1940.96 | 1939.95 | 1940.06 | -0.1090 | 1 |  |  | - | LVRIQLAESIDNEAELK |
| 461 | 478 | 1962.95 | 1961.94 | 1961.97 | -0.0342 | 1 |  |  | Oxidation (M)@16 | ISEVRAEAEAEVNSLMAK |
| 2083 | 2100 | 1979.95 | 1978.94 | 1978.96 | -0.0175 | 1 |  |  | - | VEAEYAASTDSVTPDLRR |
| 1094 | 1109 | 2011.97 | 2010.96 | 2010.95 | 0.0151 | 1 |  |  | Deamidated (NQ)@6;  Deamidated (NQ)@8 | ERDMTQLNEYVIEIEK |
| 1094 | 1109 | 2027.97 | 2026.96 | 2026.94 | 0.0187 | 1 |  |  | Oxidation (M)@4;  Deamidated (NQ)@6;  Deamidated (NQ)@8 | ERDMTQLNEYVIEIEK |
| 1055 | 1071 | 2105.00 | 2103.99 | 2104.05 | -0.0549 | 1 |  |  | Oxidation (M)@8 | ELQEQLTMQSDLLERQK |
| 1851 | 1868 | 2110.01 | 2109.00 | 2108.88 | 0.1242 | 1 |  |  | Deamidated (NQ)@5;  Deamidated (NQ)@15 | SSMTNVEDSPRDDEQIER |
| 2391 | 2411 | 2120.98 | 2119.97 | 2119.98 | -0.0024 | 1 |  |  | Deamidated (NQ)@9;  Deamidated (NQ)@10 | SSEAESAANQEAAAKGEELVK |
| 1926 | 1944 | 2139.85 | 2138.85 | 2139.02 | -0.1702 | 1 |  |  | Gln->pyro-Glu (N-term Q)@N_term;  Deamidated (NQ)@1;  Deamidated (NQ)@5;  Deamidated (NQ)@12;  Deamidated (NQ)@16 | QALMNAHKIEVQTLENASR |
| 780 | 800 | 2225.12 | 2224.11 | 2224.17 | -0.0581 | 1 |  |  | - | SVPEAEVSAAIAELQKQLADR |
| 159 | 181 | 2290.06 | 2289.05 | 2289.14 | -0.0940 | 0 |  |  | Oxidation (M)@17;  Deamidated (NQ)@20 | AALPHAAVEGASEAIAMPPNVTR |
| 1569 | 1592 | 2521.33 | 2520.32 | 2520.19 | 0.1303 | 0 |  |  | Deamidated (NQ)@2;  Deamidated (NQ)@7 | AQIASVQQSLESLQASSSDTNAQR |
| 3 | XP_003063693.1 | 2536 | 2541 | 747.35 | 746.34 | 746.34 | -0.0041 | 0 |  | 79.4 | Deamidated (NQ)@1;  Deamidated (NQ)@2 | NQLDQK |
| 3337 | 3343 | 790.40 | 789.39 | 789.41 | -0.0105 | 0 |  |  | Deamidated (NQ)@2 | GNQVMIK |
| 2906 | 2913 | 842.50 | 841.49 | 841.54 | -0.0445 | 1 |  |  | - | QVVVAKAK |
| 3105 | 3112 | 860.44 | 859.44 | 859.44 | -0.0043 | 0 |  |  | Deamidated (NQ)@1 | QAALTEAR |
| 3829 | 3836 | 870.53 | 869.52 | 869.52 | 0.0044 | 1 |  |  | - | GLRANLAR |
| 402 | 408 | 896.47 | 895.46 | 895.49 | -0.0285 | 1 |  |  | Deamidated (NQ)@2;  Deamidated (NQ)@4 | VQHNRLK |
| 3794 | 3800 | 927.49 | 926.48 | 926.44 | 0.0421 | 0 |  |  | - | QPHENFR |
| 696 | 703 | 960.55 | 959.54 | 959.54 | -0.0016 | 1 |  |  | - | RSLQELSK |
| 3641 | 3649 | 999.52 | 998.51 | 998.47 | 0.0439 | 0 |  |  | - | NAEPESPQK |
| 2613 | 2620 | 1017.53 | 1016.53 | 1016.45 | 0.0725 | 0 |  |  | Oxidation (M)@4;  Carbamidomethyl (C)@7 | DALMHVCR |
| 1874 | 1882 | 1109.58 | 1108.58 | 1108.51 | 0.0620 | 0 |  |  | Oxidation (M)@2;  Deamidated (NQ)@3;  Oxidation (M)@7 | TMQDLIMPK |
| 3819 | 3828 | 1117.53 | 1116.52 | 1116.58 | -0.0666 | 1 |  |  | Oxidation (M)@4 | GLKMTTEPPK |
| 1495 | 1504 | 1148.61 | 1147.60 | 1147.59 | 0.0157 | 0 |  |  | - | SLYATLEHSK |
| 537 | 546 | 1202.62 | 1201.61 | 1201.58 | 0.0257 | 0 |  |  | - | ILEALDDEER |
| 1373 | 1382 | 1213.58 | 1212.57 | 1212.57 | -0.0001 | 1 |  |  | Oxidation (M)@7 | LDKFTDMEAK |
| 1421 | 1430 | 1230.64 | 1229.63 | 1229.54 | 0.0917 | 0 |  |  | Oxidation (M)@3;  Deamidated (NQ)@4;  Deamidated (NQ)@6 | DPMNVQPHFK |
| 802 | 811 | 1250.62 | 1249.61 | 1249.59 | 0.0216 | 0 |  |  | Deamidated (NQ)@2 | TQSFIAYYEK |
| 1332 | 1342 | 1255.63 | 1254.62 | 1254.63 | -0.0076 | 0 |  |  | Deamidated (NQ)@6 | MFDAVNVAFIK |
| 3105 | 3116 | 1282.61 | 1281.60 | 1281.70 | -0.1000 | 1 |  |  | Gln->pyro-Glu (N-term Q)@N _rm | QAALTEARAQLK |
| 1946 | 1958 | 1286.67 | 1285.66 | 1285.64 | 0.0183 | 1 |  |  | Deamidated (NQ)@3;  Oxidation (M)@4 | HGNMIVGKTGAGK |
| 866 | 877 | 1324.70 | 1323.70 | 1323.81 | -0.1156 | 1 |  |  | - | IDVGPLKQTLLK |
| 3975 | 3985 | 1365.66 | 1364.65 | 1364.61 | 0.0384 | 0 |  |  | Deamidated (NQ)@4;  Deamidated (NQ)@8;  Deamidated (NQ)@9 | DYIQTLPQNDR |
| 1846 | 1859 | 1463.76 | 1462.75 | 1462.83 | -0.0816 | 1 |  |  | - | SFVIPISRAAGAFK |
| 1495 | 1507 | 1480.75 | 1479.74 | 1479.74 | 0.0017 | 1 |  |  | Oxidation (M)@12 | SLYATLEHSKGMK |
| 3278 | 3289 | 1497.78 | 1496.77 | 1496.78 | -0.0069 | 1 |  |  | Deamidated (NQ)@4;  Oxidation (M)@5 | WIKNMEKPHGLK |
| 2360 | 2372 | 1506.74 | 1505.73 | 1505.80 | -0.0615 | 0 |  |  | - | SQFGFMPPLELLK |
| 297 | 308 | 1524.75 | 1523.74 | 1523.68 | 0.0602 | 1 | 0 |  | Deamidated (NQ)@9 | [ATRWHDDYNAFK](../../%C3%A6%C2%A1%C5%92%C3%A9%EF%86%9D%C2%A2/%C3%A6%C2%A1%C5%92%C3%A9%C2%9D%C2%A2%C3%A6%C2%9D%C2%90%C3%A6%E2%80%93%E2%84%A2/%C3%A8%C2%B4%C2%A8%C3%A8%C2%B0%C2%B1%C3%A9%E2%80%B0%C2%B4%C3%A5%C2%AE%C5%A1%C3%A7%5C%C2%BB%E2%80%9C%C3%A6%C2%9E%C5%93/F12FTSECKJ0265_GS_20121211_489/F12FTSECKJ0265_GS_20121211_489/F12FTSECKJ0265_GS_20121211_489/PMF_Report/peptides/3-6.html#41) |
| 2487 | 2500 | 1530.77 | 1529.76 | 1529.82 | -0.0651 | 1 |  |  | Deamidated (NQ)@7 | DLAKVIQGVMQATR |
| 3344 | 3356 | 1540.75 | 1539.74 | 1539.72 | 0.0222 | 1 |  |  | - | LGDKEVDYSPDFR |
| 3677 | 3691 | 1592.83 | 1591.83 | 1591.83 | -0.0060 | 1 |  |  | - | VEKAATNYVANSLGR |
| 1823 | 1836 | 1623.79 | 1622.78 | 1622.79 | -0.0077 | 0 |  |  | Oxidation (M)@1;  Deamidated (NQ)@9;  Deamidated (NQ)@10 | MVAIMELSQQQLSK |
| 586 | 598 | 1625.77 | 1624.76 | 1624.77 | -0.0039 | 1 | 0 |  | Carbamidomethyl (C)@2 | [HCKNAYESVVQYK](../../%C3%A6%C2%A1%C5%92%C3%A9%EF%86%9D%C2%A2/%C3%A6%C2%A1%C5%92%C3%A9%C2%9D%C2%A2%C3%A6%C2%9D%C2%90%C3%A6%E2%80%93%E2%84%A2/%C3%A8%C2%B4%C2%A8%C3%A8%C2%B0%C2%B1%C3%A9%E2%80%B0%C2%B4%C3%A5%C2%AE%C5%A1%C3%A7%5C%C2%BB%E2%80%9C%C3%A6%C2%9E%C5%93/F12FTSECKJ0265_GS_20121211_489/F12FTSECKJ0265_GS_20121211_489/F12FTSECKJ0265_GS_20121211_489/PMF_Report/peptides/3-6.html#49) |
| 3049 | 3062 | 1637.78 | 1636.77 | 1636.76 | 0.0088 | 0 |  |  | Deamidated (NQ)@3 | YVNDPEYQPDVIGK |
| 802 | 814 | 1641.78 | 1640.78 | 1640.81 | -0.0332 | 1 |  |  | - | TQSFIAYYEKTYK |
| 2965 | 2981 | 1655.82 | 1654.81 | 1654.90 | -0.0835 | 0 |  |  | Deamidated (NQ)@13 | AMPALAAAEAALNVLTK |
| 787 | 801 | 1657.79 | 1656.79 | 1656.88 | -0.0965 | 0 |  |  | Deamidated (NQ)@1;  Deamidated (NQ)@7;  Deamidated (NQ)@10 | QIVSIDNGINLIVEK |
| 1356 | 1372 | 1689.79 | 1688.78 | 1688.78 | 0.0004 | 1 |  |  | Carbamidomethyl (C)@2;  Deamidated (NQ)@15 | ACTGSDPKAGTPVSNAR |
| 1003 | 1016 | 1697.86 | 1696.86 | 1696.79 | 0.0657 | 1 |  |  | - | TVDAFTHDVDEHRR |
| 2346 | 2359 | 1741.86 | 1740.86 | 1740.86 | -0.0010 | 1 | 0 |  | - | [KMLVYIDDFNMPQK](../../%C3%A6%C2%A1%C5%92%C3%A9%C2%9D%C2%A2/%C3%A6%C2%A1%C5%92%C3%A9%C2%9D%C2%A2%C3%A6%C2%9D%C2%90%C3%A6%E2%80%93%E2%84%A2/%C3%A8%C2%B4%C2%A8%C3%A8%C2%B0%C2%B1%C3%A9%E2%80%B0%C2%B4%C3%A5%C2%AE%C5%A1%C3%A7%5C%C2%BB%E2%80%9C%C3%A6%C2%9E%C5%93/F12FTSECKJ0265_GS_20121211_489/F12FTSECKJ0265_GS_20121211_489/F12FTSECKJ0265_GS_20121211_489/PMF_Report/peptides/3-6.html#62) |
| 968 | 983 | 1755.87 | 1754.86 | 1754.96 | -0.0935 | 1 |  |  | - | LDGLEKVFDAHVATLK |
| 2346 | 2359 | 1757.86 | 1756.86 | 1756.85 | 0.0023 | 1 |  |  | Oxidation (M)@2 | KMLVYIDDFNMPQK |
| 2346 | 2359 | 1773.86 | 1772.85 | 1772.85 | 0.0058 | 1 |  |  | Oxidation (M)@2;  Oxidation (M)@11 | KMLVYIDDFNMPQK |
| 1920 | 1934 | 1803.88 | 1802.88 | 1802.88 | 0.0007 | 0 |  |  | Carbamidomethyl (C)@1;  Deamidated (NQ)@7 | CDELGLQVVDEWIVK |
| 488 | 501 | 1835.92 | 1834.91 | 1834.97 | -0.0619 | 1 |  |  | - | ELLHLFTEVRFFER |
| 4267 | 4282 | 1870.92 | 1869.91 | 1869.94 | -0.0280 | 1 |  |  | - | TGSRERPSFMTFVDLK |
| 2624 | 2642 | 1882.00 | 1881.00 | 1881.03 | -0.0297 | 1 |  |  | Deamidated (NQ)@4 | VLMQPRGNALLVGVGGSGR |
| 2491 | 2506 | 1887.93 | 1886.92 | 1886.92 | -0.0003 | 1 |  |  | Deamidated (NQ)@3 | VIQGVMQATRQYYDSK |
| 3265 | 3280 | 1896.02 | 1895.01 | 1894.98 | 0.0291 | 1 |  |  | Deamidated (NQ)@8;  Deamidated (NQ)@10;  Deamidated (NQ)@12 | WPLLIDPQGQGNKWIK |
| 3137 | 3152 | 1901.94 | 1900.93 | 1900.93 | 0.0040 | 1 |  |  | Deamidated (NQ)@1;  Deamidated (NQ)@12 | QALEDELADLEQKLER |
| 2491 | 2506 | 1904.95 | 1903.95 | 1903.90 | 0.0469 | 1 |  |  | Deamidated (NQ)@3;  Oxidation (M)@6;  eamidated (NQ)@7 | VIQGVMQATRQYYDSK |
| 3069 | 3085 | 1993.98 | 1992.98 | 1992.97 | 0.0063 | 0 |  |  | Carbamidomethyl (C)@3 | GLCQWVHAMFIYGNVAK |
| 1108 | 1125 | 2034.97 | 2033.97 | 2034.02 | -0.0492 | 1 |  |  | Deamidated (NQ)@11 | FKDIDVEELENASQIVGK |
| 4074 | 4090 | 2044.05 | 2043.04 | 2043.14 | -0.1043 | 1 | 0 |  | Deamidated (NQ)@2;  Deamidated (NQ)@6 | [YNILINNMLTTLHLLKK](../../%C3%A6%C2%A1%C5%92%C3%A9%C2%9D%C2%A2/%C3%A6%C2%A1%C5%92%C3%A9%C2%9D%C2%A2%C3%A6%C2%9D%C2%90%C3%A6%E2%80%93%E2%84%A2/%C3%A8%C2%B4%C2%A8%C3%A8%C2%B0%C2%B1%C3%A9%E2%80%B0%C2%B4%C3%A5%C2%AE%C5%A1%C3%A7%5C%C2%BB%E2%80%9C%C3%A6%C2%9E%C5%93/F12FTSECKJ0265_GS_20121211_489/F12FTSECKJ0265_GS_20121211_489/F12FTSECKJ0265_GS_20121211_489/PMF_Report/peptides/3-6.html#81) |
| 4074 | 4090 | 2060.02 | 2059.01 | 2059.14 | -0.1284 | 1 |  |  | Deamidated (NQ)@2;  Deamidated (NQ)@6;  Oxidation (M)@8 | YNILINNMLTTLHLLKK |
| 1837 | 1853 | 2110.07 | 2109.07 | 2109.06 | 0.0015 | 1 |  |  | - | QDHYDYTLRSFVIPISR |
| 1690 | 1707 | 2127.09 | 2126.08 | 2125.97 | 0.1082 | 1 |  |  | Deamidated (NQ)@6;  Carbamidomethyl (C)@7;  Oxidation (M)@15 | YVIVFNCSDGVDYKMTAK |
| 1254 | 1273 | 2157.08 | 2156.07 | 2156.02 | 0.0544 | 0 |  |  | Deamidated (NQ)@12 | STDDVFAALEDNVVTLSTMK |
| 2393 | 2413 | 2221.13 | 2220.12 | 2220.04 | 0.0783 | 1 |  |  | Deamidated (NQ)@3;  Oxidation (M)@5 | DIQLMASMAPPGGGRNAFSQR |
| 3568 | 3585 | 2225.12 | 2224.11 | 2224.11 | 0.0061 | 0 |  |  | Deamidated (NQ)@3;  Deamidated (NQ)@6 | ILQSSNQVNLEEWQFFLK |
| 3357 | 3375 | 2239.14 | 2238.13 | 2238.16 | -0.0223 | 1 |  |  | Deamidated (NQ)@9 | LYLTSKLFNPHYTPEVSTK |
| 847 | 865 | 2257.09 | 2256.08 | 2256.06 | 0.0165 | 0 |  |  | Deamidated (NQ)@11;  Oxidation (M)@15 | ELFVEVEGEDQITNMAFLR |
| 2944 | 2964 | 2260.05 | 2259.04 | 2259.07 | -0.0282 | 1 |  |  | Carbamidomethyl (C)@15 | IEKEAEEANAIAAECQAGLDK |
| 2989 | 3009 | 2301.18 | 2300.17 | 2300.30 | -0.1256 | 1 |  |  | Carbamidomethyl (C)@12 | AYAKPPALVELCLKGVMTVLK |
| 3363 | 3384 | 2505.35 | 2504.34 | 2504.33 | 0.0069 | 1 |  |  | Deamidated (NQ)@3 | LFNPHYTPEVSTKVTIVNFAVK |
| 2281 | 2307 | 2838.28 | 2837.27 | 2837.47 | -0.1927 | 1 |  |  | Deamidated (NQ)@7;  Oxidation (M)@14;  Deamidated (NQ)@17;  Carbamidomethyl (C)@19 | HVLIIGNVGVGKTMVAQSCLEALPEGK |
| 4177 | 4203 | 3068.61 | 3067.60 | 3067.55 | 0.0539 | 1 |  |  | - | NAIPIDTLSFEYSIVNAEEKEVHQPPK |

The peptides with ion scores were identified by MS/MS. The protein scores are deribved from PMF matching. @ shows the modification sites of amino acid.
